# Identification of the pheromone biosynthesis genes from the sex pheromone gland transcriptome of the diamondback moth, *Plutella xylostella*

**DOI:** 10.1038/s41598-017-16518-8

**Published:** 2017-11-24

**Authors:** Da-Song Chen, Jian-Qing Dai, Shi-Chou Han

**Affiliations:** Guangdong Key Laboratory of Animal Conservation and Resource Utilization, Guangdong Public Laboratory of Wild Animal Conservation and Utilization, Guangdong Institute of Applied Biological Resources, Guangzhou, China

## Abstract

The diamondback moth was estimated to increase costs to the global agricultural economy as the global area increase of *Brassica* vegetable crops and oilseed rape. Sex pheromones traps are outstanding tools available in Integrated Pest Management for many years and provides an effective approach for DBM population monitoring and control. The ratio of two major sex pheromone compounds shows geographical variations. However, the limitation of our information in the DBM pheromone biosynthesis dampens our understanding of the ratio diversity of pheromone compounds. Here, we constructed a transcriptomic library from the DBM pheromone gland and identified genes putatively involved in the fatty acid biosynthesis, pheromones functional group transfer, and β-oxidation enzymes. In addition, odorant binding protein, chemosensory protein and pheromone binding protein genes encoded in the pheromone gland transcriptome, suggest that female DBM moths may receive odors or pheromone compounds via their pheromone gland and ovipositor system. Tissue expression profiles further revealed that two ALR, three DES and one FAR5 genes were pheromone gland tissue biased, while some chemoreception genes expressed extensively in PG, pupa, antenna and legs tissues. Finally, the candidate genes from large-scale transcriptome information may be useful for characterizing a presumed biosynthetic pathway of the DBM sex pheromone.

## Introduction

We have invested significant time in studying the diamondback moth (DBM), *Plutella xylostella* (Lepidoptera: Plutellidae), and its ability to block the serious threat posed to *Brassica* vegetable crops and canola production. However, recently DBM was estimated to increase costs to the global economy by as much as US $4–5 billion annually^1^ since the global area of *Brassica* vegetable crops and oilseed rape has increased^2^.

Synthetic insecticides are the most routinely widespread agents in the control of DBM populations. However, DBM has developed resistance to all major classes of synthetic insecticides^3^ including the bacterial insecticide *Bacillus thuringiensis* (Bt) Cry toxin^4^. Moreover, widespread use of broad-spectrum insecticides leads to a striking absence of a range of effective natural enemies, especially parasitoids, which is believed to establish the status of DBM as a major vegetable crop pest^5,6^. The ability of DBM to migrate long distances, in contrast to no evidence indicating migration of any DBM-derived parasitoids, highlights the inherent challenge of managing DBM^7^.

The acceptance of the notion that more ecologically rational approaches to DBM population control should be carried out has resulted in an Integrated Pest Management (IPM) program for DPM. Sex pheromone traps have been figured quite prominently among the variety of potential tools available in IPM for many years^8,9^. And the attractants for DBM that are based on sex pheromones have also provided an effective approach for DBM population monitoring and control^10,11^.

Two sex pheromone compounds of DBM were identified, and defined as (*Z*)-11-hexadecenal (*Z*11-16:Ald) and (*Z*)-11-hexadecenyl acetate (*Z*11-16:OAc)^12,13^. (Z)-11-hexadecenol (Z11-16:OH)^14^ and (Z)-9-tetradecenyl acetate (Z9-14:OAc)^15^ display a synergistic effect on the relative attraction of a mixture of DBM sex pheromones. An equal mixture of *Z*11-16:Ald and *Z*11-16:OAc with a minimal amount of Z11-16:OH was reported to be highly attractive to DBM male moths in the field^13,16^. The attraction to DBM male moths is reported to vary among the major compounds of *Z*11-16:Ald and *Z*11-16:OAc^17,18^. The ratio of the major sex pheromone compounds used in the lure mixture is not coincident in different locations, which suggests that different DBM populations display geographical variation in the ratio of sex pheromone compounds^7,17,19^. However, the limitation of our knowledge in the context of fatty acid and sex pheromone biosynthesis of DBM, dampens our understanding of the ratio diversity of sex pheromones. Characterization of the enzymes that are involved in pheromone biosynthesis provides an avenue to understand the evolution of DBM sexual communication.

Female lepidopterans usually produce species-specific sex pheromones as multi-component blends with a precise ratio^20^. Most lepidopteran species utilize Type I pheromone blends that usually comprise straight-chain compounds of 10–18 carbons in length with several double bonds and displaying an oxygenated functional group of a primary alcohol, aldehyde, or acetate ester^21^. Small numbers of lepidopterans utilize Type II pheromones that are biosynthesized from diet-derived linoleic or linolenic acids (i.e., 1–3 *cis* hydrocarbons and 0–2 epoxide functions)^22^.

A general scheme has become apparent for the *de novo* biosynthetic pathway of Type I pheromone components in the pheromone gland (PG)^23^. Like other fatty acids and the *de novo* biosynthesis in a variety of biological systems, the carbon atoms of Type I sex pheromones are derived from acetyl-CoA. Acetyl-CoA carboxylase (AAC) catalyzes the biotin-dependent carboxylation of acetyl-CoA to form the saturated fatty-acid precursor, malonyl-CoA^24^. Then, the fatty acid synthetase (FAS) enzyme catalyzes the synthesis of the acyl chain from malonyl-CoA through chain elongation by 2-carbon units. Double bonds are generally introduced into the acyl chain by specific desaturases (DESs) in Δ5^25^, Δ6^26^, Δ9^27^, Δ10^28^, Δ11^29^, Δ12^30^ and Δ14^31^.

The DES gene of *Bombyx mori*, *desat1*, is capable of introducing double bonds in Δ11 producing mono-unsaturated fatty acids, or in Δ10 and Δ12, thus generating di-unsaturated fatty acids^30^. Di-unsaturated fatty acid can be also produced by two DES genes consecutively^26^. The acyl chain lengths of some unsaturated precursors can be adjusted by β-oxidation^32^ catabolic process to generate the full length pheromone precursor. The fatty acid reductases (FARs) are key biosynthesis enzymes in the synthesis of oxygenated functional groups, which convert fatty-acyl pheromone precursors to fatty alcohols^33^. The FAR gene can encode multi-substrate reductases^34^, and interplay with the pheromone fatty acyl precursors in shaping the ratiometric composition of pheromones^35,36^. Besides fatty alcohols serving as major pheromone components, the hydroxyl groups of fatty alcohols will be oxidized to aldehydes^37^ or esterified to acetoxy^38^ residues to form the actual functional groups of pheromones. The fatty aldehydes, which are compositionally major pheromones of some moths, are derived from fatty alcohols by alcohol dehydrogenase catalysis^39^.

The acetyl CoA fatty alcohol acetyltransferases are key enzymes that catalyze the formation of acetates by transferring the acetate group from acetyl-CoA to a fatty alcohol^40^. In addition, their roles in the modification of pheromone composition have been previously investigated^41^. Moreover, in addition to key enzymes that are directly involved in pheromone biosynthesis, some enzymes like fatty acid transport proteins (FATPs) and acyl-CoA binding proteins (ACBPs) also play crucial roles in the transport of long chain fatty acids^42^ or acyl-CoA esters^43^.

Finally, establishing EST-libraries by next generation sequencing technology (NGS) facilitates the investigation of candidate genes that might be potentially involved in pheromone biosynthesis^44–47^. Hence, we constructed a transcriptomic library from the sex pheromone gland of DBM and identified genes that could be putatively involved in the biosynthesis of sex pheromones, fatty acids, and β-oxidation enzymes. The tissue expression profiles of putative genes also provide novel insights into the biosynthetic sex pheromone pathway.

## Results and Discussion

### Illumina sequencing and transcriptome reconstruction

More than 56.6 million clean reads were obtained from the library of the DBM pheromone gland (PG) with about 8.5 G base-pairs of nucleotides, a 0.01% error rate and 92.62% bases with a Phred quality score of more than 30 (Q30). Compared to the DBM genome size of about 394 Mb^48^, clean data can provide an appropriate coverage of sequencing that satisfy the bioinformatics study.

**Figure 1:**
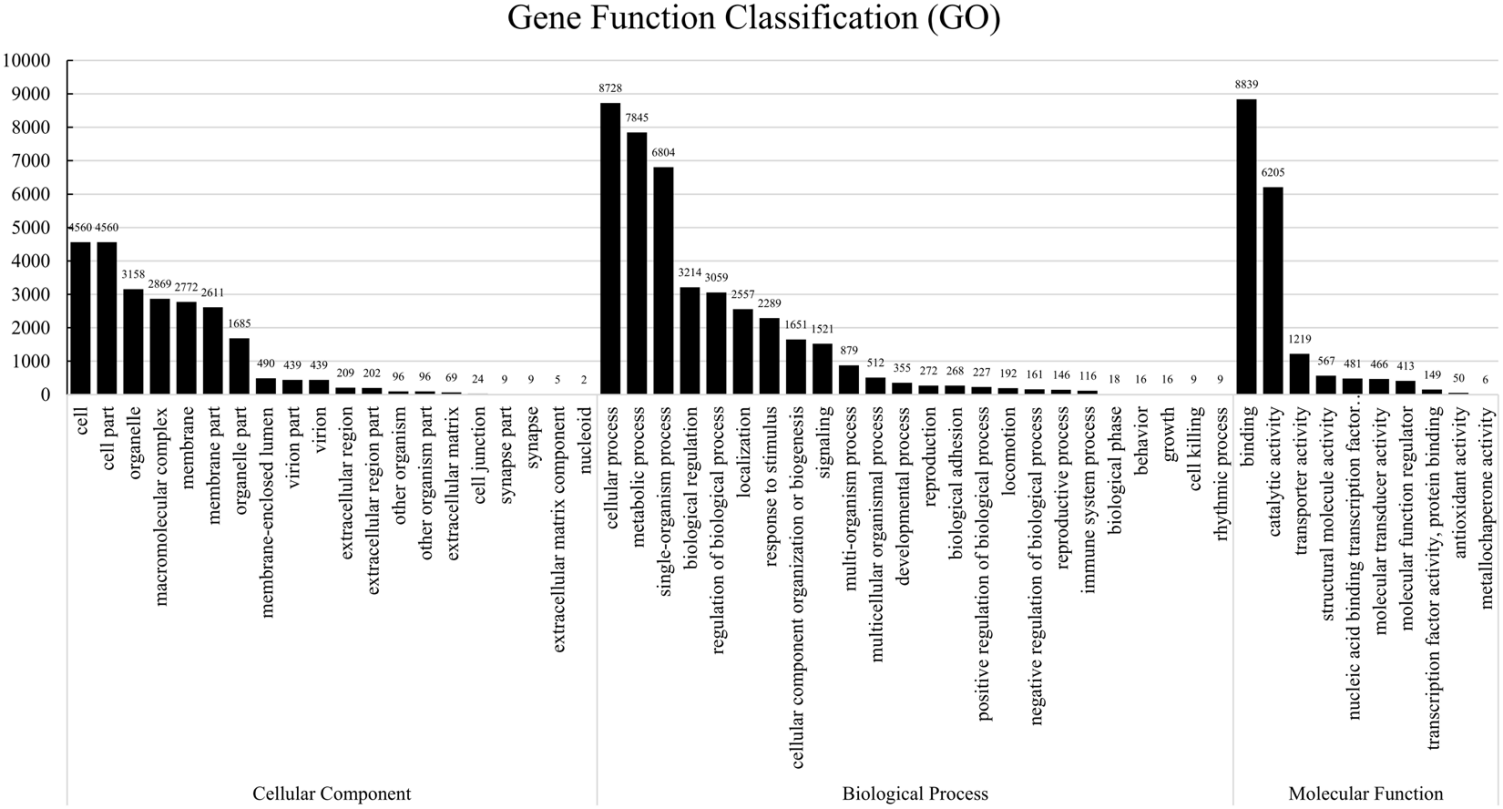
Histogram of gene ontology (GO) classification. Results are summarized for the three main GO categories: biological process, cellular component and molecular function. The number on the bars represents the total number of contigs in each category.

We first tried to map the sequences of clean data to the DBM genome maintained on the NCBI genomic database (GenBank: AHIO00000000.1); however, the overall alignment rate of the mapping results was low (56.49%) indicated a large number of clean data waste that could not be mapped to the reference genomic sequence. The high levels of DBM genomic heterozygosity and polymorphism^48^ most likely challenged the alignment algorithm. Therefore, the transcriptome was reconstructed to 73,769 contig sequences (>200 bp) by *de novo* assembly software to avoid excessive residual sequencing data. These consensus contigs have a mean length of 757 bp and an N50 length of 1314 bp with a total length of 55.83 Mb. Size distributions of the contigs are summarized in Figure S1. We defined these contigs as the sequences of genes although each of them may not necessarily represent a unique genomic sequence. Homology comparison of assembly sequences was performed by BlastX searching to the protein database NR (see Figs S2 and S3). The BlastX results were transferred to the Blast2Go program, which assigned the assembled transcripts to different functional categories (Fig. 1).

### Pheromone biosynthesis activating neuropeptide receptor

**Figure 2:**
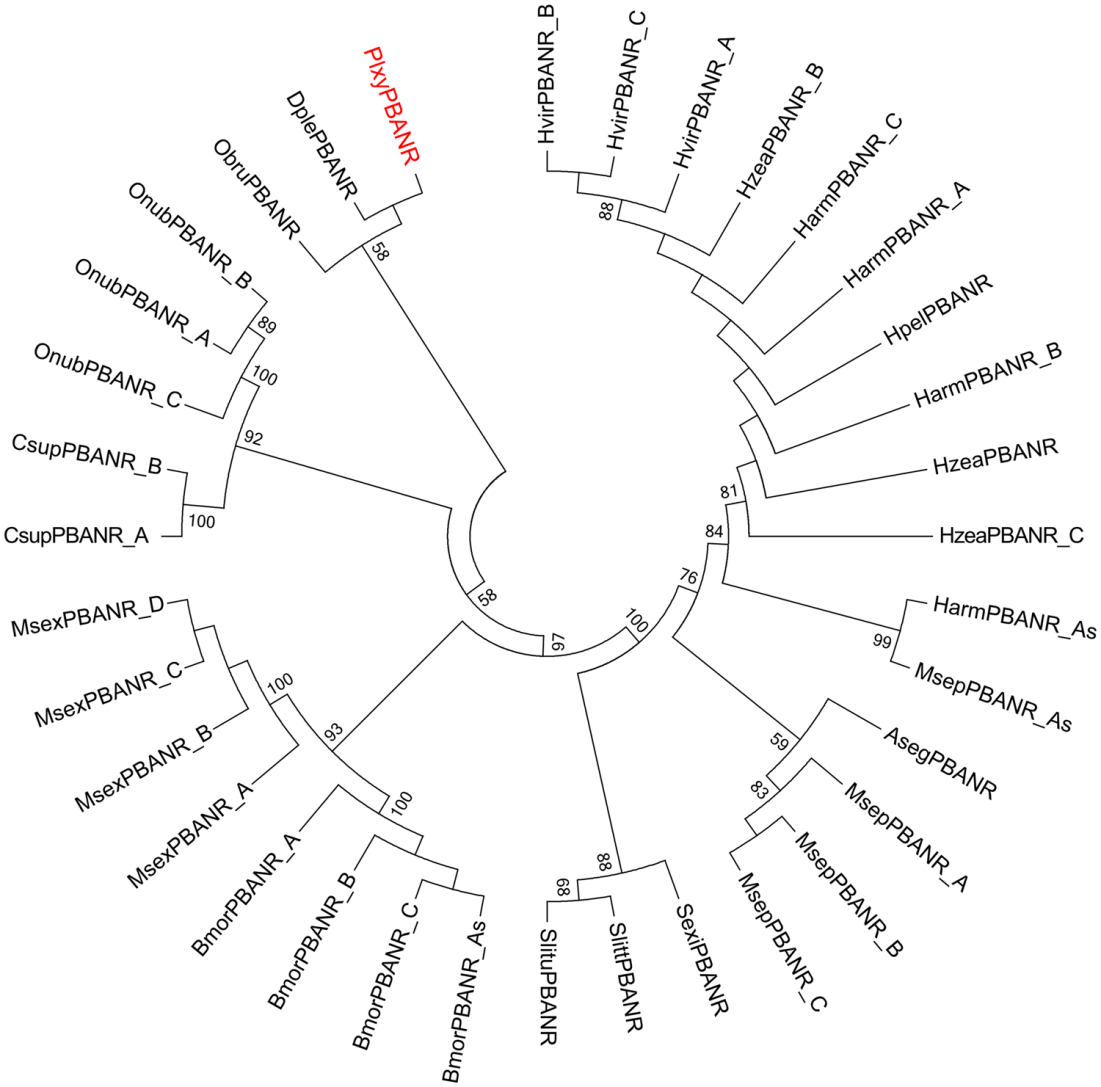
The phylogeny of PBAN receptors. The neighbor-joining (NJ) consensus tree of PBAN receptors was constructed using amino-acid sequences. Bootstrap values of NJ analyses are shown at the nodes as percent of total from 1000 bootstrap runs.

Pheromone biosynthesis activating neuropeptide (PBAN) is released from the suboesophageal ganglion and is transported through hemolymph to the PG. The binding of PBAN and its receptor in the PG membrane triggers sex pheromone production^49^. The PBAN receptor is characterized as a G-protein-coupled receptor and has been cloned in several species^50,51^. A transcript encoding a 338 aa protein, which encoded the same amino acid sequence with one DBM PBAN receptor deposited previously (AAY34744)^52^, was annotated as PBAN receptor (PxylPBANR) (Table 1). It has 78% identity to the *Bombyx mori* PBAN receptor isoform A in GenBank (AEX15646.1). The amino acid sequences of PxylPBANR and other PBAN receptors downloaded from GenBank were aligned and compared by the ClustalW method^53^. The final conserved amino acid sequences were adjusted to 328 aa in length using MEGA7^54^ after abandoning divergent regions (Supplementary Table S1). The evolutionary relationship was inferred using the Neighbor-Joining method^55^ and the evolutionary distances were computed using the JTT optimum method^56^ that was matrix-based with a gamma distribution. PxylPBANR was clustered together with DplePBANR (from *Danaus plexippus*) and ObruPBANR (from *Operophtera brumata*) (Fig. 2).

**Table 1:** BlastX match of transcripts involved in sex pheromone or fatty acid biosynthesis and β-oxidation. The EC numbers of the enzymes follow the enzyme name.

Gene id	Gene name	Gene Length	ORF	Accession Number	Putative identification	Species	Score(bits)	Expect value
PBAN receptor								
c46466_g1	PBANR	736	531	ACQ90219.1	pheromone biosynthesis-activating neuropeptide receptor subtype A	*Manduca sexta*	205	6E-63
ACC	EC 6.4.1.2							
c57656_g1	ACC	9310	7158	XP_013176189.1	acetyl-CoA carboxylase	*Papilio xuthus*	11036	0.0
FAS	EC 2.3.1.85							
c57640_g1	FAS	8353	7158	AGR49310.1	fatty acid synthase	*Agrotis ipsilon*	8953	0.0
DES	EC 1.14.19.5							
c51630_g2	DES1	1886	1062	AGR49313.1	acyl-CoA desaturase	*Agrotis ipsilon*	1589	0.0
c52870_g1	DES2	1606	1035	AII21943.1	desaturase	*Sesamia inferens*	1164	1e-155
c55325_g1	DES3	953	797	ALA65425.1	Z11-fatty acid desaturase	*Manduca sexta*	1030	2e-136
c53736_g1	DES4	2138	267	CAJ27975.1	acyl-CoA delta-9 desaturase	*Manduca sexta*	445	2e-51
c48732_g1	DES5	677	381	EHJ76461.1	acyl-CoA-delta9-3a-desaturase	*Danaus plexippus*	234	3e-73
c49569_g1	DES6	1600	1089	XP_013178743.1	stearoyl-CoA desaturase 5-like isoform X1	*Papilio xuthus*	1359	0.0
c54998_g1	DES7	1610	1083	ADP21588.1	fatty-acyl CoA Z/E11-desaturase	*Yponomeuta padellus*	541	0.0
c60875_g1	DES8	1852	1443	KNG52058.1	acyl-CoA desaturase	*Stemphylium lycopersici*	2518	0.0
c51467_g2	DES9	474	474	AAM28508.1	acyl-CoA desaturase PsepLPAQ	*Mythimna separata*	861	8e-113
c47747_g1	DES10	1230	996	XP_013181256.1	acyl-CoA Delta(11) desaturase	*Papilio xuthus*	1228	2e-165
c51467_g1	DES11	729	368	XP_014357114.1	acyl-CoA Delta(11) desaturase-like, partial	*Papilio machaon*	163	3e-12
c51630_g1	DES12	318	208	AGR49313.1	acyl-CoA desaturase NPVE	*Agrotis ipsilon*	256	2e-24
FAR	EC 1.2.1							
c52916_g1	FAR1	2517	1374	ADD62439.1	fatty-acyl CoA reductase II	*Yponomeuta evonymellus*	1334	8e-178
c55457_g1	FAR2	2371	1878	ADI82775.1	fatty-acyl CoA reductase 2	*Ostrinia nubilalis*	2746	0.0
c53808_g1	FAR3	2141	1623	ADD62440.1	fatty-acyl CoA reductase III	*Yponomeuta evonymellus*	1751	0.0
c56133_g1	FAR4	2402	1587	XP_014371693.1	fatty acyl-CoA reductase 1	*Papilio machaon*	2322	0.0
c55024_g1	FAR5	1217	1098	XP_004930778.1	putative fatty acyl-CoA reductase CG8306	*Bombyx mori*	1655	0.0
c53541_g1	FAR6	2547	1605	ALJ30235.1	putative fatty acyl reductase FAR1	*Spodoptera litura*	2124	0.0
c56693_g1	FAR7	1952	1593	XP_012545689.1	fatty acyl-CoA reductase 1-like	*Bombyx mori*	1219	5e-158
c56405_g2	FAR8	1776	1557	AKD01785.1	fatty acyl-CoA reductase 7	*Helicoverpa assulta*	1518	0.0
c56306_g1	FAR9	1876	1554	XP_012549536.1	putative fatty acyl-CoA reductase CG5065	*Bombyx mori*	2252	0.0
c56313_g3	FAR10	1747	1062	ADD62438.1	fatty-acyl CoA reductase I	*Yponomeuta evonymellus*	1408	0.0
c55072_g1	FAR11	1720	1407	ADD62439.1	fatty-acyl CoA reductase II	*Yponomeuta evonymellus*	1110	8e-144
c53406_g1	FAR12	936	690	ALJ30243.1	putative fatty acyl reductase FAR9	*Spodoptera litura*	861	4e-113
c52336_g1	FAR13	1001	814	ADI82777.1	fatty-acyl CoA reductase 4	*Ostrinia nubilalis*	1106	1e-145
c49015_g1	FAR14	2154	1491	XP_014366322.1	putative fatty acyl-CoA reductase CG5065	*Papilio machaon*	1968	0.0
c46565_g1	FAR15	1990	588	XP_013192592.1	fatty acyl-CoA reductase 1-like	*Amyelois transitella*	545	6e-64
ACT	EC:2.3.1							
c52455_g1	ACT1	1820	1269	XP_013192033.1	acetyl-CoA acetyltransferase mitochondrial isoform X3	*Amyelois transitella*	1901	0.0
c53185_g1	ACT2	2412	728	ALJ30248.1	acetyltransferase ACT1	*Spodoptera litura*	510	3e-60
ADH	EC 1.1.1.1							
c53443_g1	ADH1	1436	1131	AKQ06154.1	alcohol dehydrogenase AD8	*Cydia pomonella*	1828	0.0
c54850_g1	ADH2	1305	975	BAR64763.1	alcohol dehydrogenase	*Ostrinia furnacalis*	1377	0.0
c56088_g1	ADH3	5696	1041	AKD01749.1	alcohol dehydrogenase 14	*Helicoverpa assulta*	1474	0.0
c53167_g1	ADH4	562	553	AKD01746.1	alcohol dehydrogenase 7	*Helicoverpa assulta*	631	6e-78
c52742_g1	ADH5	657	478	AKD01725.1	alcohol dehydrogenase 3	*Helicoverpa armigera*	594	6e-73
ALR	EC 1.1.1.2							
c49161_g1	ALR1	1357	1032	XP_013136681.1	aldo-keto reductase AKR2E4-like	*Papilio polytes*	1188	4e-159
c53398_g1	ALR2	1170	1020	XP_013198587.1	aldo-keto reductase AKR2E4-like	*Amyelois transitella*	1121	2e-149
c55873_g1	ALR3	5216	1017	XP_013186405.1	aldo-keto reductase AKR2E4-like	*Amyelois transitella*	1050	2e-138
c50533_g1	ALR4	1120	1026	XP_004933321.1	aldo-keto reductase AKR2E4-like isoform X1	*Bombyx mori*	1325	6e-180
c49689_g1	ALR5	725	486	BAM19656.1	aldo-keto reductase AKR2E4-like	*Papilio xuthus*	228	3e-71
c49594_g1	ALR6	412	318	AII21974.1	aldo-ketose reductase 5	*Sesamia inferens*	330	1e-35
c43766_g1	ALR7	413	404	KOB65847.1	aldo-keto reductase	*Operophtera brumata*	138	1e-34
c53610_g1	ALR8	595	481	XP_013186405.1	aldo-keto reductase AKR2E4-like	*Amyelois transitella*	536	5e-64
c43090_g1	ALR9	386	207	AGQ45615.1	aldo-keto reductase	*Agrotis ipsilon*	284	2e-28
ACO	EC 1.3.3.6							
c57369_g1	ACO1	2696	2097	AID66678.1	peroxisomal acyl-CoA oxidase 3	*Agrotis segetum*	2859	0.0
c56807_g2	ACO2	891	888	XP_004932404.1	probable peroxisomal acyl-coenzyme A oxidase 1	*Bombyx mori*	1483	0.0
c56807_g1	ACO3	1012	660	AID66675.1	putative peroxisomal acyl-CoA oxidase	*Agrotis segetum*	822	6e-107
c51345_g1	ACO4	699	473	XP_013188650.1	probable peroxisomal acyl-coenzyme A oxidase 1	*Amyelois transitella*	520	9e-59
c56268_g1	ACO5	3652	2064	XP_013196118.1	peroxisomal acyl-coenzyme A oxidase 3	*Amyelois transitella*	2982	0.0
c56807_g3	ACO6	938	501	EHJ70241.1	putative acyl-CoA oxidase	*Danaus plexippus*	720	9e-92
c49764_g1	ACO7	423	423	XP_013188704.1	probable peroxisomal acyl-coenzyme A oxidase 1	*Amyelois transitella*	640	3e-76
c49768_g1	ACO8	499	498	AID66677.1	putative peroxisomal acyl-CoA oxidase 1	*Agrotis segetum*	719	3e-90
c49336_g1	ACO9	812	780	XP_013149592.1	probable peroxisomal acyl-coenzyme A oxidase 1	*Papilio polytes*	1093	1e-141
c52680_g1	ACO10	907	576	XP_014365999.1	probable peroxisomal acyl-coenzyme A oxidase 1	*Papilio machaon*	661	1e-78
c49336_g2	ACO11	356	352	XP_013188651.1	probable peroxisomal acyl-coenzyme A oxidase 1	*Amyelois transitella*	457	1e-50
c47685_g1	ACO12	1276	1241	XP_013177323.1	probable peroxisomal acyl-coenzyme A oxidase 1 isoform X1	*Papilio xuthus*	1673	0.0
c47971_g1	ACO13	324	323	EHJ66979.1	putative Acyl-coenzyme A oxidase 1	*Danaus plexippus*	316	8e-32
c40413_g1	ACO14	449	447	XP_013149571.1	probable peroxisomal acyl-coenzyme A oxidase 1	*Papilio polytes*	515	3e-58
ECH	EC 4.2.1.17							
c52123_g1	ECH1	1175	888	XP_013199717.1	probable enoyl-CoA hydratase, mitochondrial isoform X1	*Amyelois transitella*	545	0
c56605_g1	ECH2	2725	894	XP_013169198.1	enoyl-CoA hydratase domain-containing protein 2, mitochondrial	*Papilio xuthus*	485	1E-171
c53600_g1	ECH3	1282	1008	XP_013137975.1	probable enoyl-CoA hydratase	*Papilio polytes*	438	4E-152
c50688_g1	ECH4	1123	840	AID66690.1	enoyl-CoA hydratase domain-containing protein 3	*Agrotis segetum*	422	3E-147
HAD	EC 1.1.1.35							
c48555_g1	HAD1	1297	765	AID66692.1	3-hydroxyacyl-CoA dehydrogenase	*Agrotis segetum*	446	1E-157
c53767_g1	HAD2	869	627	XP_013140867.1	probable 3-hydroxyacyl-CoA dehydrogenase B0272.3 isoform X2	*Papilio polytes*	354	2E-121
c51812_g1	HAD3	1447	771	XP_013139736.1	3-hydroxyacyl-CoA dehydrogenase type-2-like	*Papilio polytes*	417	4E-146
c48984_g1	HAD4	432	258	XP_014361360.1	probable 3-hydroxyacyl-CoA dehydrogenase B0272.3 isoform	*Amyelois transitella*	160	3E-47
KAT								
c52501_g1	KAT1	1541	1200	XP_012546519.1	3-ketoacyl-CoA thiolase, mitochondrial-like	*Bombyx mori*	632	0.0

### Putative genes involved in pheromone biosynthesis

In most moth species, the precursors of Type I sex pheromones are synthesized as saturated long chain fatty acids^21,57–59^. In the transcriptome of DBM sex pheromone, we annotated contigs that encode for the following proteins: acetyl-CoA carboxylase (ACC, n = 1), fatty acid synthase (FAS, n = 1), desaturases (Des, n = 12), fatty-acyl reductase (FAR, n = 15), alcohol dehydrogenases (ADH, n = 5), aldo-keto reductase family 1 (ALR1, n = 9), acetyltransferase (ACT, n = 2) (Table 1), which involve in the *de novo* sex pheromone biosynthesis. The rate-limiting step in fatty acid biosynthesis is the first process, which catalyzes the ATP-dependent and biotin-dependent carboxylation of two acetyl-CoA to malonyl-CoA by ACC. One transcript with a high FPKM value of 213.21 possesses a large full-length open reading frame (ORF) encoding a protein of 2385 aa in length in the pheromone gland (PG) transcriptome of DBM (Table 1). It showed high sequence similarity to ACC as described in other insects (including *Bombyx mori*, *Drosophila melanogaster*, *Nasonia vitripennis*, *Tribolium castaneum*, *Papilio polytes* and *Helicoverpa armigera*) and shared 86% aa identity to *P. polytes*, *B. mori* and *H. armigera*.

Synthesis of saturated fatty acids from malonyl CoA, acetyl CoA and NADPH is catalyzed by a single, homodimeric, multifunctional protein known as FAS, in which acetyl-CoA undergoes a series of decarboxylation condensations with several malonyl residues^24,60^. A long transcript, which has a high expression level with a 634.68 FPKM value, was identified as FAS in the DBM PG transcriptome (Table 1). It was predicted to encode a large protein of 2385 aa in length with high sequence similarity to FAS annotated in other insects, and sharing 72% aa identity to *Agrotis ipsilon* and *Helicoverpa assulta*.

Double bonds are introduced into the fatty acid chain by a variety of desaturases at specific positions along the chain. Two sex pheromone compounds of DBM were identified as (*Z*)-11-hexadecenal (*Z*11-16:Ald) and (*Z*)-11-hexadecenyl acetate (*Z*11-16:OAc). It is reasonable to propose that DBM pheromone compounds would be desaturated by Δ11-desaturase from the saturated fatty acid precursor palmitic acid (16:0), which is supported by other studies in Lepidoptera species^61,62^. Not only being able to insert the double bond in the 11^th^ carbon of the fatty-acyl chain, desaturases can also insert in other locations such as Δ9^27^, Δ5^25^, Δ10^28^ and Δ14^31^.

For instance, Δ9-desaturases have been identified as two groups in pheromone glands of Lepidoptera species: one of which has a 16 carbon substrate chain length preference (C_16_ > C_18_) with the KPSE motif. By contrast, another with a chain length selectivity of 18 carbon substrate (C_18_ > C_16_) had the NPVE motif^63^. From the DBM PG transcriptome, twelve transcripts were identified as desaturase candidates (Table 1). The expression of Des1, Des2 and Des3 was high with FPKM values of 1107.92, 391.52 and 659.52 respectively. The amino acid sequences of Des genes from different species were aligned by using the MUSCLE method to demonstrate the relationship of desaturases.

**Figure 3:**
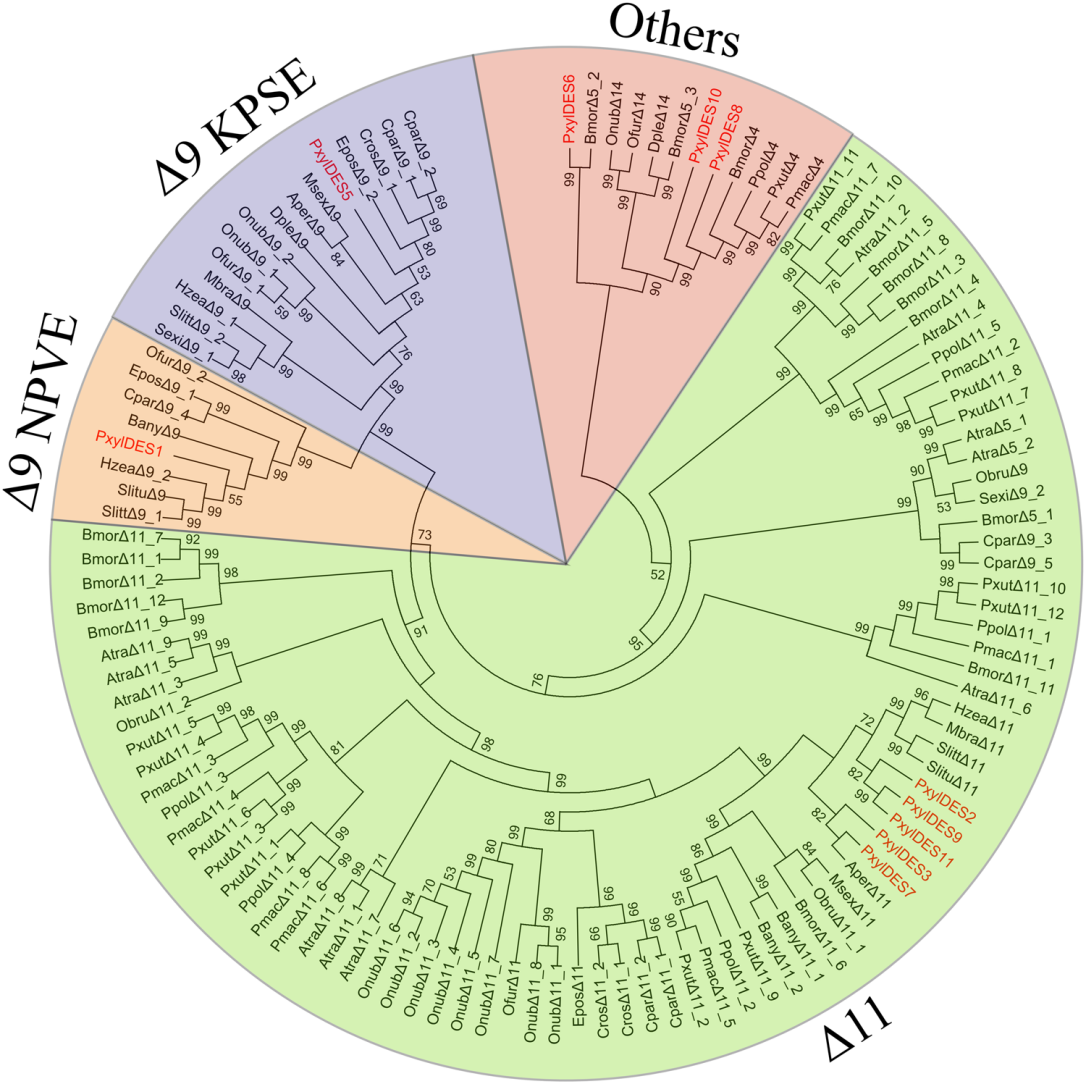
The phylogeny of DES genes. The neighbor-joining (NJ) consensus tree of DES genes was constructed using amino-acid sequences. Bootstrap values of NJ analyses are shown at the nodes as percent of total from 1000 bootstrap runs. The Δ9-DES with the KPSE motif, Δ9-DES with the NPVE motif, Δ11-DES and other DES gene groups were colored blue, yellow, green and red. Two transcripts in DBM PG were allocated in the Δ9-DES gene group, five in the Δ11-DES gene group and three in the other DES gene group.

The divergent regions of desaturase sequences were discarded, while the conservative regions of 290 aa in length were used to reconstruct a phylogenetic tree (Supplementary Table S2). The conservative regions of Des4 and Des12 were too short, so their sequences were abandoned. Des1 and Des5 were allocated together with Δ9-desaturases from other species and five desaturases of DBM were closely associated to the Δ11-desaturases (Fig. 3). The Δ9 signature motif of DES1 was NPVE, while the Δ9 signature motif of DES5 was unknown because of the incomplete ORF. The Δ11-DES signature motif of “xxxQ” was identified in DES3 and DES7, but not in other DBM Δ11-DES genes.

An oxygenated functional group (i.e., alcohol, aldehyde, or acetate ester) is a major class of sex pheromone. The key enzyme required to produce the oxygenated functional groups is FAR, which reduces fatty-acyl precursors to the corresponding alcohols, which can then be acetylated or oxidized to acetate esters or aldehydes, respectively^33^. We found amino acid sequences of 15 transcripts resembling FAR in the DBM PG transcriptome (Table 1). In this analysis, FAR1, FAR2, FAR3, FAR4 and FAR5 showed high expression levels within FPKM of more than 50. The FARs of DBM encode proteins with high amino acid sequence similarity to other Lepidoptera moths including *Bombyx mori*, *Helicoverpa assulta*, *Yponomeuta evonymellus* and *Spodoptera litura*.

ADH (EC 1.1.1.1) are a group of dehydrogenase enzymes that facilitate the interconversion between alcohols and aldehydes with the reduction of NAD^+^ to NADH^64^. ADH is a dimer protein and contains zinc at its catalytic site. Another aldehyde reductase group is ALR1 with EC number 1.1.1.2, which is monomeric NADPH-dependent oxidoreductases having wide substrate specificities for carbonyl compounds^65^. We found that the amino acid sequences encoded by five transcripts resemble ADH genes, while nine transcripts encode the proteins resemble ALR genes (Table 1). Moreover, most of them are highly expressed in PG transcriptome with more than 60 FPKM value.

Acetyltransferases (EC: 2.3.1) are probably the candidate genes for esterifying fatty alcohols into acetate esters. They belong to a huge family of acyl CoA-utilizing enzymes that transfer an acetyl group. However, the exact genes that are functionally involved in oxidization or acetylation have still not been cloned from any insect species^47^. Some key enzymes that belong to alcohol O-acetyltransferase family (EC: 2.3.1.84) in fungi^40^ and plants^66^ have been found to esterify fatty alcohol into acetate esters. We found two transcripts that encoded proteins homologous to acetyltransferases in DBM PG transcriptome (Table 1). The expression of ACT1 and ACT2 is high with more than 100 FPKM value. ACT1 is homologous to acetyl-CoA acetyltransferase from *Amyelois transitella*. ACT2 has the similar amino acid sequence to the acetyltransferase gene from *Spodoptera litura*. One acetyltransferase named ATF1 (EC 2.3.1.84) isolated from yeast was capable of acetylating fatty alcohols into acetates^40^, which provided some clue for insect sex pheromone biosynthesis. However, we did not identify any candidate gene that were homologues to ATF1 or to the genes belonging to the group of EC 2.3.1.84.

### Putative β-oxidation enzymes

In eukaryotes species, fatty acid molecules are broken down in the mitochondria to generate acetyl-CoA by β-oxidation catabolic process. β-oxidation may also play a vital role in regulating the ratio between sex pheromone compounds of different carbon lengths and breaking down of sex pheromones. Each cycle of β-oxidation liberates a two carbon unit of acetyl-CoA in a sequence of four reactions: oxidation of the fatty acid by acyl CoA oxidase (ACO: EC 1.3.3.6), hydration of the bond between C-2 and C-3 by enoyl CoA hydratase (ECH: EC 4.2.1.17), oxidation of L-β-hydroxyacyl CoA by NAD^+^ and 3-hydroxyacyl CoA dehydrogenase (HAD: EC 1.1.1.35), and the final step is the cleavage of β-ketoacyl CoA by the Coenzyme A and 3-ketoacyl-CoA thiolase (KAT: EC 2.3.1.16). We identified fourteen acyl CoA oxidase (ACO) genes, four enoyl CoA hydratase (ECH) genes, four 3-hydroxyacyl CoA dehydrogenase (HAD) genes and one 3-ketoacyl-CoA thiolase (KAT) gene in DBM PG transcriptome (Table 1), which indicate the role of β-oxidation in the breaking down of fatty acids and sex pheromone compounds. The genes involved in β-oxidation have been identified in some moth PG tissues^67^. β-oxidation is the catabolic process by which fatty acid molecules are broken down in the mitochondria in eukaryotes to generate acetyl-CoA, FADH2 and NADH. Moth species can also produce pheromone components by utilizing β-oxidation to shorten fatty acids chains to a limited length^68^.

### Putative pheromone and chemoreception carrier proteins

**Figure 4:**
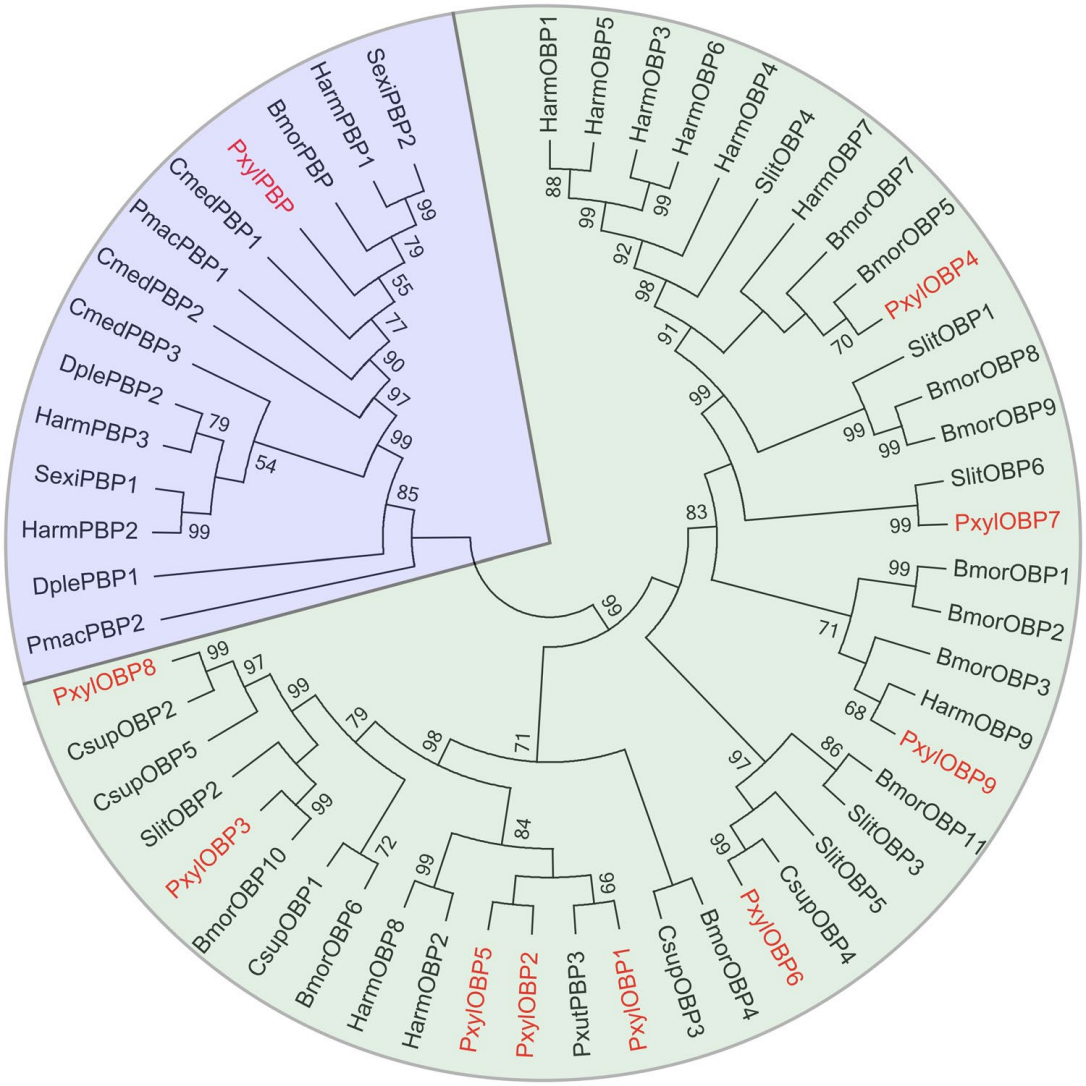
The phylogeny of OBP and PBP proteins. The neighbor-joining (NJ) consensus tree of OBP and PBP proteins as constructed using amino-acid sequences. Bootstrap values of NJ analyses are shown at the nodes as percent of total from 1000 bootstrap runs. The PBP and OBP gene groups were colored blue and green. One PBP and nine OBP genes that were expressed in DBM PG tissue were allocated to corresponding gene groups.

**Figure 5:**
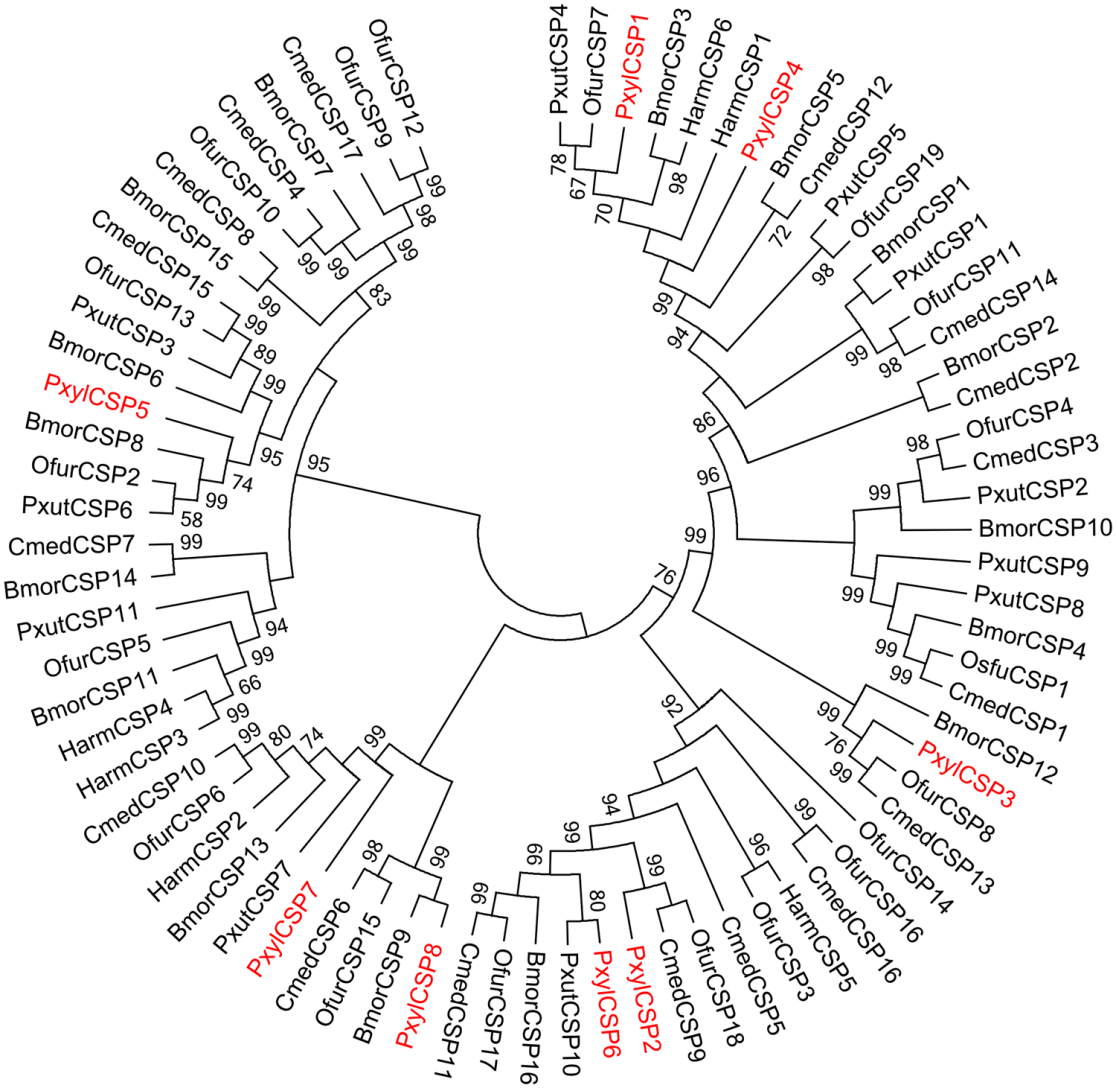
The phylogeny of CSP proteins is shown. The neighbor-joining (NJ) consensus tree of CSP proteins constructed using amino-acid sequences is described. Bootstrap values of NJ analyses are shown at the nodes as percent of total from 1000 bootstrap runs.

Odorant binding proteins (OBPs) are a major constituent of the aqueous proteins that might serve as solubilizers and carriers of the lipophilic odorants in insects. In the OBPs that are derived from moths, six cysteine residues are highly conserved with disulfide connectivity^69,70^. The OBP family genes that have been found to interact with sex pheromones are identified as pheromone-binding proteins (PBPs)^71^. Members of the OBP sub-family Minus-C do not contain all six conserved cysteine residues, while members in the sub-family Plus-C carry more than six conserved cysteine residues^72^. Another binding protein gene family that is involved in odorant sensory functions are known as chemosensory proteins (CSP), which contain only four conserved cysteines^73^. These binding proteins are not only expressed in the sensilla of the antennae, but can also be identified in the sensilla of the ovipositor^74^. The presence of chemosensilla on the ovipositor indicates the chemoreception function of odors^75^, or a feedback loop in the moth’s PG to control the biosynthesis pathway and release of sex pheromones^76^. OBP and CSP have been demonstrated in the function of binding and transportation of hydrophobic volatile molecules, including sex pheromones, plant and environment volatiles. A total of 8 CSP, 9 OBP, as well as 1 PBP transcripts were identified, which are major constituent of the aqueous proteins that might serve as solubilizers and transporters of fatty acids and sex pheromone compounds (Table 2). The PBP candidate of DBM grouped together with other PBP genes, while OBP candidates were allocated with OBP genes of other species (Fig. 4). The phylogenetic analysis of CSP genes between different species shows the CSP candidates of DBM are homologous to other species (Fig. 5).

**Table 2:** The BlastX match of transcripts involved in chemoreception genes.

Gene_id	Gene name	Gene Length	ORF	Accession Number	Putative identification	Species	Score(bits)	Expect value	Signal peptide
CSP									
c43624_g1	CSP1	558	462	ALJ30213.1	putative chemosensory protein CSP2	*Spodoptera litura*	200	5E-64	N
c45873_g1	CSP2	758	564	AND82447.1	chemosensory protein 5	*Athetis dissimilis*	159	3E-48	1–43
c46712_g1	CSP3	699	540	EHJ76401.1	chemosensory protein CSP1	*Danaus plexippus*	159	2E-46	1–22
c48825_g1	CSP4	1196	390	ABM67689.1	chemosensory protein CSP2	*Spodoptera exigua*	179	7E-56	1–21
c42390_g1	CSP5	577	420	AGI37363.1	chemosensory protein 2	*Cnaphalocrocis medinalis*	144	6E-42	1–31
c49085_g1	CSP6	747	507	BAF91720.1	chemosensory protein	*Papilio xuthus*	184	3E-58	1–38
c46947_g1	CSP7	524	438	AII01029.1	chemosensory protein	*Dendrolimus kikuchii*	162	3E-49	N
c44879_g1	CSP8	628	507	ALJ30215.1	putative chemosensory protein CSP4	*Spodoptera litura*	234	7E-75	1–27
OBP							1-23		
c42398_g1	OBP1	509	441	AFD34173.1	odorant binding protein 5	*Argyresthia conjugella*	246	7E-82	1–27
c46180_g1	OBP2	652	453	AFD34182.1	odorant binding protein 6	*Argyresthia conjugella*	189	2E-59	1–20
c46457_g1	OBP3	552	429	AII00979.1	odorant binding protein	*Dendrolimus houi*	160	4E-48	N
c48199_g1	OBP4	661	549	AFD34177.1	odorant binding protein 1	*Argyresthia conjugella*	131	4E-36	1–24
c49505_g1	OBP5	670	420	ALC79591.1	odorant binding protein 11	*Grapholita molesta*	203	2E-65	1–20
c49705_g1	OBP6	678	546	AGK24580.1	odorant-binding protein 4	*Chilo suppressalis*	207	7E-66	N/C
c53540_g2	OBP7	1168	648	XP_014371749.1	general odorant-binding protein 70	*Papilio machaon*	350	2E-121	1–31
c47978_g1	OBP8	725	447	XP_013147646.1	general odorant-binding protein 56a-like	*Papilio polytes*	86.3	4E-19	N
c55649_g1	OBP9	918	315	XP_011557111.1	general odorant-binding protein 72-like isoform X1	*Plutella xylostella*	41.6	0.023	N
PBP									
c48105_g1	PBP	742	540	AFD34179.1	pheromone binding protein 3	*Argyresthia conjugella*	244	2E-80	1–18

N: no signal peptide was identified.

### Tissue expression profiles

**Figure 6:**
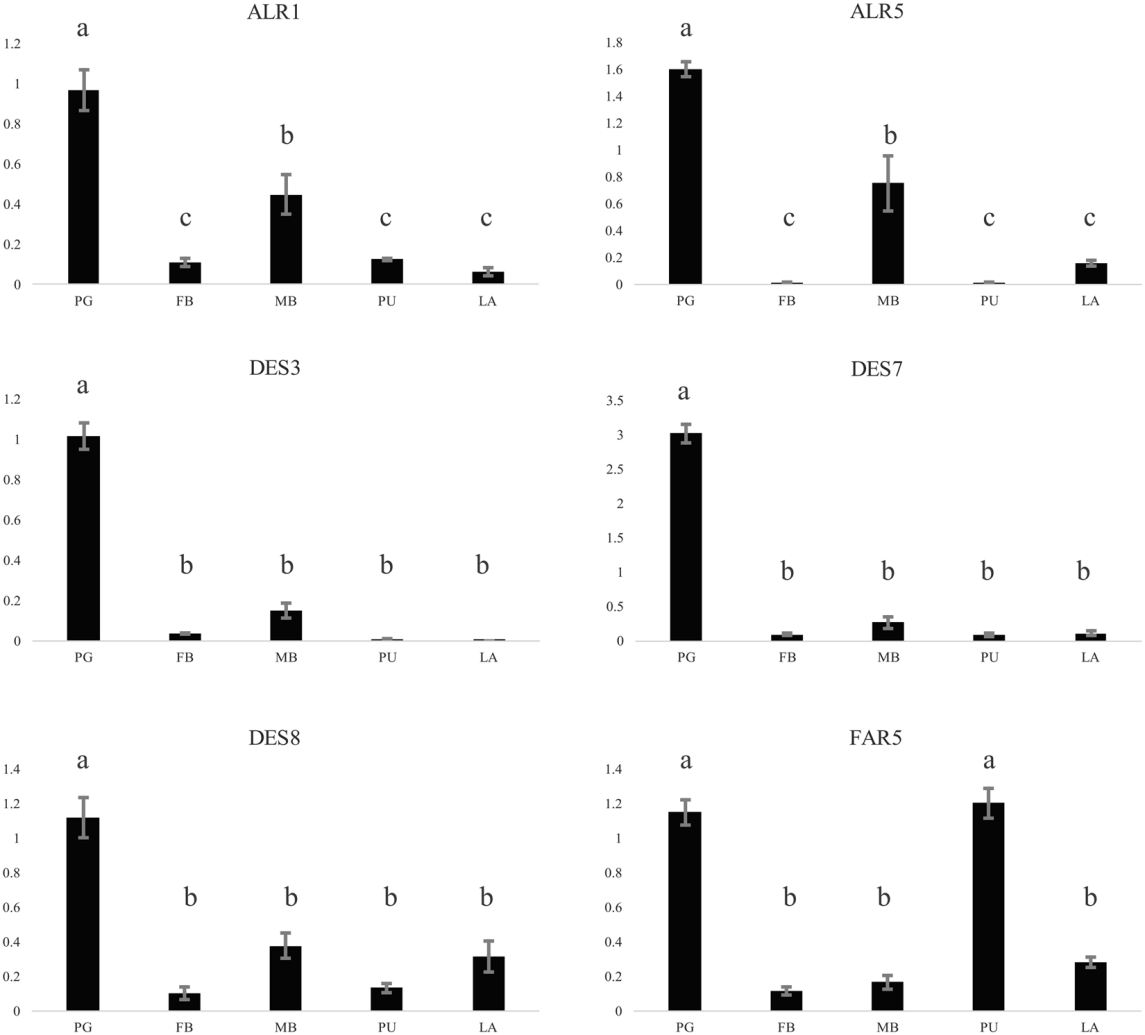
Relative expression levels of pheromone biosynthesis genes as determined by qPCR. The gene expression levels in PG tissue as compared the female moth body without PG tissue (FB), male moth body (MB), pupa (PU) and larva (LA) are shown. The standard error is represented by the error bar, and the different letters above each bar represent significant differences (p < 0.05). Abbreviation: ALR: aldo-keto reductase, DES: desaturase, FAR: fatty acyl-CoA reductase. Note: DES3 and DES7 were identified as Δ11 reductase while DES8 had closed relationship with Δ4 reductase.

**Figure 7:**
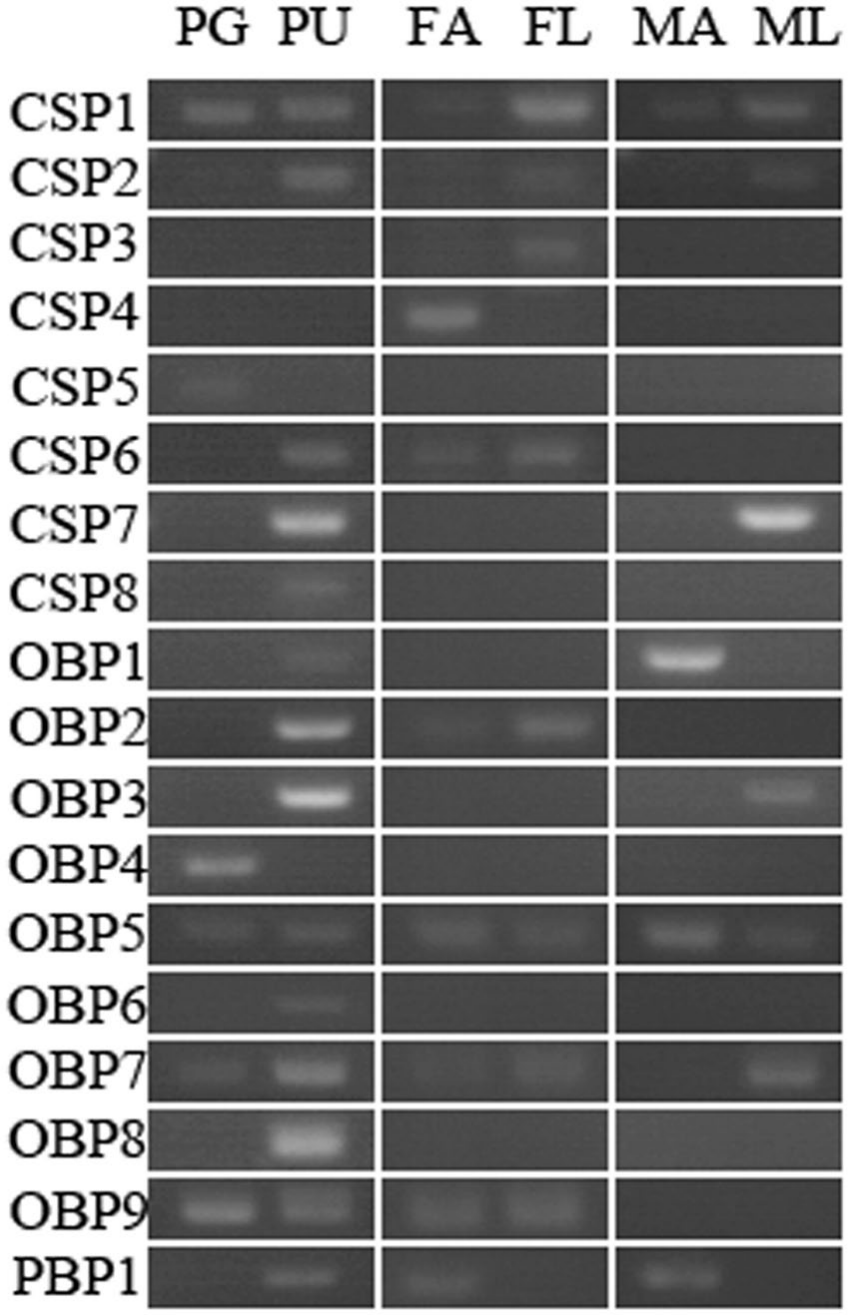
Tissue- and sex- specific expression analysis of pheromone and chemoreception carrier protein genes by using reverse transcription PCR. Abbreviation: PG: pheromone gland, PU: pupa, FA: female antenna, FL: female leg, MA: male antenna, ML: male leg.

After compared the expression levels of the gene involved in the sex pheromone biosynthesis by qRT-PCR between pheromone glands (PG), female body without PG (FB), male body (MB), pupa tissue (PU) and larva tissue (LA), five genes were found to be expressed in PG tissue at high levels compared to other tissues, including two ALR genes and three DES genes (Fig. 6). FAR5 genes were found to be highly expressed both in PG tissue and pupa tissues. The gene expression profiles of many putative genes identified in pheromone gland do not show any specificity, which is probably due to the fact these enzymes are involved in multiple basic biological pathways. However, some enzymes that are involved in double bond desaturation and functional group transfer are specifically and highly expressed in PG tissues as compared to tissues of larva, pupa, and the male and female abdomen, which indicated that the DES, FAR and ALR genes might be involved in sex pheromone biosynthesis in PG tissue^57^. We also checked the expression of CSP, OBP and PBP genes in PG, pupa, antenna and legs by semi-quantitative PCR analysis (Fig. 7). Some genes expressed extensively in a certain tissues, like CSP1, CSP2, CSP6, OBP5, OBP7 and OBP9. One PBP gene was found to be expressed in pupa tissue, female and male antenna. But PBP gene expression was not detected in PG tissue, which was probably due to its low expression level (FPKM: 13.87). OBP4 and CSP5 genes were extensively expressed in PG tissue, though the band of CSP5 was weak. OBPs and CSPs are usually expressed in the sensilla of the antenna, leg and ovipositor that were not expressed specifically in PG tissues, as shown by over half of the qPCR analyses conduced in DBM PG and other tissues. Furthermore, OBP4 was expressed with a high FPKM value and was specifically expressed in the PG tissue, which indicated its vital role in odorant sensation or chemical molecular transport.

## Methods

### Moth collection and rearing

The DBM larvae were originally collected from a broccoli field in Guangzhou, Guangdong province, China (N22°56′; E113°26′). The collected larvae were reared in laboratory with broccoli plants continually under the conditions of 25 °C, 60–70% relative humidity and a 16:8 light: dark photoperiod. Last instar larvae were separated by the undertint spot represented the testis. Fifty pheromone glands (PGs) from third day eclosion virgins were dissected for cDNA library construction. In addition, the tissues including larvae, pupae, PGs, male and female abdomens were collected and froze in liquid nitrogen until RNA extraction.

### cDNA library construction and Illumina sequencing

Total RNA was extracted using TRIzol regent according to the manufacturer’s protocol. RNA degradation and contamination was monitored on 1% agarose gels electrophoresis. The NanoPhotometer^®^ spectrophotometer was used to check RNA purity and concentration. RNA integrity was assessed using the RNA Nano 6000 Assay Kit of the Agilent Bioanalyzer 2100 system. A total amount of 1.5 µg RNA was used for preparing sequencing library generated by NEBNext® Ultra™ RNA Library Prep Kit. Briefly, mRNA was purified from total RNA using poly-T oligo-attached magnetic beads. Fragmentation was carried out using divalent cations under elevated temperature in NEBNext First Strand Synthesis Reaction Buffer (5×).

First strand cDNA was synthesized using random hexamer primer and M-MuL V Reverse Transcriptase (RNase H^−^). Second strand cDNA synthesis was subsequently performed using DNA Polymerase I and RNase H. After adenylation of 3′ ends of DNA fragments, NEBNext Adaptor with hairpin loop structure were ligated to prepare for hybridization. PCR was performed with Universal PCR primers and Index (X) Primer. After cluster generation on a cBot Cluster Generation System, the library preparations were sequenced on an Illumina Hiseq platform and paired-end reads were generated. The raw data were deposited in the NCBI Short Read Archive (SRA) database with BioProject accession number: SRP076084. Raw reads of fastq format were firstly processed by removing reads containing adapter, reads containing ploy-N and low quality reads from raw data to obtain the clean reads. More than 56.6 million clean reads were obtained with about 8.5 G base pairs.

### Transcriptome reconstruction

We attempted to map the clean reads to the genomic sequences of DBM that were obtained from an open access NCBI genomic database^48^. However, the overall alignment rate of the mapping results output by HISAT2^77^ was low (56.49%). To avoid data residuals, the program Trinity^78,79^ was used to reconstruct the transcriptome with parameters of the min_kmer_cov set to a value of two, and all other parameters set to the default value and abandoning all sequences that were shorter than 200 bp.

### Bioinformatic analysis

Functional annotations of transcripts were conducted towards the NR, NT, and the Swiss-Prot with an e-value less than 1 × 10^−5^ and KOG with an e-value that was less than 1 × 10^−3^ that was based on sequence similarity using the NCBI BlastX software suite. Based on NR annotation, Blast2GO program was used to get GO annotation and WEGO software was used for GO functional classification. Clean data were mapped back onto the assembled transcriptome by using Bowtie 2 and a read count for each gene that was obtained from the mapping results. Transcriptomic expression abundance was estimated by the RSEM (RNA-Seq by Expectation Maximization) method^80^. The ORFs (open reading frame) of the putative fatty acid biosynthesis genes were calculated by the ORF Finder online method (http://www.ncbi.nlm.nih.gov/gorf/orfig.cgi). The amino acid sequences of putative fatty acid biosynthesis genes were translated according to results obtained from the ORF Finder based on standard genetic codes.

### Phylogenetic relationship calculation

Sequences used for phylogenetic reconstructions were retrieved from the GenBank database (Supplementary Tables S1–S4). Multiple sequences were aligned by ClustalW^53^ module in MEGA7 software^54^. The raw output of the multiple sequence alignments were refined to minimize insertion/deletion events^81^. Optimum phylogenetic model was calculated by MEGA7. The evolutionary relationship was inferred using the Neighbor-Joining method performed by MEGA7 with optimum phylogenetic model. Branch supports were surveyed by bootstrapping 1000 times.

### RNA isolation and quantitative real time PCR

Total RNA from the tissues of larvae, pupae, PGs, and male and female abdomens was isolated using TRIzol reagent according to the manufacturer’s instructions. Single-stranded cDNA was synthesized using the TransScript One-Step gDNA Removal and cDNA Synthesis SuperMix (Transgen) kit. Specific primer pairs for qRT-PCR analysis were designed with Oligo 7 (Supplementary Table S5). The primer for reference genes were designed according the sequence of elongation factor 1 gene (EF1) (accession number EF417849) and ribosomal protein L32 gene (RPL32) (accession number AB180441)^82^ for normalizing expression of the target gene and correcting for sample-to-sample variation.

Quantitative RT-PCR was performed with TransStart Top Green qPCR SuperMix (Transgen) according to the manufacturer’s instructions. The cycling conditions were 94 °C for 30 s followed by 40 cycles of 94 °C for 5 s and 60 °C for 30 s. Then, the PCR products were heated to 95 °C for 1 min, cooled to 55 °C for 30 s and heated to 95 °C for 30 sec to measure the dissociation curves. Blank qTR-PCR, which comprised an added template without the primer was included in each experiment and served as the negative control. The genes involved in pheromone biosynthesis were compared in different tissues. Then each genes expressed at high levels in PG tissue was carried out in three technical replicates and three biological replicates of qRT-PCR survey to check reproducibility of the assays. Relative quantification was performed using the comparative 2-ΔΔCt method^83^. Data (mean 6 SE) from various samples were determined by one-way nested analysis of variance (ANOVA) followed by a least significant difference test (LSD) for mean comparisons. RT-PCR was performed with EasyTaq DNA Polymerase (Transgen) according to the manufacturer’s instructions. The cycling conditions were 94 °C for 2 m followed by 35 cycles of 94 °C for 30 s, 55 °C for 30 s and 70 °C for 1 m. 10 μL of each PCR product was examined on a 2% agarose gel after 30 minutes of standard electrophoresis at 130 V and 15 min of staining with standard application of GelStain (Transgen).

## Electronic supplementary material

Supplementary information

## References

[1] Zalucki MP (2012). Estimating the economic cost of one of the world’s major insect pests, *Plutella xylostella* (Lepidoptera: Plutellidae): just how long is a piece of string?. J Econ Entomol.

[2] Food and Agriculture Organization of the United Nations. *FAOSTAT* http://faostat3.fao.org/browse/Q/QC/E (2016).

[3] Attique MNR, Khaliq A, Sayyed AH (2006). Could resistance to insecticides in *Plutella xylostella* (Lep., Plutellidae) be overcome by insecticide mixtures?. J Appl Entomol.

[4] Kirsch K, Schmutterer H (1988). Low efficacy of a *Bacillus thuringiensis* (Berl.) formulation in controlling the diamondback moth, *Plutella xylostella* (L.), in the Philippines. J Appl Entomol.

[5] Furlong MJ, Wright DJ, Dosdall LM (2013). Diamondback moth ecology and management: problems, progress, and prospects. Annu Rev Entomol.

[6] Talekar NS, Shelton AM (1993). Biology, ecology, and management of the diamondback moth. Annu Rev Entomol.

[7] Li Z, Feng X, Liu SS, You M, Furlong MJ (2016). Biology, ecology, and management of the diamondback moth in China. Annu Rev Entomol.

[8] Witzgall P, Kirsch P, Cork A (2010). Sex pheromones and their impact on pest management. J Chem Ecol.

[9] McNeil JN (1991). Behavioral ecology of pheromone-mediated communication in moths and its importance in the use of pheromone traps. Annu Rev Entomol.

[10] Dai JQ, Deng JY, Du JW (2008). Development of bisexual attractants for diamondback moth, *Plutella xylostella* (Lepidoptera: Plutellidae) based on sex pheromone and host volatiles. Appl Entomol Zool.

[11] Koshihara T, Yamada H, Tamaki Y, Ando A (1978). Field attractiveness of the synthetic sex-pheromone of the diamondback moth, *Plutella xylostella* (L.). Appl Entomol Zool.

[12] Tamaki Y (1977). (*Z*)-11-hexadecenal and (*Z*)-11-hexadecenyl acetate: sex-pheromone components of the diamondback moth (Lepidoptera: Plutellidae). Appl Entomol Zool.

[13] Chou Y, Lin Y, Hsu C (1977). Sex pheromone of the diamondback moth (Lepidoptera: Plutellidae). Bull Inst Zool Acad Sin.

[14] Ando T (1979). Electroantennogram activities of sex pheromone analogues and their synergistic effect on field attraction in the diamondback moth. Appl Entomol Zool.

[15] Chisholm MD, Steck WF, Underhill EW, Palaniswamy P (1983). Field trapping of diamondback moth *Plutella xylostella* using an improved four-component sex attractant blend. J Chem Ecol.

[16] Koshihara T, Yamada H (1980). Attractant activity of the female sex-pheromone of diamondback moth, *Plutella xylostella* (L) and analogue. Jpn J Appl Entomol Zool.

[17] Yang CY, Lee S, Choi KS, Jeon HY, Boo KS (2007). Sex pheromone production and response in Korean populations of the diamondback moth. Plutella xylostella. Entomol Exp Appl.

[18] Maa C, Lin Y, Chow Y (1984). Population variations in male response to female sex pheromone of *Plutella xylostella* (L.) in northern Taiwan. Plant Prot. Bull.

[19] Zilahi‐Balogh GMG (1995). Regional differences in pheromone responses of diamondback moth in Indonesia. Int J Pest Manage.

[20] Ando, T., Inomata, S. & Yamamoto, M. In *Topics in Current Chemistry* Vol. 239 (ed S. Schulz) 51-96 (Springer Berlin Heidelberg, 2004). 10.1007/b95449 22160231

[21] Blomquist, G. J. & Vogt, R. G. *Insect Pheromone Biochemistry and Molecular Biology*. (Academic press, 2003).

[22] Millar JG (2000). Polyene hydrocarbons and epoxides: A second major class of lepidopteran sex attractant pheromones. Annu Rev Entomol.

[23] Matsumoto S (2010). Molecular mechanisms underlying sex pheromone production in moths. Biosci, Biotechnol, Biochem.

[24] Volpe JJA, Vagelos PR (1973). Saturated fatty acid biosynthesis and its regulation. Annu Rev Biochem.

[25] Foster SP, Roelofs WL (1996). Sex pheromone biosynthesis in the tortricid moth, *Ctenopseustis herana* (Felder & Rogenhofer). Arch Insect Biochem Physiol.

[26] Wang HL, Lienard MA, Zhao CH, Wang CZ, Lofstedt C (2010). Neofunctionalization in an ancestral insect desaturase lineage led to rare Δ6 pheromone signals in the Chinese tussah silkworm. Insect Biochem Mol Biol.

[27] Lofstedt C, Bengtsson M (1988). Sex pheromone biosynthesis of (*E*,*E*)-8,10-dodecadienol in codling moth *Cydia pomonella* involves *E*9 desaturation. J Chem Ecol.

[28] Foster SP, Roelofs WL (1988). Sex pheromone biosynthesis in the leafroller moth *Planotortix excessana* by Δ10 desaturation. Arch Insect Biochem Physiol.

[29] Bjostad LB, Roelofs WL (1981). Sex pheromone biosynthesis from radiolabeled fatty acids in the redbanded leafroller moth. J Biol Chem.

[30] Moto K (2004). Involvement of a bifunctional fatty-acyl desaturase in the biosynthesis of the silkmoth, *Bombyx mori*, sex pheromone. Proc Natl Acad Sci USA.

[31] Zhao C, Löfstedt C, Wang X (1990). Sex pheromone biosynthesis in the Asian corn borer *Ostrinia furnacalis* (II): Biosynthesis of (*E*)- and (*Z*)-12-tetradecenyl acetate involves Δ14 desaturation. Arch Insect Biochem Physiol.

[32] Houten SM, Wanders RJA (2010). A general introduction to the biochemistry of mitochondrial fatty acid β-oxidation. J Inherited Metab Dis.

[33] Moto K (2003). Pheromone gland-specific fatty-acyl reductase of the silkmoth. Bombyx mori. Proc Natl Acad Sci USA.

[34] Lienard MA, Hagstrom AK, Lassance JM, Lofstedt C (2010). Evolution of multicomponent pheromone signals in small ermine moths involves a single fatty-acyl reductase gene. Proc Natl Acad Sci USA.

[35] Lassance JM (2013). Functional consequences of sequence variation in the pheromone biosynthetic gene pgFAR for Ostrinia moths. Proc Natl Acad Sci USA.

[36] Hagstrom AK, Lienard MA, Groot AT, Hedenstrom E, Lofstedt C (2012). Semi-selective fatty acyl reductases from four heliothine moths influence the specific pheromone composition. PLoS One.

[37] Teal PE, Tumlinson JH (1988). Properties of cuticular oxidases used for sex pheromone biosynthesis by *Heliothis zea*. J Chem Ecol.

[38] Teal PEA, Tumlinson JH (1987). The role of alcohols in pheromone biosynthesis by two noctuid moths that use acetate pheromone components. Arch Insect Biochem Physiol.

[39] Winberg J, Thatcher DR, McKinley-McKee JS (1982). Alcohol dehydrogenase from the fruitfly *Drosophila melanogaster* substrate specificity of the alleloenzymes AdhS and AdhUF. Biochim Biophys Acta.

[40] Ding BJ (2016). The yeast ATF1 acetyltransferase efficiently acetylates insect pheromone alcohols: implications for the biological production of moth pheromones. Lipids.

[41] Morse D, Meighen E (1987). Biosynthesis of the acetate ester precursor of the spruce budworm sex pheromone by an acetyl CoA: fatty alcohol acetyltransferase. Insect Biochem.

[42] Gimeno RE (2007). Fatty acid transport proteins. Curr Opin Lipidol.

[43] Rasmussen JT, Faergeman NJ, Kristiansen K, Knudsen J (1994). Acyl-CoA-binding protein (ACBP) can mediate intermembrane acyl-CoA transport and donate acyl-CoA for *β*-oxidation and glycerolipid synthesis. Biochem J.

[44] Zhang YN, Xia YH, Zhu JY, Li SY, Dong SL (2014). Putative pathway of sex pheromone biosynthesis and degradation by expression patterns of genes identified from female pheromone gland and adult antenna of *Sesamia inferens* (Walker). J Chem Ecol.

[45] Xia YH, Zhang YN, Hou XQ, Li F, Dong SL (2015). Large number of putative chemoreception and pheromone biosynthesis genes revealed by analyzing transcriptome from ovipositor-pheromone glands of *Chilo suppressalis*. Sci Rep.

[46] Li ZQ (2015). Transcriptome comparison of the sex pheromone glands from two sibling *Helicoverpa* species with opposite sex pheromone components. Sci Rep.

[47] Ding BJ, Lofstedt C (2015). Analysis of the *Agrotis segetum* pheromone gland transcriptome in the light of sex pheromone biosynthesis. BMC Genomics.

[48] You MS (2013). A heterozygous moth genome provides insights into herbivory and detoxification. Nat Genet.

[49] Raina AK (1989). Identification of a neuropeptide hormone that regulates sex pheromone production in female moths. Science.

[50] Choi MY, Fuerst EJ, Rafaeli A, Jurenka R (2003). Identification of a G protein-coupled receptor for pheromone biosynthesis activating neuropeptide from pheromone glands of the moth *Helicoverpa zea*. Proc Natl Acad Sci USA.

[51] Hull JJ (2004). Cloning and characterization of the pheromone biosynthesis activating neuropeptide receptor from the Silkmoth, *Bombyx mori*: Significance of the carboxyl terminus in receptor internalization. J Biol Chem.

[52] Lee DW (2011). RNA interference of pheromone biosynthesis-activating neuropeptide receptor suppresses mating behavior by inhibiting sex pheromone production in *Plutella xylostella* (L.). Insect Biochem Mol Biol.

[53] Thompson JD, Higgins DG, Gibson TJ (1994). CLUSTAL W: improving the sensitivity of progressive multiple sequence alignment through sequence weighting, position-specific gap penalties and weight matrix choice. Nucleic Acids Res.

[54] Kumar S, Stecher G, Tamura K (2016). MEGA7: Molecular evolutionary genetics analysis version 7.0 for bigger datasets. Mol Biol Evol.

[55] Saitou N, Nei M (1987). The neighbor-joining method: a new method for reconstructing phylogenetic trees. Mol Biol Evol.

[56] Jones DT, Taylor WR, Thornton JM (1992). The rapid generation of mutation data matrices from protein sequences. Comput Appl Biosci.

[57] Tillman JA, Seybold SJ, Jurenka RA, Blomquist GJ (1999). Insect pheromones—an overview of biosynthesis and endocrine regulation. Insect Biochem Mol Biol.

[58] Strandh M, Johansson T, Ahrén D, Löfstedt C (2008). Transcriptional analysis of the pheromone gland of the turnip moth, *Agrotis segetum* (Noctuidae), reveals candidate genes involved in pheromone production. Insect Mol Biol.

[59] Jurenka R, Rafaeli A (2011). Regulatory role of PBAN in sex pheromone biosynthesis of heliothine moths. Front Endocrinol.

[60] Smith S (1994). The animal fatty acid synthase: one gene, one polypeptide, seven enzymes. FASEB J.

[61] Knipple DC (1998). Cloning and functional expression of a cDNA encoding a pheromone gland-specific acyl-CoA Δ11-desaturase of the cabbage looper moth. Trichoplusia ni. Proc Natl Acad Sci USA.

[62] Bjostad LB, Roelofs WL (1983). Sex pheromone biosynthesis in *Trichoplusia ni*: Key steps involve delta-11 desaturation and chain-shortening. Science.

[63] Knipple DC, Rosenfield CL, Nielsen R, You KM, Jeong SE (2002). Evolution of the integral membrane desaturase gene family in moths and flies. Genetics.

[64] W Sofer A, Martin PF (1987). Analysis of alcohol dehydrogenase gene expression in *Drosophila*. Annu Rev Genet.

[65] Bohren KM, Bullock B, Wermuth B, Gabbay KH (1989). The aldo-keto reductase superfamily. cDNAs and deduced amino acid sequences of human aldehyde and aldose reductases. J Biol Chem.

[66] Shalit M (2001). Acetyl-coa: alcohol acetyltransferase activity and aroma formation in ripening melon fruits. J Agric Food Chem.

[67] Vogel H, Heidel AJ, Heckel DG, Groot AT (2010). Transcriptome analysis of the sex pheromone gland of the noctuid moth *Heliothis virescens*. BMC Genomics.

[68] Roelofs WL, Wolf WA (1988). Pheromone biosynthesis in lepidoptera. J Chem Ecol.

[69] Scaloni A, Monti M, Angeli S, Pelosi P (1999). Structural analysis and disulfide-bridge pairing of two odorant-binding proteins from *Bombyx mori*. Biochem Biophys Res Commun.

[70] Leal WS, Nikonova L, Peng G (1999). Disulfide structure of the pheromone binding protein from the silkworm moth. Bombyx mori. FEBS Lett.

[71] Vogt RG, Riddiford LM (1981). Pheromone binding and inactivation by moth antennae. Nature.

[72] Hekmat-Scafe DS, Scafe CR, McKinney AJ, Tanouye MA (2002). Genome-wide analysis of the odorant-binding protein gene family in *Drosophila melanogaster*. Genome Res.

[73] McKenna MP, Hekmat-Scafe DS, Gaines P, Carlson JR (1994). Putative *Drosophila* pheromone-binding proteins expressed in a subregion of the olfactory system. J Biol Chem.

[74] Zhang YN (2015). Identification and expression profiles of sex pheromone biosynthesis and transport related genes in *Spodoptera litura*. PLoS One.

[75] Faucheux MJ (1988). Multiporous sensilla on the ovipositor of *Monopis crocicapitella* Clem. (Lepidoptera: Tineidae). Int J Insect Morphol Embryol.

[76] Dani FR (2011). Odorant-binding proteins and chemosensory proteins in pheromone detection and release in the silkmoth *Bombyx mori*. Chem Senses.

[77] Kim D, Langmead B, Salzberg SL (2015). HISAT: a fast spliced aligner with low memory requirements. Nat Methods.

[78] MG G (2011). Full-length transcriptome assembly from RNA-Seq data without a reference genome. Nat Biotechnol.

[79] Haas BJ (2013). *De novo* transcript sequence reconstruction from RNA-seq using the Trinity platform for reference generation and analysis. Nat Protoc.

[80] Li B, Dewey CN (2011). RSEM: accurate transcript quantification from RNA-Seq data with or without a reference genome. BMC Bioinformatics.

[81] Baldauf SL (2003). Phylogeny for the faint of heart: a tutorial. Trends Genet.

[82] Gao X (2016). Identification and characterization of the gene *CYP340W1* from *Plutella xylostella* and its possible involvement in resistance to abamectin. Int J Mol Sci.

[83] Livak KJ, Schmittgen TD (2001). Analysis of relative gene expression data using real-time quantitative PCR and the 2^−ΔΔCT^ Method. Methods.

