# Supplementary material for: Identification of the pheromone biosynthesis genes from the sex pheromone gland transcriptome of the diamondback moth, Plutella xylostella
Source: Sci Rep. 2017 Nov 24;7:16255. doi: 10.1038/s41598-017-16518-8 (PMC5701256; doi:10.1038/s41598-017-16518-8)
Supplement: Supplementary file 1 — Supplementary information [file 41598_2017_16518_MOESM1_ESM.pdf]

Identification of the pheromone biosynthesis genes from the sex pheromone gland transcriptome of the diamond back moth, *Plutella xylostella*

Da-Song Chen[1], Jian-Qing Dai[1] & Shi-Chou Han[1]

[1]Guangdong Key Laboratory of Animal Conservation and Resource Utilization, Guangdong Public Laboratory of Wild Animal Conservation and Utilization, Guangdong Institute of Applied Biological Resources, Guangzhou, China.

Correspondence and requests for materials should be addressed to J.Q.D. (jqdai@giabr.gd.cn) or S.C.H. (hansc@giabr.gd.cn)

Supplementary information

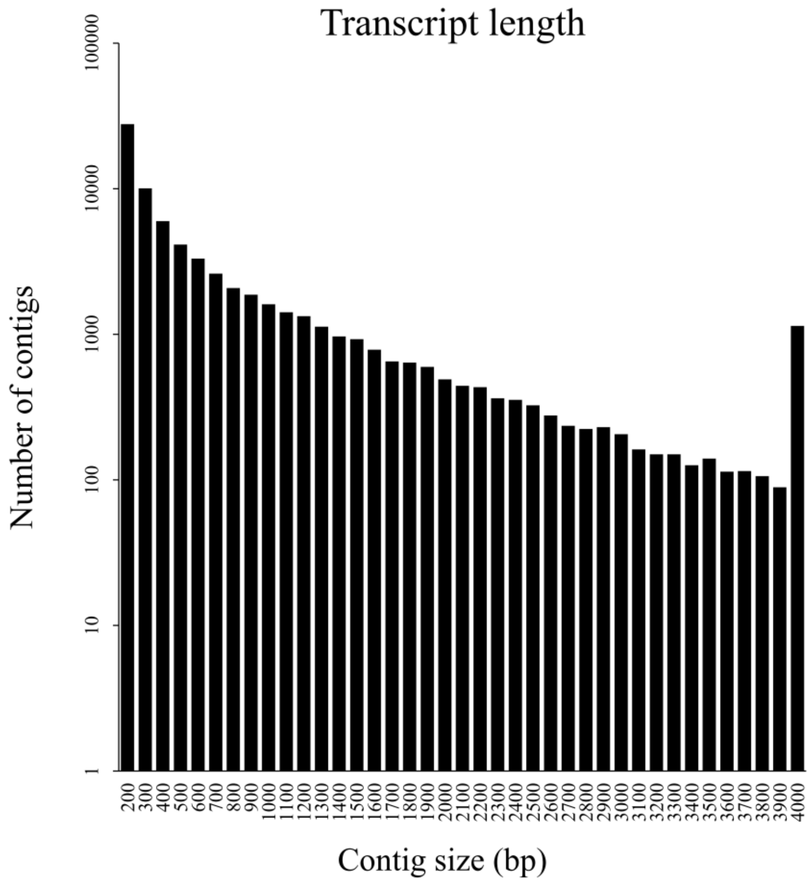

Supplementary Figure S1. Length distribution of contigs. The consensus sequence lengths ranged from 200 bp to > 4,000 bp. Each column indicates the number of contigs of each length range. The most abundant unigenes were 200 bp (27776) and the least abundant unigenes were 4000 bp (89). Contigs > 4000 bp (1144) were grouped together.

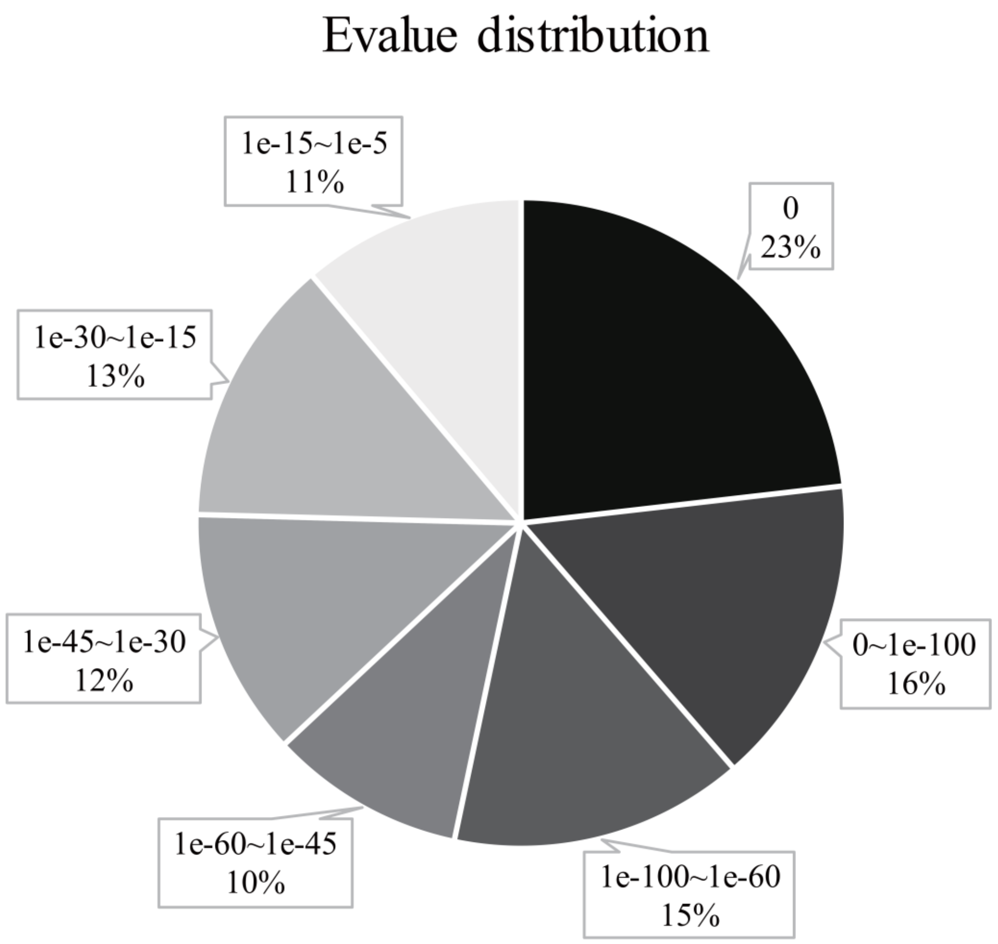

Supplementary Figure S2. E-value distribution of the BlastX results. Distribution of matched sequences by BlastX search against the NCBI-NR database. The percentage values demonstrated the relative proportion of the corresponding E-value interval. Significant matched sequences were defined as having an e-value $\leqslant 10^{-5}$.

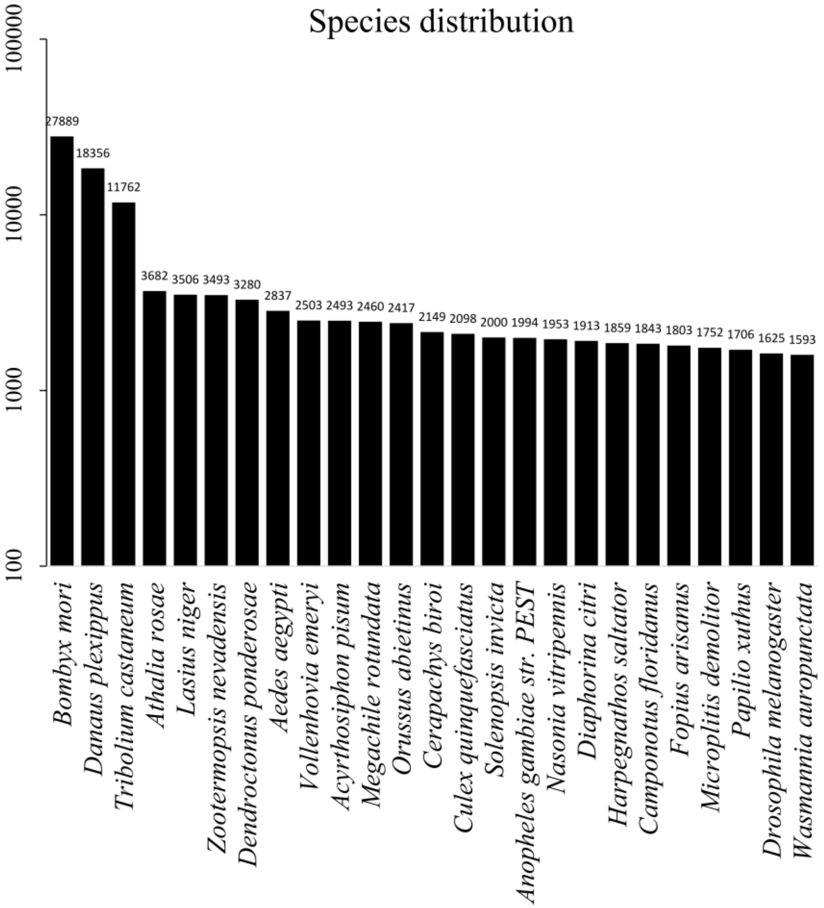

Supplementary Figure S3. Species distribution of the BlastX results. The significant hits with an e-value $\leqslant 10^{-5}$ for each query were grouped according to the species of the BlastX hits. The number of contigs that had significant homology is indicated on top of the columns.

Supplementary Table S1. Sequence details used in the alignment and phylogenetic analysis of PBANR genes.

Species name	Accession number	Sequence definition	Abbreviation	Amino acid sequence
Agrotis segetum	AID66638.1	pheromone biosynthesis activating neuropeptide receptor	AgsePBANR	-MTLPAPPSIDEYEDPF--VMNTTNVT-<br>SHPAAYDEQYALDLVVPLTVTYGIIFVAGILGNTSTCVVIARNRSMHTATNFYLFSLAISDLILLVCGLPFEVHRLWNPDTYPLG<br>EAHCIAIGLASETSANATVLTITAFTVERYIAICRPFMSHTMSKLSRAVRFIIAIWVVALCTAVPQAMQFGIVSYVDNGVNVSAC<br>TVKGVGVHQVFVISSFVFFVVPMSMISVLYALIGVKLRTSRVLHPVKKLSVDSNERAS-<br>GQMQYRNGASQRRVIRMLVAVALSFFICWAPFHVQRLLAIYGKSLEHPSDTFYLVYIVLTFLSGVLYFLSTAINPFLYNIMSNKF<br>RNAFKVTLATWCGRGGPRTGRTYSALLASQRLRAGGPGACASVRGTRRLRRLSTATTQLYDAPPRAQVSATTGAIYQPLTSP<br>QAETG--IGPELGDYVSTTHPTLSTPLEASQTP---------------------------
Bombyx mori	AEX15646.1	pheromone biosynthesis activating neuropeptide receptor isoform A	BomoPBANR_A	-MMADETVNMEM-LENN--LLNVTNVT-<br>DQSSAYSESYPLHLLVPLSVTYAVIFIVGILGNTSTCVVIARNRSMHTATNFYLFSLAISDIILLVCGLPLELYRLWNPFTYPLGEA<br>QCITIGLASETSANATVLTITAFTMERYIAICRPFMSHTMSKLSRAVRFIIAIWVFALCTAVPQAMQFGIVSYVENGQSMSACTV<br>KGPGVHQVFVISSFVFFVVPMSVISVLYALIGLKLRTSRILHPVKKLSLDSNERPG-<br>AHTPYRNGSSQRRVIRMLVAVALSFFICWAPFHVQRLLAIYGKSLEHPSDTFYLVYIVLTFLSGVLYFLSTAINPFLYNIMSNKFR<br>NAFKVRLN--
Bombyx mori	AEX31546.1	pheromone biosynthesis activating neuropeptide receptor isoform As	BomoPBANR_As	-MMADETVNMEM-LENN--LLNVTNVT-<br>DQSSAYSESYPLHLLVPLSVTYAVIFIVGILGNTSTCVVIARNRSMHTATNFYLFSLAISDIILLVCGLPLELYRLWNPFTYPLGEA<br>QCITIGLASETSANATVLTITAFTMERYIAICRPFMSHTMSKLSRAVRFIIAIWVFALCTAVPQAMQFGIVSYVENGQSMSACTV<br>KGPGVHQVFVISSFVFFVVPMSVISVLYALIGLKLRTSRILHPVKKLSLDSNERPG-<br>AHTPYRNGSSQRRVIRMLVAVALSFFICWAPFHVQRLLAIYGKSLEHPSDTFYLV---<br>---
Bombyx mori	AEX15643.1	pheromone biosynthesis activating neuropeptide receptor isoform B	BomoPBANR_B	-MMADETVNMEM-LENN--LLNVTNVT-<br>DQSSAYSESYPLHLLVPLSVTYAVIFIVGILGNTSTCVVIARNRSMHTATNFYLFSLAISDIILLVCGLPLELYRLWNPFTYPLGEA<br>QCITIGLASETSANATVLTITAFTMERYIAICRPFMSHTMSKLSRAVRFIIAIWVFALCTAVPQAMQFGIVSYVENGQSMSACTV<br>KGPGVHQVFVISSFVFFVVPMSVISVLYALIGLKLRTSRILHPVKKLSLDSNERPG-<br>AHTPYRNGSSQRRVIRMLVAVALSFFICWAPFHVQRLLAIYGKSLEHPSDTFYLVYIVLTFLSGVLYFLSTAINPFLYNIMSNKFR<br>NAFKMTLAAWCGRRGGPRMGRSYSALLASQRQRAANGLTDPVRGPRRLRRLSTATTHLCDAPPRAQCYQNRDLSIVNESPS<br>ASSHWSRVWRLR-NEPSDSIGSPRSISNSSLREVDDELTGEELATYMYHVNCNIEGLT---
Bombyx mori	AEX15640.1	pheromone biosynthesis activating neuropeptide receptor	BomoPBANR_C	-MMADETVNMEM-LENN--LLNVTNVT-<br>DQSSAYSESYPLHLLVPLSVTYAVIFIVGILGNTSTCVVIARNRSMHTATNFYLFSLAISDIILLVCGLPLELYRLWNPFTYPLGEA

		isoform C		QCITIGLASETSANATVLTITAFTMERYIAICRPFMSHTMSKLSRAVRFIIAIWVFALCTAVPQAMQFGIVSYVENGQSMSACTV<br>KGPGVHQVFVISSFVFFVVPMSVISVLYALIGLKLRTSRILHPVKKLSLDSNERPG-<br>AHTPYRNGSSQRRVIRMLVAVALSFFICWAPFHVQRLLAIYGKSLEHPSDTFYLVYIVLTFLSGVLYFLSTAINPFLYNIMSNKFR<br>NAFKMTLAAWCGRRGGPRMGRSYSALLASQRQRAANGLTDPVRGPRRLRRLSTATTHLCDAPPRAQVSATK----IAISP--------<br>--
Chilo suppressalis	ALM88337.1	pheromone biosynthesis activating neuropeptide receptor A	ChsuPBANR_A	-MDTI-LVDIPP-------VLNMTNHT-<br>MQTSGYAEPESLNLLVPLSITFCIIFVAGVLGNISTCVVISRNRSMHTATNFYLFSLAISDLILLICGLPIELYKMWNPATYPLGEP<br>VCIALGLASETSANATVLTITAFTVERYIAICRPFMSHTMSKLSRAVRYIILIWVCALCTAAPQAMQFGIVKESDNGETVTVCTV<br>KGQGVHQVFIISSFIFFVVPMSVISVLYALIGVKLRTSRVLHPVKKLFVENGERGNRTNMRYRSGTSQQRVIRMLVAVALSFFVC<br>WAPFHLQRLLAIYGKSMENPSDTFYTAYIILTLLSGVLYFLSPAINPILYNIMSNKFRNAFKV----------RNLPKFFM------------------<br>---
Chilo suppressalis	ALM88338.1	pheromone biosynthesis activating neuropeptide receptor B	ChsuPBANR_B	-MDTI-LVDIPP-------VLNMTNHT-<br>MQTSGYAEPESLNLLVPLSITFCIIFVAGVLGNISTCVVISRNRSMHTATNFYLFSLAISDLILLICGLPIELYKMWNPATYPLGEP<br>VCIALGLASETSANATVLTITAFTVERYIAICRPFMSHTMSKLSRAVRYIILIWVCALCTAAPQAMQFGIVKESDNGETVTVCTV<br>KGQGVHQVFIISSFIFFVVPMSVISVLYALIGVKLRTSRVLHPVKKLFVENGERGNRTNMRYRSGTSQQRVIRMLVAVALSFFVC<br>WAPFHLQRLLAIYGKSMENPSDTFYTAYIILTLLSGVLYFLSPAINPILYNIMSNKFRNAFKVTFTNWCGRR-<br>RGDPRFGRTYSAMLATQRLRDGLVTEHR-TRRLQRLSTATSTLFEAPPRAECFRGQELSAVNEFPSG-<br>TSWQQVWRLRHHYLSDSVASARSISNTSLPEVDEELTDDELATYMYQVNLKIRGLLLIR
Danaus plexippus	EHJ71920.1	pheromone biosynthesis-activating neuropeptide receptor	DaplPBANR	---------MDLDDEELQ-SLNETNDT---<br>QSGFAEPESLDVIVPLSVIYAIIFVTGILGNISTCVVIGRNRSMHTATNFYLFSLAISDLLLLICGLPLEVHRLWNPLSYPLGEALCI<br>TVGLISETSANATVLTITAFTVERYIAICRPFMSHKMSKLSRAVRYIIAIWICALCSAVPQAMQFGVVSYKENGQNISACTVKGH<br>GVHQVFVISSFVFFVAPMSLITVLYALIGLKLHTSRVLHPVKKSSVESGDRPN-<br>GTPRYRNGASQRRVIRMLVAVALSFFLCWAPFHVQRLIAIYGKNMEHPTDTFYKVYIVLTYVSGVLYFLSTSINPFLYNIMSNK<br>FRNAFKAMFGKLGITYNGH--<br>--
Helicoverpa armigera	AEX15647.1	pheromone biosynthesis activating neuropeptide receptor isoform A	HearPBANR_A	-MTLSAPPSIDDYEDPF--VMNTTNVT-<br>SHPAAYDEQYALDLVVPLTVTYVIIFVAGILGNTSTCVVIARNRSMHTATNFYLFSLAISDLILLVCGLPFEVHRLWNPDTYPLG<br>EAHCIAIGLASETSANATVLTITAFTVERYIAICRPFMSHTMSKLSRAVRFIIAIWVFALCTAVPQAMQFGIVSYVDHGQNVSAC<br>TVKGVGVHQVFVISSFVFFVVPMSMISVLYALIGIKLRTSRVLHPVKKLSVESNERP--<br>GQMQYRNGASQRRVIRMLVAVALSFFICWAPFHVQRLLAIYGKSLEHPSDTFYLVYIVLTFLSGVLYFLSTAINPFLYNIMSNKF<br>RNAFKVRVYD---
Helicoverpa armigera	AEX31547.1	pheromone biosynthesis activating neuropeptide receptor	HearPBANR_As	-MTLSAPPSIDDYEDPF--VMNTTNVT-<br>SHPAAYDEQYALDLVVPLTVTYVIIFVAGILGNTSTCVVIARNRSMHTATNFYLFSLAISDLILLVCGLPFEVHRLWNPDTYPLG

		isoform As		EAHCIAIGLASETSANATVLTITAFTVERYIAICRPFMSHTMSKLSRAVRFIIAIWVFALCTAVPQAMQFGIVSYVDHGQNVSAC<br>TVKGVGVHQVFVISSFVFFVVPMSMISVLYALIGIKLRTSRVLHPVKKLSVESNERP--<br>GQMQYRNGASQRRVIRMLVAVALSFFICWAPFHVQRLLAIYGKSLEHPSDTFYLVNYYYLIYKVIFYSFS----LFFLGIYRY-------<br>---
Helicoverpa armigera	AEX15644.1	pheromone biosynthesis activating neuropeptide receptor isoform B	HearPBANR_B	-MTLSAPPSIDDYEDPF--VMNTTNVT-<br>SHPAAYDEQYALDLVVPLTVTYVIIFVAGILGNTSTCVVIARNRSMHTATNFYLFSLAISDLILLVCGLPFEVHRLWNPDTYPLG<br>EAHCIAIGLASETSANATVLTITAFTVERYIAICRPFMSHTMSKLSRAVRFIIAIWVFALCTAVPQAMQFGIVSYVDHGQNVSAC<br>TVKGVGVHQVFVISSFVFFVVPMSMISVLYALIGIKLRTSRVLHPVKKLSVESNERP--<br>GQMQYRNGASQRRVIRMLVAVALSFFICWAPFHVQRLLAIYGKSLEHPSDTFYLVYIVLTFLSGVLYFLSTAINPFLYNIMSNKF<br>RNAFKVTLATWCGRGGPRMGRTYSALLASQRLRAGGPGACASVRGPRRLRRLSTATTQLYDAPPRAQCYNGRDLPTVNESP<br>SGNGHWGRAWRLRIHDPSDSVDSPRSISNSSLREVDDELTGEELATYMYHVNCNIGGLT---
Helicoverpa armigera	AEX15641.1	pheromone biosynthesis activating neuropeptide receptor isoform C	HearPBANR_C	-MTLSAPPSIDDYEDPF--VMNTTNVT-<br>SHPAAYDEQYALDLVVPLTVTYVIIFVAGILGNTSTCVVIARNRSMHTATNFYLFSLAISDLILLVCGLPFEVHRLWNPDTYPLG<br>EAHCIAIGLASETSANATVLTITAFTVERYIAICRPFMSHTMSKLSRAVRFIIAIWVFALCTAVPQAMQFGIVSYVDHGQNVSAC<br>TVKGVGVHQVFVISSFVFFVVPMSMISVLYALIGIKLRTSRVLHPVKKLSVESNERP--<br>GQMQYRNGASQRRVIRMLVAVALSFFICWAPFHVQRLLAIYGKSLEHPSDTFYLVYIVLTFLSGVLYFLSTAINPFLYNIMSNKF<br>RNAFKVTLATWCGRGGPRMGRTYSALLASQRLRAGGPGACASVRGPRRLRRLSTATTQLYDAPPRAQVSATTGAIYQPLTSP<br>LAGTG--TGAERGGYASTTPPTPSTPLAASPTPVFARLTTSSPARSWPPTCTT--------
Heliothis peltigera	AEQ33641.1	pheromone biosynthesis activating neuropeptide receptor	HepePBANR	-MTLSAPPSIDDYEESF--EMNTTNVT-<br>SHPAAYDEQYALDLVVPLTVTYVIIFVAGILGNTSTCVVIARNRSMHTATNFYLFSLAISDLILLVCGLPFEVHRLWNPDTYPLG<br>EAHCIAIGLASETSANATVLTITAFTVERYIAICRPFMSHTMSKLSRAVRFIIAIWVFALCTAVPQAMQFGIVSYVDHGQNVSAC<br>TVKGVGVHQVFVISSFVFFVVPMSMISVLYALIGIKLRTSRVLHPVKKLSVESNERP--<br>GQMQYRNGASQRRVIRMLVAVALSFFICWAPFHVQRLLAIYGKSLEHPSDTFYLVYIVLTFLSGVLYFLSTAINPFLYNIMSNKF<br>RNAFKV--
Heliothis virescens	ABU93812.1	pheromone biosynthesis-activating neuropeptide receptor isoform A	HeviPBANR_A	-MTLPAPPSIDEYEDPF--VMNTTNVT-<br>SHPAAYDEQYALDLVVPLTVTYVIIFVAGILGNTSTCVVIAKNRSMHTATNFYLFSLAISDLILLVCGLPFEVHKLWNPDTYPLG<br>EAHCIAIGLASETSANATVLTITAFTVERYIAICRPFMSHTMSKLSRAVRFIIAIWVFALCTAVPQAMQFGIVSYVDHGQNVSAC<br>TVKGVGVHQVFVISSFVFFVVPMSMISVLYALIGIKLRTSRVLHPVKKLSVESNERP--<br>GQMQYRNGASQRRVIRMLVAVALSFFICWAPFHVQRLLAIYGKSLEHPSDTFYLVYIVLTFLSGVLYFLSTAINPFLYNIMSNKF<br>RNAFKI--
Heliothis virescens	ABU93813.1	pheromone biosynthesis-activating neuropeptide receptor isoform B	HeviPBANR_B	-MTLPAPPSIDEYEDPF--VMNTTNVT-<br>SHPAAYDEQYALDLVVPLTVTYVIIFVAGILGNTSTCVVIAKNRSMHTATNFYLFSLAISDLILLVCGLPFEVHKLWNPDTYPLG<br>EAHCIAIGLASETSANATVLTITAFTVERYIAICRPFMSHTMSKLSRAVRFIIAIWVFALCTAVPQAMQFGIVSYVDHGQNVSAC

				TVKGVGVHQVFVISSFVFFVVPMSMISVLYALIGIKLRTSRVLHPVKKLSVESNERP--<br>GQMQYRNGASQRRVIRMLVAVALSFFICWAPFHVQRLLAIYGKSLEHPSDTFYLVYIVLTFLSGVLYFLSTAINPFLYNIMSNKF<br>RNAFKVTLATWCGRGGPRMGRTYSALLASQRLRAGGPGACTSVRGPRRLRRLSTATTQLYDAPPRAQCYNGRDLPTVNESP<br>SGNGHWGRAWRLRIHDPSDSVDSPRSISNSSLREVDDELTGEELATYMYHVNCNIGGLT---
Heliothis virescens	ABV58013.1	pheromone biosynthesis-activating neuropeptide receptor isoform-C	HeviPBANR_C	-MTLPAPPSIDEYEDPF--VMNTTNVT-<br>SHPAAYDEQYALDLVVPLTVTYVIIFVAGILGNTSTCVVIAKNRSMHTATNFYLFSLAISDLILLVCGLPFEVHKLWNPDTYPLG<br>EAHCIAIGLASETSANATVLTITAFTVERYIAICRPFMSHTMSKLSRAVRFIIVIWVFALCTAVPQAMQFGIVSYVDHGQNVSAC<br>TVKGVGVHQVFVISSFVFFVVPMSMISVLYALIGIKLRTSRVLHPVKKLSVESNERP--<br>GQMQYRNGASQRRVIRMLVAVALSFFICWAPFHVQRLLAIYGKSLEHPSDTFYLVYIVLTFLSGVLYFLSTAINPFLYNIMSNKF<br>RNAFKVTLATWCGRGGPRMGRTYSALLASQRLRAGGPGACTSVRGPRRLRRLSTATTQLYDAPPRAQVSATTGGIYQPLTSP<br>LVGTG--TGAERGGYASTTPPTPSTPLAASPTPVFARLTTSSPVKSWPPTCTT--------
Helicoverpa zea	AAP93921.1	pheromone biosynthesis-activating neuropeptide receptor	HezePBANR	-MTLSAPPSIDDYEDPF--VMNTTNVT-<br>SHPAAYDEQYALDLVVPLTVTYVIIFVAGILGNTSTCVVIARNRSMHTATNFYLFSLAISDLILLVCGLPFEVHRLWNPDTYPLG<br>EAHCIAIGLASETSANATVLTITAFTVERYIAICRPFMSHTMSKLSRAVRFIIAIWVFALCTAVPQAMQFGIVSYVDHGQNVSAC<br>TVKGVGVHQVFVISSFVFFVVPMSMISVLYALIGIKLRTSRVLHPVKKLSVESNERP--<br>GQMQYRNGASQRRVIRMLVAVALSFFICWAPFHVQRLLAIYGKSLEHPSDTFYLVYIVLTFLSGVLYFLSTAINPFLYNIMSNKF<br>RNAFKFKTTA---
Helicoverpa zea	AFP19101.1	pheromone biosynthesis-activating neuropeptide receptor isoform-B	HezePBANR_B	-MTLSAPPSIDDYEDPF--VMNTTNVT-<br>SHPAAYDEQYALDLVVPLTVTYVIIFVAGILGNTSTCVVIARNRSMHTATNFYLFSLAISDLILLVCGLPFEVHRLWNPDTYPLG<br>EAHCIAIGLASETSANATVLTITAFTVERYIAICRPFMSHTMSKLSRAVRFIIAIWVFALCTAVPQAMQFGIVSYVDHGQNVSAC<br>TVKGVGVHQVFVISSFVFFVVPMSMISVLYALIGIKLRTSRVLHPVKKLSVESNERP--<br>GQMQYRNGASQRRVIRMLVAVALSFFICWAPFHVQRLLAIYGKSLEHPSDTFYLVYIVLTFLSGVLYFLSTAINPFLYNIMSNKF<br>RNAFKVTLATWCGRGGPRMGRTYSALLASQRLRAGGPGACASVRGPRRLRRLSTATTQLYDAPPRAQCYNGRDLPTVNESP<br>SGNGHWGRAWRLRIHDPSDSVDSPRSISNSSLREVDDELTGEELATYMYHVNCNIGGLT---
Helicoverpa zea	AEO17028.2	pheromone biosynthesis-activating neuropeptide receptor isoform-C	HezePBANR_C	-MTLSAPPSIDDYEDPF--VMNTTNVT-<br>SHPAAYDEQYALDLVVPLTVTYVIIFVAGILGNTSTCVVIARNRSMHTATNFYLFSLAISDLILLVCGLPFEVHRLWNPDTYPLG<br>EAHCIAIGLASETSANATVLTITAFTVERYIAICRPFMSHTMSKLSRAVRFIIAIWVFALCTAVPQAMQFGIVSYVDHGQNVSAC<br>TVKGVGVHQVFVISSFVFFVVPMSMISVLYALIGIKLRTSRVLHPVKKLSVESNERP--<br>GQMQYRNGASQRRVIRMLVAVALSFFICWAPFHVQRLLAIYGKSLEHPSDTFYLVYIVLTFLSGVLYFLSTAINPFLYNIMSNKF<br>RNAFKVTLATWCGRGGPRMGRTYSALLASQRLRAGGPGACASVRGPRRLRRLSTATTQLYDAPPRAQVSATTGAIYQPLTSP<br>LAGTG--TGAERGGYASTTPPTPSTPLAASPTPVFARLTTSSPARSWPPTCTT--------
Mythimna separata	AEX15648.1	pheromone biosynthesis activating neuropeptide receptor	MasePBANR_A	MMTLSEPQDMEMEFVET--FVNMTNVT-<br>SQSAAYSEPYTLDLLVPLSITYAVIFVAGILGNTSTCIVIARNRSMHTATNFYLFSLAISDLILLVCGLPLELHRLWYPFTYPLGEA

		isoform A		ECITIGLASETSANATVLTITAFTVERYIAICRPFMSHTMSKLSRAVRFIVAIWVFALCTAVPQAMQFGLVSYVENGQTIVECTVK GPGVHQVFVISSFVFFVVPMSVITVLYALIGVKLRTSRVLHPVKKLSVDSNERPY- GQTQYRNGASQRRVIRMLVAVALSFFICWAPFHVQRLLAIYGKSLEHPSDTFYLVYIVLTFLSGVLYYLSTAINPFLYNIMSNKF RNAFK---VRK--
Mythimna separata	AEX31548.1	pheromone biosynthesis activating neuropeptide receptor isoform As	MasePBANR_B	MMTLSEPQDMEMEFVET--FVNMTNVT- SQSAAYSEPYTLDLLVPLSITYAVIFVAGILGNTSTCIVIARNRSMHTATNFYLFSLAISDLILLVCGLPLELHRLWYPFTYPLGEA ECITIGLASETSANATVLTITAFTVERYIAICRPFMSHTMSKLSRAVRFIVAIWVFALCTAVPQAMQFGLVSYVENGQTIVECTVK GPGVHQVFVISSFVFFVVPMSVITVLYALIGVKLRTSRVLHPVKKLSVDSNERPY- GQTQYRNGASQRRVIRMLVAVALSFFICWAPFHVQRLLAIYGKSLEHPSDTFYLVYIVLTFLSGVLYYLSTAINPFLYNIMSNKF RNAFKMTLANWCGRRGVPRMGRTYSALLASQRQRAMN- GSAERGRTRRLRRLSTATTQLGDAPPRAECYKDRDLSIVNESPSGNSQWSQAWRLR- ADPSDSIGSPRSISNSSLREVDEELTGEELATYMYNVN-DTGGPT---
Mythimna separata	AEX15645.1	pheromone biosynthesis activating neuropeptide receptor isoform B	MasePBANR_C	MMTLSEPQDMEMEFVET--FVNMTNVT- SQSAAYSEPYTLDLLVPLSITYAVIFVAGILGNTSTCIVIARNRSMHTATNFYLFSLAISDLILLVCGLPLELHRLWYPFTYPLGEA ECITIGLASETSANATVLTITAFTVERYIAICRPFMSHTMSKLSRAVRFIVAIWVFALCTAVPQAMQFGLVSYVENGQTIVECTVK GPGVHQVFVISSFVFFVVPMSVITVLYALIGVKLRTSRVLHPVKKLSVDSNERPY- GQTQYRNGASQRRVIRMLVAVALSFFICWAPFHVQRLLAIYGKSLEHPSDTFYLVYIVLTFLSGVLYYLSTAINPFLYNIMSNKF RNAFK--MTLANWCGRRGVPRMGRTYS-ALLAS- QRQRAMNGSAERGRTRRLRRLSTATTQLGDAPPRAEVS-ATKTATSL-
Mythimna separata	AEX15642.1	pheromone biosynthesis activating neuropeptide receptor isoform C	MasePBANR_D	MMTLSEPQDMEMEFVET--FVNMTNVT- SQSAAYSEPYTLDLLVPLSITYAVIFVAGILGNTSTCIVIARNRSMHTATNFYLFSLAISDLILLVCGLPLELHRLWYPFTYPLGEA ECITIGLASETSANATVLTITAFTVERYIAICRPFMSHTMSKLSRAVRFIVAIWVFALCTAVPQAMQFGLVSYVENGQTIVECTVK GPGVHQVFVISSFVFFVVPMSVITVLYALIGVKLRTSRVLHPVKKLSVDSNERPY- GQTQYRNGASQRRVIRMLVAVALSFFICWAPFHVQRLLAIYGKSLEHPSDTFYLVYIVLTFLSGVLYYLSTAINPFLYNIMSNKF RNAFK---CYKDRDLSIVNESPSGNSQWSQAWRLR- ADPSDSIGSPRSISNSSLREVDEELTGEELATYMYNVN-DTGGPT---
Manduca sexta	ACQ90219.1	pheromone biosynthesis-activating neuropeptide receptor subtype A	MysePBANR_A	-MTLPAPPSIDEYEDPF--VMNTTNVT- SHPAAYDEQYALDLVVPLTVTYVIIFVAGILGNTSTCVVIARNRSMHTATNFYLFSLAISDLILLVCGLPFEVHRLWNPDTYPLG EAHCIAIGLASETSANATVLTITAFTVERYIAICRPFMSHTMSKLSRAVRFIIAIWVCALCTAVPQAMQFGIVSYVDNGQNVSAC TVKGVGVHQVFVISSFVFFVVPMSMISVLYALIGIKLRTSRVLHPVKKLSVDSNERAS- GQMQYRNGASQRRVIRMLVAVALSFFICWAPFHVQRLLAIYGKSLEHPSDTFYLVYIVLTFLSGVLYFLSTAINPFLYNIMSNKF RNAFKVRFCNIFMIDLLVLLPRGTPFFILQPALLLIL--- ------------

Manduca sexta	ACQ90220.1	pheromone biosynthesis-activating neuropeptide receptor subtype B	MysePBANR_As	-MTLPAPPSIDEYEDPF--VMNTTNVT-<br>SHPAAYDEQYALDLVVPLTVTYVIIFVAGILGNTSTCVVIARNRSMHTATNFYLFSLAISDLILLVCGLPFEVHRLWNPDTYPLG<br>EAHCIAIGLASETSANATVLTITAFTVERYIAICRPFMSHTMSKLSRAVRFIIAIWVCALCTAVPQAMQFGIVSYVDNGQNVSAC<br>TVKGVGVHQVFVISSFVFFVVPMSMISVLYALIGIKLRTSRVLHPVKKLSVDSNERAS-<br>GQMQYRNGASQRRVIRMLVAVALSFFICWAPFHVQRLLAIYGKSLEHPSDTFYLVNL---<br>---
Manduca sexta	ACQ90221.1	pheromone biosynthesis-activating neuropeptide receptor subtype C	MysePBANR_B	-MTLPAPPSIDEYEDPF--VMNTTNVT-<br>SHPAAYDEQYALDLVVPLTVTYVIIFVAGILGNTSTCVVIARNRSMHTATNFYLFSLAISDLILLVCGLPFEVHRLWNPDTYPLG<br>EAHCIAIGLASETSANATVLTITAFTVERYIAICRPFMSHTMSKLSRAVRFIIAIWVCALCTAVPQAMQFGIVSYVDNGQNVSAC<br>TVKGVGVHQVFVISSFVFFVVPMSMISVLYALIGIKLRTSRVLHPVKKLSVDSNERAS-<br>GQMQYRNGASQRRVIRMLVAVALSFFICWAPFHVQRLLAIYGKSLEHPSDTFYLVYIVLTFLSGVLYFLSTAINPFLYNIMSNKF<br>RNAFKVTLATWCGRGGPRMGRTYSALLASQRLRAGGRGLRRRARDPR-<br>LRRLSTATTQLYDAPPRAQCYNGRDLPTVNESPSGNGHWARAWRLRIHDPSDSVDSPRSISNSSLREVDDELTGEELATYMYH<br>VNCNIGGLT---
Manduca sexta	ACQ90222.1	pheromone biosynthesis-activating neuropeptide receptor subtype D	MysePBANR_C	-MTLPAPPSIDEYEDPF--VMNTTNVT-<br>SHPAAYDEQYALDLVVPLTVTYVIIFVAGILGNTSTCVVIARNRSMHTATNFYLFSLAISDLILLVCGLPFEVHRLWNPDTYPLG<br>EAHCIAIGLASETSANATVLTITAFTVERYIAICRPFMSHTMSKLSRAVRFIIAIWVCALCTAVPQAMQFGIVSYVDNGQNVSAC<br>TVKGVGVHQVFVISSFVFFVVPMSMISVLYALIGIKLRTSRVLHPVKKLSVDSNERAS-<br>GQMQYRNGASQRRVIRMLVAVALSFFICWAPFHVQRLLAIYGKSLEHPSDTFYLVYIVLTFLSGVLYFLSTAINPFLYNIMSNKF<br>RNAFKVTLATWCGRGGPRMGRTYSALLASQRLRAGGRGLRRRARDPR-<br>LRRLSTATTQLYDAPPRAQVSATTGAIYQPLTSPRAGTG--<br>TGLARGASGSTTPPTPSTPLEASPTPVFARSTTSSPARSWPPTCTT--------
Operophtera brumata	KOB64247.1	pheromone biosynthesis-activating neuropeptide receptor	OpbrPBANR	-MTLPDQKTMEHDNGGAYYVLNSTNLT-<br>ASDSVFSEPNTLQIIVPLSLVYLVIFFAGILGNVSTCVVISRNRSMHTATNFYLFSLAISDLILLVCGLPLELYRLWNPYTYPLGEA<br>LCIIVGLASETTANATVLTIAAFTVERYIAICRPFMSHTMSKLSRAVRFIVAIWICAICTAVPQAMQFGIVYDDDNGQTTSACTV<br>KGRGVHQVFIISSFVFFVVPMSLITVLYALIGMKLRTSRVLHPVKKLSVDS-ERTSAP-<br>MQYRNGTSQRRVIRMLVAVALSFFICWAPFHI--------------------YIVLTYISGILYFLSTAIN---<br>--
Ostrinia nubilalis	AGL12066.1	pheromone biosynthesis activating neuropeptide receptor isoform A	OsnuPBANR_A	-MAPP-<br>SKAMEWNSSFDMLMANVTNASDSRSYAYAEPDSLDLLVPLSVTYIIIFVAGILGNISTCVVIARNRSMHTATNFYLFSLAISDLL<br>LLVCGLPIEFHRMWNPSTYPLGEAHCIALGLASETSANATVLTITAFTVERYIAICRPFMSHTMSKLSRAVRFIVAIWVCALCTA<br>IPQAMQFGIGTQIDNGQTTIVCTVKGQGVHQVFIISSFIFFVVPMSVICVLYALIGIKLRTSRVLHPIKKLSMESSERCSMS-<br>TRYRSGTSQRRVIRMLVAVALSFFVCWAPFHVQRLLAIYGKSMERPSETFYLVYIVLTFLSGVLYFLSTAINPILYNIMSNKFRN

				AFKVSGT---------VIIVTHSYFTFIVITLQT-GWNFAQP------EESKEVNH---<br>---------------
Ostrinia nubilalis	AGL12067.1	pheromone biosynthesis activating neuropeptide receptor isoform B	OsnuPBANR_B	-MAPP-<br>SKAMEWNSSFDMLMANVTNASDSRSYAYAEPDSLDLLVPLSVTYIIIFVAGILGNISTCVVIARNRSMHTATNFYLFSLAISDLL<br>LLVCGLPIEFHRMWNPSTYPLGEAHCIALGLASETSANATVLAITAFTVERYIAICRPFMSHTMSKLSRAVRFIVAIWVCALCTA<br>IPQAMQFGIVTQIDNGQTTIVCTVKGQGVHQVFIISSFIFFVVPMSVICVLYALIGIKLRTSRVLHPIKKLSMESSERCSMS-<br>TRYRSGTSQRRVIRMLVAVALSFFVCWAPFHVQRLLAIYGKSMERPSETFYLVYIVLTFLSGVLYFLSTAINPILYNIMSNKFRN<br>AFKMTLTNWCGR--SSAPRVGRTYSALLASQRQRNGIANSEPGMRRLRRLSTATTQLCDAPPRAKCFNGRELSAVSESPSG-<br>NSWARAWRLRLNEPSDSAASPPSISNSSLRDVDEELTGEELATYMYQVNCNLGKLT---
Ostrinia nubilalis	AGL12068.1	pheromone biosynthesis activating neuropeptide receptor isoform C	OsnuPBANR_C	-MAPP-<br>SKAMEWNSSFDMLMANVTNASDSRSYAYAEPGSLDLLVPLSVTYIIIFVAGILGNISTCVVIARNRSMHTATNFYLFSLAISDLL<br>LLVCGLPIEFHRMWNPSTYPLGEAHCIALGLASETSANATVLTITAFTVERYIAICRPFMSHTMSKLSRAVRFIVAIWVCALCTA<br>IPQAMQFGIVTQIDNGQTTIVCTVKGQGVHQVFIISSFIFFVVPMSVICVLYALIGIKLRTSRVLHPIKKLSMESSERCSMS-<br>TRYRSGTSQRRVIRMLVAVALSFFVCWAPFHVQRLLAIYGKSMERPSETFYLVYIVLTFLSGVLYFLSTAINPILYNIMSNKFRN<br>AFKMTLTNWCGR--SSAPRVGRTYSALLASQRQRNGIANSEPGMRRLRRLSTATTQLCDAPPRAKVSAS---MVVSCQP----------<br>--
Spodoptera exigua	ABY62317.2	pheromone biosynthesis-activating neuropeptide receptor	SpexPBANR	-MTLSAPP-IDEYEDPFI-VMNTTNVT-<br>SHPAAYDEPYTLDLVVPLTVTYVVIFVAGILGNTSTCVVIARNRSMHTATNFYLFSLAISDLILLVCGLPFEVHRLWNPDTYPLG<br>EAHCIAIGLASETSANATVLTITAFTVERYIAICRPFMSHTMSKLSRAVRFIIAIWVFALCTAVPQAMQFGIVSYKDNGQNVSAC<br>TVKGVGVHQVFVISSFVFFVVPMSMISVLYALIGIKLRTSRVLHPVKKLSVDSNERAS-<br>GQMQYRNGASQRRVIRMLVAVALSFFICWAPFHVQRLLAIYGKSLEHPSDTFYLVYIVLTFLSGVLYFLSTAINPFLYNIMSNKF<br>RNAFKVSFCYIYD---
Spodoptera littoralis	ABD52277.1	pheromone biosynthesis activating neuropeptide receptor	SplittPBANR	-MTLSAPP-IDEFEDPFV-VMNTTNVS-<br>SHPAAYDEPYTLDLVVPLTVTYVVIFVAGILGNTSTCVVIARNRSMHTATNFYLFSLAISDLILLVCGLPFEVHRLWNPDTYPLG<br>EAHCIAIGLASETSANATVLTITAFTVERYIAICRPFMSHTMSKLSRAVRFIIAIWVFALCTAVPQAMQFGIVSYVDNGQNVSAC<br>TVKGVGVHQVFVISSFVFFVVPMSMISVLYALIGIKLRTSRVLHPVKKLSVDSNERAS-<br>GQMQYRNGASQRRVIRMLVAVALSFFICWAPFHVQRLLAIYGKSLEHPSDTFYLVYIVLTFLSGVLYFLSTAINPFLYNIMSNKF<br>RNAFKVSFCYIHD---
Spodoptera litura	AJW32184.1	pheromone biosynthesis activating neuropeptide receptor	SplituPBANR	-MTLSAPP-IDEYEDPFV-VMNTTNVS-<br>SHPAAYDEPYTLDLVVPLTVTYMVIFVAGILGNTSTCVVIARNRSMHTATNFYLFSLAISDLILLVCGLPFEVHRLWNPDTYPLG<br>EAHCIAIGLASETSANATVLTITAFTVERYIAICRPFMSHTMSKLSRAVRFIIAIWVFALCTAVPQAMQFGIVSYVDNGQNVSAC<br>TVKGVGVHQVFVISSFVFFVVPMSMISVLYALIGIKLRTSRVLHPVKKLSVDSNERAS-<br>GQMQYRNGASQRRVIRMLVAVALSFFICWAPFHVQRLLAIYGKSLEHPSDTFYLVYIVLTFLSGVLYFLSTAINPFLYNIMSNKF

		RNAFKVSFCYIHD--
Plutella xylostella	PlxyPBANR	-MTLATLNVIQDDLALL--LGNLTNVT-<br>VRASAYSEPESLDILVSMTVVYAVIFVSGLLGNASTCVVIARNRSMHTATNFYLFSLAVSDLLLLICGLPLELHRLWNPVTFPFG<br>EIACIGLGLASETSTNATVLTITAFTVERYIAICHPFMSHTMSKLSRAVRFIIGIWIFAICMAFPQAMQFGIVALP--<br>GHDESACTVKGHGVHQVFVISSFVFFVVPMSVISVLYAQIGLKLRHSSVLHPVKKLSVES---GR-<br>GARSYRSGASQRRVIRMLVAVALSFFVCWAPFHVQRLLAIYGKNLDHPSDTFVLVYVVLNYISGILYFLSTAINPILYNIMSNKF<br>REAFKV---

Supplementary Table S2. Sequence details used in the alignment and phylogenetic analysis of Des genes.

Species name	Accession number	Sequence definition	Abbreviation	Amino acid sequence
Antheraea pernyi	ADO85596.1	acyl-CoA-delta-11-desaturase	AperΔ11	IV-YMNLFTFGYWYI-AGLYGLYLFFT---STKWS-TIILNIFLIIASGLGITVGAHRLWAHKCFKAKLPL--<br>QIILMILNTLAFQNTAITWVRDHRMHHKFTDT---DAD-PHNATRGFFFSHVG---WLLVKK-----HPELMKRSKHIDMS--<br>DIYGNPVLTFQKKYAFPLVATFTFILPTVVPIYFWDESLNNAWHAT-<br>VFRYAYNLNITFLVNSAAHMWGYRPYDKNI**[QPTQ]**NIFTTLCTFGEGFHNYHHTFPW--DYRAAELG-<br>NNYLNLSTKVIDFFAWIGWAYDLKTVPADIVN
Antheraea pernyi	ADO85598.1	acyl-CoA-delta-9-desaturase, partial	AperΔ9	FV-WRNIILFAYLHI-AALYGGYLFLF---HAKWQ-TDIFAYILYVMSGLGITAGAHRLWAHKSYKAKWPL--<br>RLILVIFNTLAFQDSAIDWARDHRMHHKYSET---DAD-PHNASRGFFFSHIG---WLLVRK-----HPELKRKGKGLDLN--<br>DLYADPILRFQKKYYLLLMPIACFILPTVIPVYFWSETWSNAFFVAALFRYTFILNVTWLVNSAAHKWGDKPYDKSI**[KPSE]**NISVSVFALG<br>EGFHNYHHTFPW--DYKTAELG-NNRLNFTTNFINFFAKFGWAYDLKTVSDEIVQ
Amyelois transitella	NP_001299594.1	acyl-CoA Delta(11) desaturase-like	AtraΔ11_1	IV-YRNLLTFGYWHL-SAVYGLYLCFT---SAKRA-TIIFAIFIYIIAKIGITGGAHRLWAHRAYKAKLPL--<br>EILLLIMNSIAFQDTVLTWARDHRLHHKYSDT---DAD-PHNPTRGFFFAHMG---WVLVRK-----HPEVLARGKALPID--<br>DLKNNPLLRFQKKYAIPMIGTLCFLMPTFVPVYFWGESISTAWNIN-<br>LLRYVTNLNVIFLINSWAHLIGNKPYDKNI**[AATQ]**SIPISIATLGEGFHNYHHTYPW--DYKASELG-<br>NNKINLTTKFIDFFAWVGWAYDLKSMPSEGVA
Amyelois transitella	XP_013183656.1	PREDICTED: acyl-CoA Delta(11) desaturase-like	AtraΔ11_2	IK-WTSFIAITLYHI-LGVYWCYHYAF---PVKWP-TLVFAAIMYVASGFGITGGAHRYWTHKAYKAKLPL--<br>KLFLMVCFASAGQNSIKQWVRDHRIHHKFSDT---EAD-PHNANRGLFFSHIG---WLMMKK-----NDEVLQAGKQIDMS--<br>DIENDVHLQIFEKYFNYIKLVFCYILPTTIGIWLWGEDWRCSVAWQCFIRFLTMFHSELTVNSLAHAYGYKPYNRNI**[IPAE]**NRFVATCTLGE<br>GWHNYHHAFPF--DYKAAEH--FDFLNFGTWFIQFFEKIGWAYDLRQATPGMIN
Amyelois transitella	XP_013185302.1	PREDICTED: acyl-CoA Delta(11) desaturase-like	AtraΔ11_3	I--HPLFFPFVLIYTTCGIYGGFLLMF----AKPQ-TILFTLFLVFPSMLGLGAGVHRLWSHRAFKVKPQL--<br>EILLIFFYLLANQRSIVTWARRHRLHHQCSDT---DAD-PHNATRGFFFSQFG---WMCVEP-----HPEAQKREKYIDVS--<br>DLMNNPIIRFQEKYITPLLLLVAYFIPTYIPT-LWGESLYVSFFAN-<br>IFRTNLVLFMINLINSAAHLIGYKPIDATA**[VGTQ]**YQGLGLIIFGEGFHNYHHTFPY--DYRSSEFG-<br>DIKYNLSALFIDFMAKIGWAYDLKVTSESVIK
Amyelois transitella	XP_013187169.1	PREDICTED: acyl-CoA Delta(11) desaturase-like isoform X1	AtraΔ11_4	IR-WKDTVLIALLHIVAITWFSYTVCFI-HSPKWQ-SILFAFLMGQIAGFGVTAGAHRYWCHRSYKAKLPL--<br>QFILLISYSIAGQNTIYNWVRDHRVHHKFSES---SAD-PHDARRGFFFSHVG---WLMMKK-----HPAVLKEGAKIDMS--<br>DIKNDPLVRFHTKYFNLFKMACCFLLPTLVPVIFWDETWDLAILSQPLLRYMFSLNFTWSVNSFAHIWGNKPYDRNI**[MPAE]**NWGVSVVA<br>MGEGWHNYHHTFPW--DYKAAELG--YTLNLTTLILDMFASIGWAYNLKVASSSLVH
Amyelois transitella	XP_013187170.1	PREDICTED: acyl-CoA Delta(11) desaturase-like	AtraΔ11_5	II-YHVVVVLLVSHL-FGFYGGYLLVK---KAKIQ-TIIFTVFLTSISMLGITAGAHRLWSHRAYKAKTPL--<br>QILLAIFFLLTSQRSIVTWVENHRLHHRFSDT---DGD-PHNATRGFFFSTIG---WTAVEP-----HPIVVREKKLIDMT--<br>DLLQNPIIRFQHKYIEIILIVVAYVIPTYIPT-LWGESLYNAYFIN-

		isoform X2		FFRLVVVLTIAGLINSVAHIWGYKPIDKTV**[VGTQ]**FLAVGVLAFGEGFHNYHHVFPY--DYRTSEVG-<br>DTKYNFTALFIDLMAKIGWAYDLKAVPEETVR
Amyelois transitella	XP_013187171.1	PREDICTED: acyl-CoA Delta(11) desaturase-like isoform X3	AtraΔ11_6	YV-WFNILWFLYLHV-ASLYGVYLALT---SAKWQ-TNVFAFAVHVMCAIGIGAGSHRLWTHRCFKAKTPL--<br>RILLMIWQTMGFQDCIFEWARDHRTHHKYADT---DAD-PHNAERGLFYSHMG---WLCCKK-----TPEVIEGGRRIDLT--<br>DLYADPVVMFQKKHYMKMMPILCFVVPTIIPVYFWGESWINAFFIPTILRYTIGINVVWSINSFAHTFGYRPYDKSL**[NPRD]**NIGTWMICV-<br>EGFHNYHHTFPW--DYRATEYPLYNMLTPTIVFIEAMAKIGQAYDLKSVSPEIIR
Amyelois transitella	XP_013187194.1	PREDICTED: acyl-CoA Delta(11) desaturase isoform X1	AtraΔ11_7	LV-KHNVIKFGYMHL-AALYGVYLCFT---SAKWQ-TLLWAFLLLEFAKIGITAGAHRLWCHRSYKAKLPL--<br>EIILLIFNSIAYMNTATYWVRDHRVHHKFADT---DAD-PHNVNRGFWFSQIG---WLFVRK-----HPDVVEKGKTVFMD--<br>DIHKNPLLRFQKKYAFFVIGLWAYVIPTVVPMYFWGESLNNSWHICTMLRHVLTINQIFLVNSIGHSWGNKPYDKNI**[RAVE]**NIAVSLMSTG<br>ECFHNYHHVFPF--DYKASELG-MTKFNAATMFINFFAWLGWAYDLKTIPDELII
Amyelois transitella	XP_013187195.1	PREDICTED: acyl-CoA Delta(11) desaturase isoform X2	AtraΔ11_8	IV-YRNLLTFGYWHL-SAVYGLYLCFT---CAKWA-TILFAFFLYVIAEIGITGGAHRLWAHRTYKAKLPL--<br>EILLLIMNSIAFQDTAFTWARDHRLHHKYSDT---DAD-PHNATRGFFYSHVG---WLLVKK-----HPEVKARGKYLSLD--<br>DLKNNPLLKFQKKYAILVIGTLCFLMPTFVPVYFWGEGISTAWNIN-<br>LLRYVMNLNMTFLVNSAAHIFGNKPYDKSI**[ASVQ]**NISVSLATFGEGFHNYHHTYPW--DYRAAELG-<br>NNRLNMTTAFIDFFAWIGWAYDLKSVPQEAIA
Amyelois transitella	XP_013190816.1	PREDICTED: acyl-CoA Delta(11) desaturase-like	AtraΔ11_9	--MLGITAGAHRLWSHRAYKAKTPL--QILLAIFFLLTSQRSIVTWVENHRLHHRFSDT---DGD-<br>PHNATRGFFFSTIG---WTAVEP-----HPIVVREKKLIDMT--DLLQNPIIRFQHKYIEIILIVVAYVIPTYIPT-LWGESLYNAYFIN-<br>FFRLVVVLTIAGLINSVAHIWGYKPIDKTV**[VGTQ]**FLAVGVLAFGEGFHNYHHVFPY--DYRTSEVG-<br>DTKYNFTALFIDLMAKIGWAYDLKAVPEETVR
Amyelois transitella	XP_013192760.1	PREDICTED: stearoyl-CoA desaturase 5 isoform X1	AtraΔ5_1	IV-WRNVFAFVYLHV-AATYGFYLMFTG--RVRIW-TILFAISFAVISAMGVTAGAHRLWAHRAYKAKWPL--<br>RLFLAILQTMAFQNHIYEWVRDHRVHHKFTET---DAD-PHNAKRGFFFSHIG---WLMVRK-----HKEVFEKGATVDMS--<br>DLEKDPIVMFQKKTYLFVMPIVCFVIPAWLPVYFWGEDPWTSWYAASIARYTLTLHFTWLVNSAAHIWGNRPYDKYI**[GATD]**NKTVAICA<br>FGEGWHNYHHVFPW--DYKAAELG-NYSTNMSTALIDIAAKYGLAYDLKTVSADMIR
Amyelois transitella	XP_013192762.1	PREDICTED: stearoyl-CoA desaturase 5 isoform X2	AtraΔ5_2	VRIW-------------------------------TILFAISFAVISAMGVTAGAHRLWAHRAYKAKWPL--RLFLAILQTMAFQNHIYEWVRDHRVHHKFTET---<br>DAD-PHNAKRGFFFSHIG---WLMVRK-----HKEVFEKGATVDMS--<br>DLEKDPIVMFQKKTYLFVMPIVCFVIPAWLPVYFWGEDPWTSWYAASIARYTLTLHFTWLVNSAAHIWGNRPYDKYI**[GATD]**NKTVAICA<br>FGEGWHNYHHVFPW--DYKAAELG-NYSTNMSTALIDIAAKYGLAYDLKTVSADMIR
Bicyclus anynana	AGD98720.1	delta11-desaturase	BanyΔ11_1	II-YFNLLTFGYAHL-ATLYGVYLACT---TATWK-TLMFHHVMFILAAVGITAGTHRLWSHRAYKAKMPL--<br>QIILMVLNSFAFQNSAIQWVRDHRMHHRYSDT---DAD-PHNATRGFFYSHMG---WLLVRK-----HPEMLRRGKFIDMS--<br>DIYANPVLRFQKKYAIPVFGTLCFALPTLIPMYFFGETLNTAWHLT-<br>IMRYVFNLHMTFLVNSAAHLWGNKPYDKNM**[LPAQ]**NLPVSFIAFGEGFHNYHHAFPW--DYKTAELG-<br>NNWLNFSTKFIDFFAWIGWAYDLKEVPLEVAR
Bicyclus anynana	AKJ32408.1	delta-11 desaturase-	BanyΔ11_2	II-YFNLLTFGYAHL-ATLYGMYLACT---TATWK-TLMFHHVMFILAAVGITAGTHRLWSHRAYKAKMPL--

		like protein		QIILMVLNSFAFQNSAIQWVRDHRMHHRYSDT---DAD-PHNATRGFFYSHMG---WLLVRK-----HPEMLRRGKFIDMS--<br>DIYANPVLRFQKKYAIPVFGTLCFALPTLIPMYFFGETLNTAWHLT-<br>IMRYVFNLHMTFLVNSAAHLWGNKPYDKNM**[LPAQ]**NLPVSFIAFGEGFHNYHHAFPW--DYKTAELG-<br>NNWLNFSTKFIDFFAWIGWAYDLKEVPLEVAR
Bicyclus anynana	AGD98721.1	delta9-desaturase	BanyΔ9	IV-WRNVILFMLLHL-GGLYGAYLFLT---QAMWT-TRIFTVLLYLSSGLGITAGAHRLWAHKSYKAKLPL--<br>RMLLTLFNTLAFQDSVLDWARDHRMHHKYSET---DAD-PHNATRGFFFSHVG---WLLVRK-----HPEIKAKGHTIDMS--<br>DLWADPVLRFQKKYYLILMPLVCFILPTYIPT-<br>LWGESLWNAYFVSAIFRYVYVLNVTWLVNSAAHKWGSKPYDKFI**[NPVE]**TKSVSLVVLGEGFHNYHHTFPW--DYKTAELG-<br>NYSLNFSKLFIDTMAKIGWAYDLKTVSPDVIE
Bombyx mori	ABD36148.1	acyl-CoA delta-11 desaturase isoform 1	BmorΔ11_1	IV-YKNIFIHIYLHV-TMFYGLYLCCT---TAKWP-SVGFAYLCYVAATIGVTAGAHRLWSHKSYKARLPL--QILLMVFLSLANQRAT-<br>HWVRDHRVHHKYSDT---DAD-PHNASRGFFYSHIG---WLFVRK-----HPEVKKKGKLIDLS--<br>DLFDNPALMFQHRYSKTFIPIVCFGLPTILPVILWDELLVVAWNLT-<br>IMRYVINFHVFFLVNSVAHIWGNKPYDKTI**[KSAE]**NNLVAFATLGEGYHNYHHVFPW--DYRCTELG-<br>RTWLNYTKLFIDLCAKVGLAYDLKVVSDDVIL
Bombyx mori	XP_004925564.1	PREDICTED: acyl-CoA Delta(11) desaturase	BmorΔ11_10	IK-WPSFIIITLYHV-IGAYWCYNFAF---PVKWQ-TVVFAAIMYVATGFGITGGAHRLWTHKSYKAKLPL--<br>KLFLLLCFSAAGQNSLYQWVRDHRIHHKFSDT---DAD-PHNANRGLFFSHIG---WLMMKK-----NNEVILRGKQIDMS--<br>DIENDPFLRFYDKNFNSLKLVFCYIPPTMLGILLWNEEWKCAVAWQCFIRFLGMFHSELTVNSLAHTFGYKPYNKNI**[VPAE]**NRFVSICTLG<br>EGWHNYHHAFPF--DYKAAEH--FDFFNFGTKFIKLFEKIGWAYDLKEATPEMIN
Bombyx mori	XP_004933819.1	PREDICTED: acyl-CoA Delta(11) desaturase	BmorΔ11_11	LV-WFNIIWFLFLHI-SSLYGLYLVFT---SAKWQ-TNVFAFGVHLMCAIGIGAGSHRLWTHRSFKARTPL--<br>RIVLMIWQTMGFQDCIFEWARDHRTHHKYADT---DAD-PHNAERGLFFSHMG---WLCCKK-----SPEVIEGGRRIDLS--<br>DLYADPVVMFQKKHYMKMMPILCFVLPTVIPVYFWDETWLNAFFIPTILRYTCGINVVWSVNSFAHTFGYRPYDKSL**[NPRE]**NIGVWMIC<br>V-EGFHNYHHTFPW--DYRATEHPLYNMLTPTIVFIDAMAMIGQAYDLKTVPHDIIK
Bombyx mori	XP_012547022.1	PREDICTED: acyl-CoA Delta(11) desaturase-like	BmorΔ11_12	VV-YRNIIVLLYYHM-AGIYGLYLGLI---AAKWA-TVISAFITYCISVIGVTAGSHRLWSHKSYKATKPL--<br>QLFLMLCQSVSNQHSVAYWVMVHRLHHKYSDS---DAD-PHNATRGLFYSHMG---WLMVRK-----HPEIEKRGKLLDLS--<br>DIYGNPYLKFQDTHYYWFLPLMSFLLPTIIPMYFWGETCSVAWNVN-<br>MCRYVMNLNAIFLVNSVAHMWGYKPYDKNI**[APSQ]**NLSVSVLSLGEGFHNYHHVFPW--DYKCDELS-<br>SFRTNPTTVFIELCAKLGLAYDLRSASDDLIE
Bombyx mori	ABD36149.1	acyl-CoA delta-11 desaturase isoform 2	BmorΔ11_2	IV-YKNIFIHIYLHV-TMFYGLYLCCT---TAKWP-SVGF-------------AGAHRLWSHKSYKARLPL--QILLMVFLSLANQRAT-<br>HWVRDHRVHHKYSDT---DAD-PHNASRGFFYSHIG---WLFVRK-----HPEVKKKGKLIDLS--<br>DLFDNPALMFQHRYSKTFIPIVCFGLPTILPVILWDELLVVAWNLT-<br>IMRYVINFHVFFLVNSVAHIWGNKPYDKTI**[KSAE]**NNLVAFATLGEGYHNYHHVFPW--DYRCTELG-<br>RTWLNYTKLFIDLCAKVGLAYDLKVVSDDVIL
Bombyx mori	NP_001274329.1	acyl-CoA Delta(11)	BmorΔ11_3	---MTIFSGIGITCGVHRLWTHRSYKVKAPM--KFLLMALFCSSGQNSIYNWVRDHRLHHKFADT---DAD-

		desaturase-like		PHNVKRGFFFSHIG---WLMVKK-----NESVLTKGKLIDMS--<br>DIESDSHLMFEHKYHNQLTILFCFLIPTLMNIFLIGEDWKCAIAWQCFIRYLYVLHCELTVNSLAHMYGYKPYNENI**[EASE]**NILVSVLTYGE<br>GYHNFHHVFPF--DFRAAET--MDFFSLSTKIIRTFEKIGWTYDLKQASPEMIE
Bombyx mori	NP_001274330.1	acyl-CoA Delta(11) desaturase-like	BmorΔ11_4	IR-WTNAILITTFHVVTVVWGFYTLFYEKQKPMWQ-SVIFGIFMGQLAGFGVTAGAHRYWCHRSYKAKLPL--<br>QLILALCYSAAGQNTIYEWVRDHRVHHKFSET---SAD-PHDANRGFFFSHIG---WLMMKK-----HPDVVKQGSKIDMS--<br>DIIHDPVVIFCTKYFILFKITFCWIIPSALPVYAWGELWNIAILSQAFWRYMLSLHFTWSVNSFAHLWGNKPYDRYI**[MPCE]**NKGVSIVAMG<br>EGWHNYHHTFPW--DYKAAELG--VPNNTTTFLLDLFARIGWAYDLKQASPALVR
Bombyx mori	NP_001296477.1	acyl-CoA Delta(11) desaturase-like	BmorΔ11_5	---MIIVSGAGITCGVHRLWTHRSYKAKAPM--KLLLMACFCSSGQNSIYNWVRDHRLHHKFADT---DAD-<br>PHNVKRGFFFSHIG---WLMVKK-----NESVLTKGKLIDMS--<br>DIESDSHLMFEHKYHNQLTILFCFLIPTLMNIFLIGEDWKCAVAWQCFIRYLYVLHCELTVNSLAHMYGYKPYNVNI**[EASE]**NILVSVLTYGE<br>GYHNFHHVFPF--DFRAAET--MDFFSLSTKIISTFEKIGWTYDLKQASPEMIE
Bombyx mori	NP_001296494.1	acyl-CoA Delta(11) desaturase	BmorΔ11_6	II-YFNIFTFTFAHL-SALYGVYLCFT---SAKRE-SLILGYIIGVLAGFGVTAGAHRLWTHRTYKAKMPL--<br>QILLMLFYSFAYQNSAVHWIRDHRLHHKYSDT---DAD-PHNATRGFFYSHMG---WLLVKK-----HPEVKRRGRSLDMS--<br>DIYSNRVLMFQKKYAIPITGTICFVIPTLLPMYLWGESFKNAWHIT-<br>ILRYIITLHVAFLVNSAAHIWGYKPYDKRI**[LPTQ]**NLFVSFMAFGEGFHNYHHVFPW--DYRTAELG-<br>NNYLNLTTMFIDFFAWLGWAYDLKSVPVSLAE
Bombyx mori	NP_001296508.1	acyl-CoA Delta(11) desaturase-like	BmorΔ11_7	IV-YKNIFIHIYLHV-TMFYGLYLCCT---TAKWP-SVGFAYLCYVAATIGVTAGAHRLWSHKSYKARLPL--QILLMVFLSLANQRAT-<br>HWVRDHRVHHKYSDT---DAD-PHNASRGFFYSHIG---WLFVRK-----HPEVKKKGKLIDLS--<br>DLFDNPALMFQHRYSKTFIPIVCFGLPTILPVILWDELLVVAWNLT-<br>IMRYVINFHVFFLVNSVAHIWANKPYDKTI**[KSAE]**NNLVAFATLGEGYHNYHHVFPW--DYRCTELG-<br>RTWLNYTKLFIDLCAKVGLAYDLKVVSDDVIL
Bombyx mori	XP_004923568.2	PREDICTED: acyl-CoA Delta(11) desaturase-like	BmorΔ11_8	IK-WDSLLVISFFHI-FGFYWCSKYAI---PLKLG-TFLFAYAMTVFCALGITCGAHRLWTHRSYKVKAPV--<br>KFLLMACFCSTGQNSIYDWVRDHRLHHKFADT---DAD-PHNVNRGFFFSHIG---WLMMKK-----NESVLTKGKLIDMS--<br>DIESDSHLMFQHKYHNQLKILFCFLIPTLMNIFLIGEDWKCAVAWQCFIRYLYVLHCELTVNSLAHMYGYKPYNVNI**[EASE]**NILVSVLTYG<br>EGYHNFHHVFPF--DFRAAET--MDFFSLSTKIIRTFEKIGWTYDLKQASPEMIE
Bombyx mori	XP_004923569.1	PREDICTED: acyl-CoA Delta(11) desaturase-like	BmorΔ11_9	VV-YRNIIVLLYYHM-AGIYGLYLGLI---AAKWA-TVISAFITYCISGIGVTAGSHRLWSHKSYKATKPL--<br>QLFLMLCQSVSNQHSVAYWVMVHRLHHKYSDS---DAD-PHYATRGLFYSHMG---WLMVRK-----HPEIEKRGKLLDLS--<br>DIYGNPYLKFQDTHYYWFLPLMSFLLPTIIPMYFWGETCSVAWNVN-<br>MCRYVMNLNAIFLVNSVAHMWGYKPYDKNI**[APSQ]**NLSVSVLSLGEGFHNYHHVFPW--DYKCDELS-<br>SFRTNPTTVFIELCAKLGLAYDLRSASDDLIE
Bombyx mori	XP_004930794.1	PREDICTED: sphingolipid delta(4)-desaturase	BmorΔ4	LFGYDPLFKWVVLSMVLAQFAMMPIVQ---<br>HLSWPMTLILAYCFGGVINHSLMLAIHEIAHNLAFGHNRPLHNRLFGFFANLPIGVPISISFKKYHLEHHRYQGDEVIDTDLPTLLEAKLFCT<br>TGGKLCWLFLQPFFYALRPLVVRPKPPTPLE-----------------LINLIIQLFFDAVVVKLFGW-

		DES1		KVLGYLVFGSLMAMGVHPVAGHFISEHYMFRKGFETYSYY------GPLNWITFNVGYHNEHHDFPAVPGRRLPEVK-RIASEFYDTLPQHNSWSSVLYDFVMDPEIGPY
Bombyx mori	NP_001296524.1	stearoyl-CoA desaturase 5-like	BmorΔ5_1	IV-WRNVFAFIYLHA-ATLYGIYLMFTG--KVKLW-TVLFAVSFTLLSAVGITVGAHRLWAHRAYKAKWPL--RLFLALLQTMAFQNHIYEWVRDHRVHHKFTET---DAD-PHNAKRGFFFSHMG---WLMVRK-----HKDVFEKGAAVDMS--DLEKDPIVMFQKKTYLFVMPILCFLLPLYIPVYFWNENPWSSWYVASMFRYTISLHFTWLVNSAAHLWGNKPYDQYI**[GATD]**NKTVAICALGEGWHNYHHVFPW--DYKAAELG-DYSTNLSTALIDIAAKYGLAYDLKTVSEKMIR
Bombyx mori	XP_004925565.1	PREDICTED: stearoyl-CoA desaturase 5	BmorΔ5_2	IR-WPDLVVQVSLHL-VSIYGIFLIVTN--EVKLL-TTLFALATIYTSGFGITAGVHRLWSHRAYKARTPL--RILLAFLFTITGQRDIYTWALDHRVHHKYSET---VAD-PHDVRRGFWFAHVG---WLVLTP-----HPAVEDRRVALRKCSLDLIEDPVVRIQQKIFIPLFLLLNILIPIWIPWYFWNESLVTSFVISFVLRFTTTLNIAFSVNSFAHLWGNKPYDRFI**[KPAE]**NSVVSLAALGEGWHNYHHVFPW--DYRTSELG---RINVSTNFIDFFAKIGWAYDLKAATSSMIE
Bombyx mori	XP_012545304.1	PREDICTED: stearoyl-CoA desaturase 5	BmorΔ5_3	IV-WKNAIGYLILHL-LCLWGVGLIVT---ELEGLKSCLWTILHMYAGSTGVTVGAHRLFTHKSFKATPLL--KVFLLCLQTIAGQNSTFIWVRDHRLHHRYSDT---DAD-PHNSKRGFFFCHIG---WLMMKK-----HPYVIELGRRIDMS--DLQADKMIMFQKKYYYYLYFMVAFLIPVFVPIYFFNEHWLSSFLVCYCARYILQLNLTWLVNSAAHMYGTRPYDRKL**[QPVE]**SWFVSCISLGEGWHNYHHAFPW--DYKAAEES--MHFNCNATIIRFFERIGLAYDLKTASPDMIR
Choristoneura parallela	AAQ12891.1	E11-desaturase SFWGE11	CparΔ11_1	II-YTNLLTFGYGHI-AGLYGLYLCFT---SAKWQ-TVILAIILNEMAILGITAGAHRLWSHRSYKAAVPL--QIILMIFNSLAFQNSAINWVRDHRMHHKYSDT---DGD-PHNASRGFFYSHVG---WLLVKK-----HPEVKKRGKMIDMS--DIYSNPVLRFQKKYAIPFIGMICFVLPTIIPMYFWGETLSNAWHIT-MLRYVFSLNSIFLVNSAAHLYGYRPYDKNI**[LPAE]**NKIALIACLGDSFHNYHHVFPW--DYRASELG-NIGMNWTAQFIDFFAWIGWAYDLKTASDENIN
Choristoneura parallela	ADC53486.1	desaturase E11, partial	CparΔ11_2	--------------I-AGLYGLYLCFT---SAKWQ-TVILAIILNEMAILGITAGAHRLWSHRSYKAAVPL--QIILMIFNSLAFQNSAINWVRDHRMHHKYSDT---DGD-PHNASRGFFYSHVG---WLLVKK-----HPEVKKRGKMIDMS--DIYSNPVLRFQKKYAIPFIGMICFVLPTIIPMYFWGETLSNAWHIT-MLRYVFSLNSIFLVNSAAHLYGYRPYDKNI**[LPAE]**NKIALIACLGDSFHNYHHVFPW--DYRASELG-NIGMNWTAQFIDFFAWIGWAYDLK--------
Choristoneura parallela	AAN39700.1	Z9-desaturase SFWG1A	CparΔ9_1	YV-WRNIILFAYLHL-AAIYGGYLFLF---SAKWQ-TDIFAYLLYVASGLGITAGAHRLWAHKSYKAKWPL--RLILTIFNTIAFQDSAIDWARDHRMHHKYSET---DAD-PHNATRGFFFSHIG---WLLVRK-----HPELKRKGKGLDLS--DLYSDPILRFQKKYYMILMPLACFILPTVIPVYMWNETWSNAFFVAALFRYTFILNVTWLVNSAAHKWGDKPYDKSI**[KPSE]**NMSVSLFAFGEGFHNYHHTFPW--DYKTAELG-NHRLNFTTKFINFFAKIGWAYDMKTVSQEIVQ
Choristoneura parallela	AAN39701.1	Z9-desaturase SFWG1B	CparΔ9_2	YV-WRNIILFAYLHL-AAIYGGYLFLF---SAKWQ-TDIFAYLLYVASGLGITAGAHRLWAHKSYKAKWPL--RLILTIFNTIAFQDSAIDWARDHRMHHKYSET---DAD-PHNATRGFFFSHIG---WLLVRK-----HPELKRKGKGLDLS--DLYSDPILRFQKKYYMILMPLACFILPTVIPVYMWNETWSNAFFVAALFRYTFILNVTWLVNSAAHKWGDKPYDKSI**[KPSE]**NMSVSLFAFGEGFHNYHHTFPW--DYKTAELG-NHRLNFTTKFINFFAKIGWAYDMKTVSQEIVQ
Choristoneura	AAQ12887.1	Z9-desaturase	CparΔ9_3	IV-WRNVLAFVYLHV-GLLYGFYLILTG--RVKLW-TPVFAVTFTVLSALGVTAGAHRLWAHRAYKARWPL--

parallela		SFWG5A		RLILALLQTMAFQNHIYEWVRDHRVHHKFTET---DAD-PHNAKRGFFFSHIG---WLMVRK-----HKEVFEKGASIDMS--<br>DLEKDPIVMLQKKTYLVVMPILCFLLPSWIPVYFWGEDPWTSWYVASIWRYTMSLNFTWLVNSAAHIWGNKPFDKNI**[GATD]**NLTVAICA<br>IGEGWHNYHHVFPW--DYKAAELG-NYRTNISTAIIDLAAKYGWAYDLKTVSTQMIL
Choristoneura parallela	AAQ12889.1	Z9-desaturase SFWG4A	CparΔ9_4	IV-WRNVILFLLLHT-GAVYGGYLFFT---KAMWA-TKFFAIFLYLCSGLGITAGAHRLWAHKSYKARLPL--<br>RILLTLFNTMAFQDSVLDWARDHRMHHKYSET---DAD-PHNATRGFFFSHVG---WLLVRK-----HPQIKAKGHTIDMS--<br>DLCSDPVLRFQKKYYLTLMPIFCFILPTYIPT-<br>LWGESLWNAYFVAAIFRYCYVLNVTWLVNSAAHKWGDRPYDKNI**[NPVE]**TKPVSLVVFGEGFHNYHHVFPW--DYKTAELG-<br>GYSLNITKLFIDTMAKIGWAYDLKSVSPDIVE
Choristoneura parallela	ADC53487.1	desaturase Z9-III, partial	CparΔ9_5	--WPL--RLILALLQTMAFQNHIYEWVRDHRVHHKFTET---DAD-PHNAKRGFFFSHIG-<br>--WLMVRK-----HKEVFEKGASIDMS--<br>DLEKDPIVMLQKKTYLVVMPILCFLLPSWIPVYFWGEDPWTSWYVASIWRYTMSLNFTWLVNSAAHIWGNKPFDKNI**[GATD]**NLTVAICA<br>IGEGWHNYHHVFPW--DYKAAELG-NYRTNISTAIIDLAAKYGWAYDLKTVSTQMIL
Choristoneura rosaceana	AAN41250.1	acyl-CoA Z/E11 desaturase	CrosΔ11_1	II-YTNLLTFGYWHI-AGLYGLYLCFT---SAKWQ-TIILALILNEMAILGITAGAHRLWAHRSYKATVPL--<br>QIILIIFNSLSFQNSAIHWIRDHRMHHKYSDT---DGD-PHNASRGFFYSHVG---WLLVKK-----HPEVKKRAKTIDMS--<br>DIYSNPILRFQKKYAIPFIGMICFVLPTIIPMYFWGETLSNAWHIT-<br>MLRYVFSLNSIFLVNSAAHLYGYRPYDKNI**[LPAE]**NKMTFIACLGENFHNYHHVFPW--DYRASELG-<br>NIGMNWTAKFIDFFAWIGWAYDLKTASDENIK
Choristoneura rosaceana	ADC53485.1	desaturase Z/E11, partial	CrosΔ11_2	II-YTNLLTFGYWHI-AGLYGLYLCFT---SAKWQ-TIILALILNEMAILGITAGAHRLWAHRSYKATVPL--<br>QIILIIFNSLSFQNSAIHWIRDHRMHHKYSDT---DGD-PHNASRGFFYSHVG---WLLVKK-----HPEVKKRAKTIDMS--<br>DIYSNPILRFQKKYAIPFIGMICFVLPTIIPMYFWGETLSNAWHIT-<br>MLRYVFSLNSIFLVNSAAHLYGYRPYDKNI**[LPAE]**NKMTFIACLGENFHNYHHVFPW--DYRASELG-<br>NIGMNWTAKFIDFFAWIGWAYDLKTASDENIK
Choristoneura rosaceana	AAN39697.1	Z9-desaturase	CrosΔ9_1	YV-WRNIILFAYLHL-AAIYGGYLFLF---SAKWQ-TDIFAYFLYVASGLGITAGAHRLWAHKSYKAKWPL--<br>RLILTIFNTIAFQDSAIDWARDHRMHHKYSET---DAD-PHNATRGFFFSHIG---WLLVRK-----HPELKRKGKGLDLS--<br>DLYSDPILRFQKKYYMILMPLACFILPTVIPVYMWNETWSNAFFVAALFRYTFILNVTWLVNSAAHKWGDKPYDKSI**[KPSE]**NMSVSLFAF<br>GEGFHNYHHTFPW--DYKTAELG-NHRLNFTTKFINFFAKIGWAYDMKTVSQEIVQ
Danaus plexippus	EHJ69993.1	acyl-CoA delta-14 desaturase	DpleΔ14	VV-WKNAIGFFILHL-LGIWGHLIVFTG--GIYWQ-TFMWTSLLLFTSTEGITIGAHRLWSHRTFKATPLL--<br>KTILMIFQTLAGQNSIFTWCRDHRLHHRYSDT---DAD-PHNAKRGFFFSHIG---WLLCKK-----HPYVKELGKRIDMS--<br>DLQNDWMIMAQKKYYYYLYLIFAVIIPVSVPYYYFGESLKNSLLVCYFARYVFQLNGTWLVNSAAHLYGTRPYDKKL**[QPVE]**SWFVSFIS<br>FGEGWHNYHHAFPW--DYKAAELS--MHFNQSAKFIRIFEKLGLAYDLKTASPEMVQ
Danaus plexippus	EHJ68613.1	acyl-CoA delta-9 desaturase isoform	DpleΔ9	YV-WRNIIAFAYLHI-AALYGGYLFLF---SAKWQ-TNIFAYILYVMSGLGITAGAHRLWAHKSYKAKWPL--<br>RVILVIFNTLAFQDSAIDWARDHRMHHKYSET---DAD-PHNATRGFFFSHIG---WLLVRK-----HPELKRKGKGLDLS--<br>DLYADPILRFQKKYYLLLMPISCFILPTIIPVYLWGETWTNGYFVAAMFRYAFILNVTWLVNSAAHKWGDKPYDKNI**[QPSE]**NMSVSLFAL

				GEGFHNYHHTFPW--DYKTAELG-NNRLNFTTTFINFFAKIGWAYDLKTVSDEIVK
Epiphyas postvittana	AAL11496.1	acyl-CoA E11-desaturase	EposΔ11	IL-YTNLLIFGYGHL-AGLYGLYLCFT---SARLQ-TIILAFILHAMAILGITAGAHRLWTHRSYKATMPL--QIILIIFNSLSFQNSAINWVRDHRSHHKYCDT---DAD-PHNAARGLFYSHIG---WLLVKK-----HPEVKKRGKMTDMS--DVYRNPVLRFQKKYAVPFIGTICFVLPTIIPMYFWGESLNNAWHIT-LLRYIFSMHTIFLVNSVAHLWGNRPYDKNI**[LPAD]**NRTLSIATLGEASHNYHHTFPW--DYRSTELG-YLPTNFTTNFIDFFAWIGWAYDLKTTSGEIIN
Epiphyas postvittana	AAK94070.1	AF402775_1 acyl-CoA delta-9 desaturase	EposΔ9_1	IV-WRNVILFVLLHT-GAVYGGYLFFT---KAMWA-TKFFAFFLYLCSGLGITAGAHRLWAHKSYKARLPL--RILLTLFNTIAFQDSVLDWARDHRMHHKYSET---DAD-PHNATRGFFFSHVG---WLLVRK-----HPQIKAKGHTIDMS--DLCSDPVLRFQKKYYLTLMPLFCFILPTYIPT-LWGESLWNAYFVAAIFRYCYVLNVTWLVNSAAHKWGDRPYDKNI**[NPVE]**TKPVSLVVFGEGFHNYHHTFPW--DYKTAELG-GYSLNISKLFIDTMAKIGWAYDLKSVSPDIVE
Epiphyas postvittana	AAL35750.1	acyl-CoA delta-9 desaturase	EposΔ9_2	YV-WRNIILFAYLHI-AAVYGGYLFLF---SAKWQ-TDIFAYLLYVASGLGITAGAHRLWAHKSYKAKWPL--RLILTIFNTTAFQDSAIDWARDHRMHHKYSET---DAD-PHNATRGFFFSHIG---WLLVRK-----HPELKRKGKGLDLS--DLYADPILRFQKKYYLILMPLACFILPTVIPVYLWNETWSNAFFVAALFRYTFILNVTWLVNSAAHKWGDKPYDKSI**[KPSE]**NLSVSLFAFGEGFHNYHHTFPW--DYKTAELG-NHRLNFTTKFINFFAKIGWAYDMKTVSHEIVQ
Helicoverpa zea	AAF81787.1	AF272342_1 acyl-CoA delta-11 desaturase	HzeaΔ11	IV-YPNLITFGYWHI-AGLYGLYLCFT---SAKWA-TILFSYILFVLAEIGITAGAHRLWAHKTYKAKLPL--EILLMVFNSIAFQNSAIDWVRDHRLHHKYSDT---DAD-PHNASRGFFYSHVG---WLLVRK-----HPEVKKRGKELNMS--DIYNNPVLRFQKKYAIPFIGAVCFALPTMIPVYFWGETWSNAWHIT-MLRYIMNLNVTFLVNSAAHIWGNKPYDAKI**[LPAQ]**NVAVSVATGGEGFHNYHHVFPW--DYRAAELG-NNSLNLTTKFIDLFAAIGWAYDLKTVSEDMIK
Helicoverpa zea	AAF81788.1	AF272343_1 acyl-CoA delta-9 desaturase	HzeaΔ9_1	LV-WRNILLFAYLHL-AALYGGYLFLF---SAKWQ-TDIFAYILYVISGLGITAGAHRLWAHKSYKAKWPL--RVILVIFNTVAFQDAAMDWARDHRMHHKYSET---DAD-PHNATRGFFFSHIG---WLLVRK-----HPDLKEKGKGLDMS--DLLADPILRFQKKYYLILMPLACFVMPTVIPVYFWGETWTNAFFVAAMFRYAFILNVTWLVNSAAHKWGDKPYDKSI**[KPSE]**NLSVAMFALGEGFHNYHHTFPW--DYKTAELG-NNKLNFTTTFINFFAKIGWAYDLKTVSDDIVK
Helicoverpa zea	AAF81790.2	AF272345_1 acyl-CoA delta-9 desaturase	HzeaΔ9_2	IV-WRNVILMGMLHI-GGVYGAYLFLT---TAMWR-TCIFAVVLYICSGLGITAGAHRLWAHKSYKARLPL--RLMLTLFNTLAFQDAVIDWARDHRMHHKYSET---DAD-PHNATRGFFFAHVG---WLLVRK-----HPQIKAKGHTIDLS--DLKSDPILRFQKKYYLFLMPLVCFILPCYIPT-LWGESLWNAYFVCSIFRYVYVLNVTWLVNSAAHLWGAKPYDKNI**[NPVE]**TRPVSLVVLGEGFHNYHHTFPW--DYKTAELG-DYSLNLTKLFIDTMAAIGWAYDLKTVSTDVIQ
Mamestra brassicae	ABX90049.1	acyl-CoA delta 11 desaturase	MbraΔ11	IV-YPNLFTFGYWHI-AGLYGLYLCFT---SAKWQ-TMIFSFILFVLAEIGVTAGAHRLWAHKTYKAKLPL--QILLMILNSIAFQNSAIDWVRDHRLHHKFSDT---DAD-PHNATRGFFYSHVG----WLLVRK-----HPEVKRRGKELDMS--DIYNNPVLRFQKNYAIPFIGAVCFGLPTLIPVYCWGETWSTAWHIT-MFRYVMNLNVTFLVNSAAHIWGKKPYDKKI**[LPAQ]**NIAVSIATCGEGFHNYHHVFPW--DYRAAELG-

				NNSLNVTTKFIDFFAWIGWAYDLKTVSQDMIK
Mamestra brassicae	ABX90048.1	acyl-CoA delta 9 desaturase	MbraΔ9	YV-WRNIILFAYLHL-AALYGGYLFLF---SAKWQ-TDVFAYILYVMSGLGITAGAHRLWAHKSYKAKWPL--<br>KVILIIFNTIAFQDAAMDWARDHRMHHKYSET---DAD-PHNATRGFFFSHIG---WLLVRK-----HPDLKKKGKGLDMS--<br>DLLNDPILKFQKKYYLLLMPLACFVMPTMIPVYLWGETWTNAFFVAAMFRYAFILNVTWLVNSAAHKWGDKPYDKSI**[KPSE]**NLSVAMF<br>ALGEGFHNYHHTFPW--DYKTAELG-NQKLNFTTTFINFFAKLGWAYDMKTVSDDIVK
Manduca sexta	ALA65425.1	Z11-fatty acid desaturase	MsexΔ11	II-YTNIITFTYWHL-AGLYGLYLCFT---TAKWA-TIIMSWLIFVSAAIGVTAGAHRLWAHRTYKAKLPL--<br>QILLMVFNTFAFQNTAINWVRDHRLHHKFSDT---DAD-PHNATRGFFYSHVG---WLLVKK-----HPEVKRRGKLIDMS--<br>DIYSNKVLVFQKNYAIPFIGMVCFILPTLIPVYCWGESLNTAWHIT-<br>MLRYVANLNITFLVNSAAHLWGNKPYDKNI**[LAAQ]**NLSVSFAAFGEGFHNYHHVFPW--DYRTAELG-<br>NNYLNLSTKFIDFFAWLGWAYDLKAVPVGMAK
Manduca sexta	CAJ27975.1	acyl-CoA delta-9 desaturase	MsexΔ9	LV-WRNIILFAYLHI-AALYGGYLFLV---HAKWQ-TDIFAYLLYVMSGLGITAGAHRLWAHKSYKAKWPL--<br>RLILVVFNTMAFQDSAIDWARDHRMHHKYSET---DAD-PHNATRGFFFSHIG---WLLVRK-----HPELKRKGKGLDLS--<br>DLYADPILRFQKKYYLILMPITCFVMPTVIPVYLWGESWVNAFFVAALFRYAFILNVTWLVNSAAHKWGDKPYDKSI**[KPSE]**NISVSMFAL<br>GEGFHNYHHTFPW--DYKTAELG-NNRLNFTTNFINFFAKIGWAYDLKTVSDEIIQ
Operophtera brumata	KOB63425.1	Delta 11 desaturase	ObruΔ11_1	II-KTNLVTFGYGHL-AALYGLYLGVT---SAHWG-TLLLAYIIFVAAAIGVTAGAHRLWAHRTYKATLPL--QILLMIMNTFAFQNTAI------------<br>TDT---EAD-PHNATRGFFYSHMG---WLLVKK-----NEH---TLSTAWHIT-<br>VLRYILNLHITFLVNSAAHFWGNKPYDKSI**[RPVQ]**SLPVSFVAFGEGFHNYHHVFPW--DYKAAELG-<br>NNRLNVSTKFIDFFAWLGWAYDLKSVPDEMVR
Operophtera brumata	KOB65151.1	Delta11-desaturase	ObruΔ11_2	YI-PKHVILFLYVHL-SGLYGLYLALT---AARWE-TIGLTIVLNYASIVGITAGAHRLWSHRAYKAKLPL--<br>QILLAAMTSLAFQFSTITWVRDHRAHQKFSDT---DAD-PHNSKKGLFFSHMG---WLMMDK-----NPAIKRKAKGIDLS--<br>DVYNNPVLRFQHDNFIIVGGLACFVVPPLIPL-LWGESLWVAWHMN-<br>LARYILSLHPIFLVNSAAHKWGNKPYDRSI**[APSQ]**NIGVSIANLGEGFHNYHHTFPF--DYRAAELG--<br>NIFNPTTKFIDLFAWLGWAYDLKTASHDLVT
Operophtera brumata	KOB75262.1	Delta-9 desaturase 14-26, partial	ObruΔ9	---MF-----------TGRCRMWTILF--------------------------AGAHRLWAHRAYKARWPL--RLFLAVMQTMAFQNHIYEWVRDHRVHHKFTET---<br>DAD-PHNAKRGFFFSHIG---WLMLRK-----HKDVFDKGATVDMS--<br>DLEKDPIVMFQKRTYLVLMPLLCFVFPSMIPVYFWGEDAWTSWYVASITRYTVSLHFTWLVNSAAHIWGNRPYDKNI**[GATD]**NVSVAICA<br>FGEGWHNYHHVFPW--DYKAAELG-NYSTNLSTALIDFAAK----------HAEMIK
Ostrinia furnacalis	AAL32060.1	AF441861_1 acyl-CoA Z/E11 desaturase	OfurΔ11	IL-YFNVMTFTFLHL-SALYGLYLGFT---SVKWA-TIGLGIIFYFFAEIGITAGAHRLWSHRSYKAKLPL--<br>EILLMVFNSMAFQNTALSWARDHRVHHKCPDT---NGD-PHNANRGFFYSHVG---WLMTKK-----SDEVIKQGKLCDVA--<br>DLYSNPVLRFQKKYAVPFIGTLCFVLPTLIPMYFWGETLNNAWHFN-<br>MFRYVINLNATFCVNSVVHKWGYKPYDKNI**[CPTQ]**NVLLNLAVLGEAFHNYHHVFPW--DYRAAELG-<br>NQKMNPTTLFIDFFAWIGWAYDLKTASKEMIK
Ostrinia furnacalis	AAL35746.2	acyl-CoA delta-14	OfurΔ14	II-WPIVMVYVVMHI-GAITGLLLVLGG--NVKIA-SIIWAVFYSLVATEGAHMGAHRCFSHRAFKAKPLL--

		desaturase			KVILLIMQTISGQHSTYIWCRDHRQHHRYSDT---DGD-PHNSKRGMFYCHVG---WLMTSR-----HPLCKKLRKTIDMS--DLQQDPLVMFQYRYFRSLFFTFGFLLPVWVPMHFFQESFTNAVFVCFFLRYVYALHVTYFINSLAHKYGTRPYDKTI**[QPVE]**TWFVSLLSLGEGWHNYHHAYPW--DYKAAEIG--MPLNSTASLIRLCASLGLAYDLKSVDPETLN
Ostrinia furnacalis	AAL27033.1	acyl-CoA desaturase	delta-9	OfurΔ9_1	LV-WRNIILFAYLHL-AAVYGAYLFLF---SAKWQ-TDIFAYILYVISGLGITAGAHRLWAHKSYKAKWPL--RLILIIFNTVSFQDSALDWSRDHRMHHKYSET---DAD-PHNATRGFFFSHIG---WLLVRK-----HPELKRKGKGLDLS--DLYADPILRFQKKYYLLLMPLGCFIMPTVVPVYFWGETWTNAFFVAALFRYTFILNVTWLVNSAAHKWGHKPYDSSI**[KPSE]**NLSVSLFALGEGFHNYHHTFPW--DYKTAELG-NNRLNFTTNFINFFAKIGWAYDLKTVSDEIIQ
Ostrinia furnacalis	AAL27034.1	acyl-CoA desaturase	delta-9	OfurΔ9_2	IV-WRNVAIFTYLHL-GFLYGAYLMLT---TVMWK-TRLFCLILYVCSGLGITAGAHRLWAHKSYKAKLPL--RIILTLFNTLAFQDAVVDWARDHRMHHKYSET---DAD-PHNATRGFFFSHVG---WLLVRK-----HPQIKAKGHTIDMS--DLRADPVLRFQKKYYMYLMPLICFIMPSVVPA-LWGETVWNGYFTCAVFRYVAVLNGTWLVNSWAHLWGDKPYDRHI**[NPVE]**TKVVSVAAIGEGFHNYHHTFPW--DYKAAELG-NYTFNITKFFIDTMATIGWAYDLKTVSTDVIQ
Ostrinia nubilalis	ACA81687.1	acyl-CoA desaturase	delta-11	OnubΔ11_1	IL-YFNVMTFTFLHL-SALYGLYLGFT---SVKWA-TIGLGIIFYFFAEIGITAGAHRLWSHRSYKAKLPL--EILLIVFNSMAFQNTALSWARDHRVHHKCPDT---NGD-PHNANRGFFYSHVG---WLLTKK-----SDEVIKQGKLCDVA--DLYSNPVLRFQKKYAVPFIGMLCFVLPTLIPMYFWGETLNNAWHFN-MFRYVINLNATFCVNSVVHKWGYKPYDKNI**[CPTQ]**NVLLNLAVLGEAFHNYHHVFPW--DYRAAELG-NQKMNPTTLFIDFFAWIGWAYDLKTASKEMIK
Ostrinia nubilalis	ADB25212.1	desaturase beta	ezi-D11	OnubΔ11_2	II-YRYVISFSYLHL-AALYGLYLCLT---SVKWP-TIGFGLLMYHITIIGITAGAHSLWSHRSYKAKFPL--QVILMVLNSMAFQNTTITWARDHRVHHKCSDT---NGD-PHNANRGFFFSHMG---WLMTKK-----SDEVIKQGKSMDMS--DLYNNPVLRFQRKYALPVIGTLCFILPTFIPMYFWGETLNNAWHMN-ILRYVTVLHVTCCINSVAHKWGYKPYDKNI**[LPSQ]**NVLLSTFALGEGFHNYHHVFPS--DYQASEFG-NKKLNLTTWFIDFFAKIGWAYDMKTASEEVVR
Ostrinia nubilalis	ADE58523.1	desaturase beta	ezi-D11	OnubΔ11_3	II-YRYVISFSYLHL-AALYGLYLCLT---SVKWP-TIGFGLLMYHITIIGITAGAHRLWSHRSYKAKFPL--QVILIVLNSMAFQNTTLTWARDHRVHHKCSDT---NGD-PHNANRGFFFSHMG---WLMTKK-----SDEVIKQGKSMDMS--DLYNNPVLRFQRKYALPVIGTLCFILPTFIPMYFWGETLNNAWHMN-ILRYVTALHVTCCVNSVAHNPFNKPYDKNI**[LPSQ]**IVLLSTFGLGEGFHNYHHVFPS--DYQASELG-NKKLNLTTWFIDFFAKIGWAYDMKTASEEVVR
Ostrinia nubilalis	ADE58524.1	desaturase beta, partial	ezi-D11	OnubΔ11_4	II-YRYVISFSYLHL-AALYGLYLCLT---SVKWP-TIGFGLLMYHITIIGITAGAHRLWSHRSYKAKFPL--QVILIVLNSMAFQNTTLTWARDHRVHHKCSDT---NGD-PNNANGGFSLSHMG---WLMTRK-----SDEVIKQGKSMDMS--DLYNNPVSRFQRKYALPVIGTLCFIHPTFIPMYFWGETLNNAWHMN-ILRYVTVLHVTCCINSVAHKWGYEPYDKNI**[LPSQ]**NILLSTFALGEGFHNYHHVFPW--DYQAAEFG-NKKLSLTTLFIDFFAKIGW-------------
Ostrinia nubilalis	ADE58525.1	desaturase	ezi-D11	OnubΔ11_5	---MYHITIIGITAGAHRLWSHRSYKAKFPL--QVILIVLNSMAFQNTTLTWARDHRVHHKCSDT---NGD-

		beta, partial		PHNANRGFFFSHMG---WLMTKK-----SDE--
Ostrinia nubilalis	ADE58526.1	desaturase ezi-D11 beta	OnubΔ11_6	II-YQYVISFSYLHL-AALYGLYLCLT---SVKWP-TIGFGLLMYHITIIGITAGAHRLWSHRSYKAKFPL--QVILIVLNSMAFQNTTLTWARDHRVHHKCSDT---NGD-PHNANRGFFFSHMG---WLMTKK-----SDEVIKQGKSMDMS--DLYNNPVLRFQRKYALPVIGTLCFILPTFIPMYFWGETLNNAWHMN-ILRYVTVLHVTCCINSVAHKWGYKPYDKNI**[LPSQ]**NVLLSTFALGEGFHNYHHVFPS--DYQASEFG-NKKLNLTTWFIDFFAKIGWAYDMKTASEEVVR
Ostrinia nubilalis	ADE58531.1	desaturase ezi-D11 alpha	OnubΔ11_7	---MLTYQLSILGITAAAHRLWSHRSYKAKFPL--QVILMVLNCMSFQNSALNWCRDHRVHHKCSDT---DGD-PHNASRGFFFSHIG---WLMTKK-----SEEVKRQGKSIDMS--DLYNNPVLRFQKKYAVPLIGTICFILPTFIPLYFWGETLNNAWHMN-ILRYVLSLHATWCVNSVAHKWGYKPYDKNI**[LPSQ]**NILLSTAMLGEGFHNYHHVFPW--DYQAAELG-NKKFNHTTYFIDFFAWVGWAYDLKSSSEEVVR
Ostrinia nubilalis	ADE97412.1	acyl-CoA delta-11 desaturase, partial	OnubΔ11_8	IL-YFNVMTFTFLHL-SALYGLYLGFT---SVKWA-TIGLGIIFYFFAEIGITAGAHRLWSHRSYKAKLPL--EILLIVFNSMAFQNTALSWARDHRVHHKCPDT---NGD-PHNANRGFFYSHVG---WLLTKK-----SDEVIKQGKLCDVA--DLYSNPVLRFQKKYAVPFIGMLCFVLPTLIPMYFWGETLNNAWHFN-MFRYVINLNATFCVNSVVHKWGYKPYDKNI**[CPTQ]**NVLLNLAVLGEAFHNYHHVFPW--DYRAAELG-NQKMNPTTLFID--------------------
Ostrinia nubilalis	AAL35330.1	AF441220_1 acyl-CoA Z/E14 desaturase	OnubΔ14	II-WPIVMVYVVMHI-GAITGLLLVLGG--NVKIA-SIIWAVFYSLVATEGAHMGAHRCFSHRAFKAKPLL--KVILLIMQTISGQHSTYIWCRDHRQHHRYSDT---DGD-PHNSKRGMFYCHVG---WLMTSR-----HPLCKKLRKTIDMS--DLQQDPLVMFQYRYFRSLFFTFGFLLPVWVPMHFFQESFTNAVFVCFFLRYVYALHVTYFINSLAHKYGTRPYDKTI**[QPVE]**TWFVSLLSLGEGWHNYHHAYPW--DYKAAEIG--MPLNSTASLIRLCASLGLAYDLKSVDPETLN
Ostrinia nubilalis	ADE58520.1	acyl-CoA desaturase Z9-1	OnubΔ9_1	LV-WRNIILFAYLHL-AAVYGAYLFLF---SAKWQ-TDIFAYILYVISGLGITAGAHRLWAHKSYKAKWPL--RLILIIFNTVSFQDSALDWSRDHRMHHKYSET---DAD-PHNATRGFFFSHIG---WLLVRK-----HPELKRKGKGLDLS--DLYADPILRFQKKYYLLLMPLGCFIMPTVVPVYFWGETWTNAFFVAALFRYTFILNVTWLVNSAAHKWGHKPYDSSI**[KPSE]**NLSVSLFALGEGFHNYHHTFPW--DYKTAELG-NNRLNFTTNFINFFAKIGWAYDLKTVSDEIIQ
Ostrinia nubilalis	ADE58521.1	acyl-CoA desaturase Z9-1, partial	OnubΔ9_2	LV-WRNIILFAYLHL-AAVYGAYLFLF---SAKWQ-TDIFAYILYVISGLGITAGAHRLWAHKSYKAKWPL--RLILIIFNTVSFQDSALDWSRDHRMHHKYSET---DAD-PHNATRGFFFSHIG---WLLVRK-----HPELKRKGKGLDLS--DLYADPILRFQKK--
Papilio machaon	KPJ08485.1	Acyl-CoA Delta(11) desaturase	PmacΔ11_1	YV-WFNIFWFLFLHI-SSAYGLYLAFT---SAKWQ-TNVFAFAIHLMCAIGIGAGSHRLWTHRCFKAKTPL--RIVLMIWQTMGFQDSIFEWARDHRTHHKYADT---DGD-PHNAERGLFFSHMG---WLCCKK-----SPEVIEGGKRIDLS--DLYEDPVVMFQKKHYMKMMPILCFVLPTILPVYLWGETWTNAFFIPTILRYTFGINVVWSVNSFAHKFGYRPYDKSL**[NPRE]**NIAVWMFCV-EGFHNYHHTFPW--DYRASEHPLINMLTPTIVFIEAMAKIGQAYDLKAVSPEIIK
Papilio machaon	KPJ11142.1	Acyl-CoA Delta(11) desaturase	PmacΔ11_2	IR-WTSAVPIVIFHLITALSIVYAVIVG-SIPKWQ-TFLFGYFMGQVAGFGVTAGAHRYWIHRSYKATFPL--QMILIICYSVAGQNNIYNWVRDHRIHHKFSET---SAD-PHDARRGFFFSHVG---WLMMKK-----HPAVIREGRKINIR--DIANDPLVQFHTKYFDVFKFVFCFFLPTLIPVYAWQETWTNAILSQPILRYALSLNFTWSVNSFAHIWGNKPYDRHI**[SPAE]**NWGVSAVAMG

				EGWHNYHHTFPW--DYKASELA--YINNITTSLLNLFAKIGWAYDLKQASPSLIK
Papilio machaon	KPJ12039.1	Acyl-CoA Delta(11) desaturase	PmacΔ11_3	IV-YSNILKYTVLHV-FAFYGLYVVFA---KAKWQ-TFAFNYVTTHLSAFGVTVGAHRLWAHKAYKATLPM--QVVLMLLNSLAFQSTAFEWIRDHRLHHKYSDT---DAD-PYNASRGFFFSHIG---WLLVRK-----HPLVLKKGKTIDMS--DIYNNPVLKFQQKYAIIVIGLCCYILPTLIPMYFWSETLTNSFFTN-ILRHVITLHATFSVNSVAHLFGTKPYDKNI**[KPVQ]**SLFVSFASNGEGYHNYHHVFPY--DYRAAEFG--GWLNISKAVIDILAKFGLVYDLKMASESVIT
Papilio machaon	KPJ12046.1	Acyl-CoA Delta(11) desaturase	PmacΔ11_4	IV-YLRVVQYTVLHI-FAAYGLYLGIT---EVKWR-TVISFCIFLLASIVGITVAAHRLWSHKAFKATMPL--QVALMLCNSIAFQSTAIDWIRDHRLHHKHSDT---DAD-PYNASRGFFFSHIG---WLLVRK-----HPLVLNKGKTVDMS--DIYNNPVLKFQQKHAVVVIGLCCYVIPTIIPMYFWNETFSNAFFTN-VLRHVIGLHLAFSVNSFAHLWGTKPYDKSI**[LPVQ]**SLFVSLISGGEGFHNYHHVFPC--DYRTAEIG-NNWLNPSTLFIDVLAKFGLAYDLKFTPENVIT
Papilio machaon	KPJ16137.1	Acyl-CoA Delta(11) desaturase	PmacΔ11_5	IV-YRNILTFGYAHL-TALYGFYLACT---EATWS-TIIFSYILFALAAIGITAGAHRLWTHRAYKAKLPL--QIILVVLNSLAFQNTAIDWVRDHRLHHRYSDT---DAD-PHNATRGFFYSHVG---WLLVRK-----HEEVKRRGKFIDMS--DIYANPVLRFQKKYAIPFIGTICFVLPTIIPMYFFGESLKTAWCIA-VLRYVLNLHIAFLVNSAAHLWGNKPYDRTI**[KPVQ]**SLPVSFVAFGEGFHNYHHVFPW--DYRTAELG-NNYLNLTTIFIDFFAKIGWAYDLKSVTDDMIQ
Papilio machaon	KPJ16138.1	Acyl-CoA Delta(11) desaturase	PmacΔ11_6	IV-YVNVFIYAAYHL-FAIYGLYLSFT---SVKWP-TIILGVILWTLSVLGTTMGSHRLWSHRAYKAKLPL--QILLMICTSIACQLTSYNWVRDHRMHHKYSDT---NAD-PHNAKRGFFFSHMG---WLFIKK-----HSEMKRLGNTIYMD--DVHSNPVLMFQKKYAIPFMGSLCFIIPVLIPMYFWNETFVNAWNFN-ILRFILGVHSICSINSFAHLWGSRPYDENI**[MPVE]**NMGVSLVTLGEGFHNYHHSFPW--DYKAGELG-NNWLNLSTSTIDFFAKLGWAYDLKTASGEVVE
Papilio machaon	KPJ16371.1	Acyl-CoA Delta(11) desaturase	PmacΔ11_7	IK-WSTAIPIILYHV-LAVYWCYHFAF---PVKWQ-TVLYAVIMYILTGFGITGGAHRLWTHKSYKATLPL--KLFYLVCFAAAGQNSIYQWVRDHRVHHKYSDT---EAD-PHNANRGLFFSHIG---WLMMKK-----NSQVQQRGKEMDMS--DIEADPILRFYNKYFNYFKLMFCYILPTTLGVWLWGEEWKCAVAWQCFIRFLIMFHSELTVNSLAHAYGYRPYNKNI**[IPRE]**NRFVATCTLGEGWHNYHHAFPF--DYKAAEH--FDAFNLCTTFINGFKWLGWAYDLREATPQMIN
Papilio machaon	XP_014356697.1	PREDICTED: acyl-CoA Delta(11) desaturase-like	PmacΔ11_8	IV-YVNVFIYAAYHL-FAIYGLYLSFT---SVKWP-TIILGVILWTLSILGITMGSHRLWSHRAYKAKLPL--QILLMICTSIACHLTSYNWIRDHRMHHKYSDT---NAD-PHNAKRGFFFSHIG---WLFIKK-----HSEMKRLGNTIYMD--DVRSNPVLMFQKKYAIPFMGSLCFIIPVLIPMYFWNETFVNAFNFN-ILRFILGVHAICSVNSFAHLWGSRPYDESI**[MPVE]**NMGVSLVTLGEGFHNYHHSFPW--DYKAGELG-NNWLNFSTSAINFFAKLGWAYDLKTASGEVVE
Papilio machaon	KPJ18423.1	Sphingolipid delta(4)-desaturase DES1	PmacΔ4	LYGYDPLFKWVVTAM-VLMQLLMLPLVK--YMSWPVLLLVAYCFGGVINHSLMLAIHEIAHNLAFGHNRPLHNRLFGFFANLPIGVPVSISFKKYHLEHHRYQGDEVIDVDLPTLLEAKLFCTTGGKLVWLFLQPFFYALRPLVVRPKPPTPLE-----------------MINLVIQLFFDAVVVELFGL-

				RALWYLLLGSFMAMGIHPVAGHFVSEHYMFRKGFETYSYY------GPLNWITFNVGYHNEHHDFPAVPGRRLPEVK-RIASEFYDDLPHHTSWSSVLYDFVMDPDIGPY
Papilio polytes	XP_013136853.1	PREDICTED: acyl-CoA Delta(11) desaturase-like	PpolΔ11_1	YV-WFNIFWFLFLHI-SSAYGLYLAFT---SAKWQ-TNVFAFAIHLMCAIGIGAGSHRLWTHRCFKAKTPL--RTVLMIWQTMGFQDSIFEWARDHRTHHKYADT---DGD-PHNAERGLFFSHMG---WLCCKK-----SPEVIEGGKRIDLS--DLYEDPVVMFQKKHYLKMMPILCFVLPTVFPVLLWGESWTNAFFIPTILRYTFGINVVWSVNSFAHKFGYRPYDKSL**[NPRE]**NIAVWMFCV-EGFHNYHHTFPW--DYRASEHPLINMLTPTIVFIEAMAKIGQAYDLKAVSPEIIK
Papilio polytes	XP_013139284.1	PREDICTED: acyl-CoA Delta(11) desaturase-like	PpolΔ11_2	IV-YRNIVTFGYAHL-TALYGLYLACT---EATWA-TIIFSYVLFALAAIGVTAGAHRLWTHRAYKAKLPL--QIILIVINSLAFQNTAIDWVRDHRLHHRYSDT---DAD-PHNATRGFFYSHMG---WLLVRK-----HEEVKRRGKFIDMS--DIYANPVLQFQRKYAIPFIGTICFVLPTVIPMYFFGESLKTAWCIA-ILRYVLNLHIAFLVNSAAHLWGNKPYDRTI**[KPVQ]**SLPVSFVAFGEGFHNYHHVFPW--DYRTAELG-NNYLNLTTIFIDFFAKIGWAYDMKSVTDDMIK
Papilio polytes	XP_013139285.1	PREDICTED: acyl-CoA Delta(11) desaturase-like	PpolΔ11_3	IV-FSNIFKYTVLHI-FAFYGIYVVLT---KAKWQ-TFVFNYITTYLSAFGVTIGAHRLWAHKSFKATMPV--QIVLMLMNSLAFQSTAFVWIRDHRLHHKYSDT---DAD-PYNASRGFFFSHIG---WLLVRK-----HPLVLKKGKTVDMS--DIYNNPVLKFQQKYAIVVIGLCCYIIPTYIPIYLWNETFSNSFYAN-ILRHVITLHATFSVNSVAHLFGTKPYDKNI**[KAVQ]**TLFVAFISNGEGYHNYHHVFPW--DYRAAEFG--GWLNPSKWIIDVLAKYGLVYDLKIAPKRLIS
Papilio polytes	XP_013142761.1	PREDICTED: acyl-CoA Delta(11) desaturase-like	PpolΔ11_4	IV-YQNVLIYTAYHI-FAIYGLYLTFT---SVKWP-TIVLGVILWTLSILGTTMGSHRLWSHRAYKATLPL--QIFLMICTSIACQLTSYNWVRDHRMHHKYSDT---NAD-PHNAKRGFFFSHFG---WLLIKK-----HSEMKRLGETIYMD--DVHSNPVLMFQKKYAIPLMGSLCFVVPVLIPMYFWNETFVNAWNFN-ILRFILGVHSICSINSFAHLFGSRPYDESI**[MPVE]**NMGVSLVTLGEGFHNYHHSFPW--DYKAGELG-NHWLNFSTASIDFFAKIGWAYDLKTASGAVVE
Papilio polytes	XP_013142839.1	PREDICTED: acyl-CoA Delta(11) desaturase	PpolΔ11_5	IR-WTSAIPIVIFHLITVLSIAYSIIIGN-IPKWQ-TFLFGYIMGQVAGFGVTAGVHRYWTHRSYKATFPL--QIILVICYSVAGQNSIYNWVRDHRIHHKFSET---SAD-PHDARRGFFFSHVG---WLMMKK-----HPAVIREGRKIDMR--DIANDPLIQFHTKYFDVFKFFFCFFLPTLIPVYGWDETWTNAILSQPVLRYALSLNFTWSVNSFAHIWGNKPYDRHI**[SPAE]**NWGVSAVAMGEGWHNYHHTFPW--DYKASELA--YVNNITTSLLNLFAKIGWAYDLKQASPGLIR
Papilio polytes	XP_013143737.1	PREDICTED: sphingolipid delta(4)-desaturase DES1	PpolΔ4	LYGYDPLFKWVVLSM-VLMQLLVLPLVK--YMNWPVLLLVAYCFGGVINHSLMLAIHEIAHNLAFGHNRPLHNRLFGFFANLPIGVPVSISFKKYHLEHHRYQGDEVIDVDLPTLLEAKLFCTTGGKLFWLFLQPFFYALRPLVVRPKPPTPLE----------------MINLVIQLFFDAVVVELFGW-RALWYLLLGSFMAMGIHPVAGHFVSEHYMFRKGFETYSYY------GPLNWITFNVGYHNEHHDFPAVPGRRLPEVK-RIASEFYDDLPHHTSWSSVLYDFVMDPDIGPY
Papilio xuthus	KPI92675.1	Acyl-CoA Delta(11) desaturase	PxutΔ11_1	IV-YVNVFIYSAYHI-FALYGLYLSFT---SVKWP-TIILGIILWTLSILGTTMGSHRLWSHRAYKANLPL--QIFLMICTSIACQLTSYNWVRDHRMHHKYSDT---NAD-PHNAKRGFFFSHMG---WLLIKK-----HTEMKRLGNTIYMD--DVHSNPVLMFQKKYAIPFMGSLCFIIPVLIPIYFWNESFVNAWNFN-

				ILRFILGVHSICSINSFAHLFGTRPYDENI**[MPVE]**NIGVSLVTLGEGFHNYHHSFPW--DYKAGELG-<br>NNWLNFSTATIDFFAKLGWAYDLKTASDAVVE
Papilio xuthus	XP_013164773.1	PREDICTED: acyl-CoA Delta(11) desaturase-like	PxutΔ11_10	YV-WFNIFWFLFLHI-SSVYGLYLAFT---SAKWQ-TNVFAFAIHQMCAIGIGAGSHRLWTHRCFKAKTPL--<br>RTVLMIWQTMGFQDSIFEWARDHRTHHKYADT---DGD-PHNAERGLFFSHMG---WLCCKK-----SPEVIEGGKRIDLS--<br>DLYEDPVVMFQKKHYLKMMPILCFVLPTVLPVYLWGETWINAFFIPTILRYTFGINVVWSVNSFAHKFGYRPYDKSL**[NPRE]**NIAVWMFC<br>V-EGFHNYHHTFPW--DYRASEHPLINMLTPTIVFIEAMAKIGQAYDLKAVSPEIIK
Papilio xuthus	XP_013169713.1	PREDICTED: acyl-CoA Delta(11) desaturase-like	PxutΔ11_11	IK-WSTAILIILYHV-LAVYWCYHYAL---PVKWQ-TVLYATVMYILSGYGITGGAHRLWTHKSYKATLPL--<br>KLFYLVCFAAAGQNTLYQWVRDHRVHHKYSDT---EAD-PHNANRGLFFSHIG---WLMMKK-----NSQVKQRGKEMDMS--<br>DLEADPILRFYNKYFHYFKLMFCYILPTTLGVWLWGEEWKCAVAWQCFIRFLSMFHSELTVNSLAHAYGYKPYNKNI**[VPRE]**NRFVATCT<br>FGEGWHNYHHAFPF--DYKAAEH--FDAFNLCTTFINGFKWLGWAYDLREATPQMIN
Papilio xuthus	XP_013178722.1	PREDICTED: acyl-CoA Delta(11) desaturase-like	PxutΔ11_12	YV-WFNIFWFLFLHI-SSVYGLYLAFT---SAKWQ-TNVFAFAIHQMCAIGIGAGSHRLWTHRCFKAKTPL--<br>RTVLMIWQTMGFQDSIFEWARDHRTHHKYADT---DGD-PHNAERGLFFSHMG---WLCCKK-----SPEVIEGGKRIDLS--<br>DLYEDPVVMFQKKHYLKMMPILCFVLPTVLPVYLWGETWINAFFIPTILRYTFGINVVWSVNSFAHKFGYRPYDKSL**[NPRE]**NIAVWMFC<br>V-EGFHNYHHTFPW--DYRASEHPLINMLTPTIVFIEAMAKIGQAYDLKAVSPEIIK
Papilio xuthus	KPI94378.1	Acyl-CoA Delta(11) desaturase	PxutΔ11_2	IV-YRNILTFGYAHL-TALYGLYLACT---EATWS-TIIFSYILFALAAIGITAGAHRLWTHRAYKAKLPL--<br>QIILVVLNSLAFQNTAIDWVRDHRLHHRYSDT---DAD-PHNATRGFFYSHIG---WLLVRK-----HEEVKRRGKFIDMS--<br>DIYANPVLCFQRKYAIPFIGTICFVLPTIIPMYFFGESLKTAWCIA-<br>VLRYVLNLHIAFLVNSAAHIWGNKPYDRTI**[KPVQ]**SLPVSFVAFGEGFHNYHHVFPW--DYRTAELG-<br>NNYLNLTTIFIDFFAKIGWAYDLKSVNDDMIK
Papilio xuthus	KPJ02273.1	Acyl-CoA Delta(11) desaturase	PxutΔ11_3	IV-YLRVLQYTVLHI-FAAYGLYLGIT---AAKWK-TIISFCIFLIASVVGITVAAHRLWSHKAFKATMPL--<br>QIVLMLFNSIAFQSTAIDWIRDHRLHHKHSDT---DAD-PYNASRGFFFSHIG---WLLVRK-----HPLVLKKGKTVDMS--<br>DIYNNPVLKFQQKHAVIVIGLCCYVVPTIIPIYFWNETFSNAFYIN-<br>TLRHVIGLHLAFSVNSFAHLWGTKPYDKSI**[LPVQ]**SLFVSLISGGEGFHNYHHVFPC--DYRTAEIG-<br>NNWLNPSTLFIDVLAKFGLVYDLKSTPENVIN
Papilio xuthus	KPJ02274.1	Acyl-CoA Delta(11) desaturase	PxutΔ11_4	IV-YTNILKYTVLHV-FAFYGLYVTLT---KAKWK-TLIFHYVTTHLSAFGITVGAHRLWAHKAFKATLPM--<br>EVILMLLNSLAFQSTAFEWIRDHRLHHKYSDT---DAD-PYNASRGFFFSHIG---WLLVRK-----HPLVLKKGKTIDMS--<br>DIYNNPVLKFQQKYAIIVIGLCCYILPTIIPIYFWNETFYNSFHTN-<br>ILRHVITLHATFSVNSIAHLYGTKPYDNNI**[KAVQ]**SLIVTLVSNGEGYHNYHHVFPC--DYRAAEYG--<br>CWLNTSKFLIDILAKFGLVYDLKMASDSVIK
Papilio xuthus	KPJ02275.1	Acyl-CoA Delta(11) desaturase	PxutΔ11_5	IV-YSNILKYTVLHV-FAFYGLYVTLT---KAKWK-TLIFHYVTTHLSAFGITVGAHRLWAHKAFKATLPM--<br>EVVLMLLNSLAFQSTAFEWIRDHRLHHKYSDT---DAD-PYNASRGFFFSHIG---WLLVRK-----HPLVLKKGKTIDMS--<br>DIYNNPVLKFQQKYAIIVIGLCCYILPTIIPIYFWNETFYNSFHTN-<br>ILRHVITLHATFSVNSIAHLYGTKPYDNNI**[KAVQ]**SLIVTLVSNGEGYHNYHHVFPC--DYRAAEYG--

				CWLNTSKFLIDILAKFGLVYDLKMASDSVIK
Papilio xuthus	XP_013164010.1	PREDICTED: acyl-CoA Delta(11) desaturase-like	PxutΔ11_6	IV-YLRVLQYTVLHI-FAAYGLYLGIT---AAKWK-TIISFCIFLIASVVGITVAAHRLWSHKAFKATMPL--QIVLMLFNSIAFQSTAIDWIRDHRLHHKHSDT---DAD-PYNASRGFFFSHIG---WLLVRK-----HPLVLKKGKTVDMS--DIYNNPVLKFQQKHAVIVIGLCCYVVPTIIPVYFWNETFSNAFFIN-TLRHVIGLHLAFSVNSFAHLWGTKPYDKSI**[LPVQ]**SLFVSLISGGEGFHNYHHVFPC--DYRTAEIG-NNWLNPSTLFIDVLAKFGLVYDLKSTPENVIN
Papilio xuthus	XP_013164539.1	PREDICTED: acyl-CoA Delta(11) desaturase-like	PxutΔ11_7	IR-WTSAIPIVIFHLITALSIIYSVIVG-SIPKWQ-TFLFGYFMGQVAGFGVTAGAHRYWTHRSYKATFPL--QIILIICYSVAGQNNIYNWVRDHRIHHKFSET---SAD-PHDARRGFFFSHVG---WLMMKK-----HPDVIREGRKISMR--DIANNPLVQFHTKYFDVFKFVFCFFLPTLIPVYGWHETWTNAILSQPILRYALSLNFTWSVNSFAHIWGNKPYDRHI**[RPAE]**NWGVSAVAMGEGWHNYHHTFPW--DYKASELA--YINNITTSLLNLFAKIGWAYDLKEASPSLIK
Papilio xuthus	XP_013164746.1	PREDICTED: acyl-CoA Delta(11) desaturase-like	PxutΔ11_8	IR-WTSAIPIVIFHLITALSIIYSVIVGS-IPKWQ-TFLFGYFMGQVAGFGVTAGAHRYWTHRSYKATFPL--QIILIICYSVAGQNNIYNWVRDHRIHHKFSET---SAD-PHDARRGFFFSHVG---WLMMKK-----HPDVIREGRKISMR--DIANNPLVQFHTKYFDVFKFVFCFFLPTLIPVYGWHETWTNAILSQPILRYALSLNFTWSVNSFAHIWGNKPYDRHI**[RPAE]**NWGVSAVAMGEGWHNYHHTFPW--DYKASELA--YINNITTSLLNLFAKIGWAYDLKEASPSLIK
Papilio xuthus	XP_013164760.1	PREDICTED: acyl-CoA Delta(11) desaturase-like	PxutΔ11_9	IV-YRNILTFGYAHLTALLY----------PLRIS----------------ITAGAHRLWTHRAYKAKLPL--QIILVVLNSLAFQNTAIDWVRDHRLHHRYSDT---DAD-PHNATRGFFYSHIG---WLLVRK-----HEEVKRRGKFIDMS--DIYANPVLCFQRKYAIPFIGTICFVLPTIIPMYFFGESLKTAWCIA-VLRYVLNLHIAFLVNSAAHIWGNKPYDRTI**[KPVQ]**SLPVSFVAFGEGFHNYHHVFPW--DYRTAELG-NNYLNLTTIFIDFFAKIGWAYDLKSVNDDMIK
Papilio xuthus	XP_013177212.1	PREDICTED: sphingolipid delta(4)-desaturase DES1	PxutΔ4	LYGYDPLFKWVVLAM-VLMQLLMLPLVK--YMSWPVLLVVAYCFGGVINHSLMLAIHEIAHNLAFGHNRPLHNRLFGFFANLPIGVPVSISFKKYHLEHHRYQGDEVIDVDLPTLLEAKLFCTTGGKLVWLFLQPFFYALRPLVVRPKPPTPLE-----------------MINLVIQLFFDAVVVELFGW-RALWYLLLGSFMAMGIHPVAGHFVSEHYMFRKGFETYSYY------GPLNWITFNVGYHNEHHDFPAVPGRRLPEVK-RIAPEFYDDLPHHTSWSSVLYDFVMDPDIGPY
Spodoptera exigua	AFO38464.1	delta-9 desaturase 16-18	SexiΔ9_1	LV-WRNIILFAYLHL-AALYGGYLFLF---SAKWQ-TDIFAYILYVISGLGITAGAHRLWAHKSYKAKWPL--KVILIIFNTVAFQDAAMDWARDHRMHHKYSET---DAD-PHNATRGFFFSHIG---WLLVRK-----HPDLKEKGKGLDMS--DLLADPLLRFQKKYYLVLMPLACFVMPTMIPVYLWGETWTNAFFVAAMFRYAFILNVTWLVNSAAHKWGDKPYDKSI**[KPSE]**NMSVAMFALGEGFHNYHHTFPW--DYKTAELG-NNKLNFATAFINFFAKIGWAYDMKTVSDDIVK
Spodoptera exigua	AFO38465.1	delta-9 desaturase 14-26	SexiΔ9_2	VV-WRNVFAFVYLHA-AALYGFYLMFTG--KVRIW-TILFGLLFAIMAGMGVTAGAHRLWAHRSYKARWPL--RLFLALMQTMAFQNHIYEWVRDHRVHHKFTET---DAD-PHNARRGFFFSHIG---WLMVRK-----HKDVFEKGATVDMS--DLEKDPIVMFQKKTYMVLMPLLCFVIPAWIPCYFWGENPWYSWYVASITRYTVALHFTWLVNSAAHIWGNRPYDKNI**[GATD]**NKAVAICAFGEGWHNYHHVFPW--DYKAAELG-NYSTNLSTALIDFAAKHGLAYDLKTVSAEMIR
Spodoptera littoralis	AAQ74259.1	delta-11 desaturase	SlittΔ11	IV-YFNIITFAYWHI-AGLYGLYLCFT---STKWA-TVLFSFFLFVVAEVGVTAGSHRLWSHKTYKAKLPL--QILLMVMNSLAFQNTVIDWVRDHRLHHKYSDT---DAD-PHNASRGFFYSHVG---WLLVRK-----HPDVKKRGKEIDIS--

				DIYNNPVLRFQKKYAIPFIGAVCFVLPTLIPVYGWGETWTNAWHVA-<br>MLRYIMNLNVTFLVNSAAHIYGKRPYDKKI**[LPSQ]**NIAVSIATFGEGFHNYHHVFPW--DYRAAELG-<br>NNSLNFPTKFIDFFAWIGWAYDLKTVSKEMIK
Spodoptera littoralis	AAQ74257.1	delta-9 desaturase	SlittΔ9_1	IV-WRNVILMGLLHI-GGVYGAYLFLT---TAMWR-TSLFAVFLYICSGLGITAGAHRLWAHKSYKARLPL--<br>RILLTLFNTLAFQDAVIDWARDHRMHHKYSET---DAD-PHNATRGFFFSHVG---WLLVRK-----HPQIKAKGHTIDLS--<br>DLKNDPILRFQKKHYLILMPLVCFILPCYIPT-<br>LWGESLWNAYFVCSIFRYVYVLNVTWLVNSAAHLWGAKPYDKNI**[NPVE]**TKPVSLVVLGEGFHNYHHTFPW--DYKTTELG-<br>DYPLNLTKLFIDFMAAIGWAYDLKTVSSDVIQ
Spodoptera littoralis	AAQ74258.1	delta-9 desaturase	SlittΔ9_2	LV-WRNIILFAYLHL-AALYGGYLFLF---SAKWQ-TDIFAYILYVISGLGITAGAHRLWAHKSYKAKWPL--<br>KVILIIFNTVAFQDAAMDWARDHRMHHKYSET---DAD-PHNATRGFFFSHIG---WLLVRK-----HPDLKEKGKGLDMS--<br>DLLADPVLRFQKKYYLLLMPLACFVMPTMIPVYLWGETWTNAFFVAAMFRYAFNLNVTWLVNSAAHKWGDKPYDKSI**[KPSE]**NMSVA<br>MFALGEGFHNYHHTFPW--DYKTAELG-NNKLNFTTAFINFFAKIGWAYDMKTVSEDIVK
Spodoptera litura	AGH12217.1	delta 11 desaturase	SlituΔ11	IV-YFNLVSFAYWHI-AGLYGLYLCFT---SAKWA-TILFSFFLFVVAEVGVTAGAHRLWSHKTYKAKLPL--<br>QILLMVMNSLAFQNTAIDWVRDHRLHHKYSDT---DAD-PHNASRGFFYSHIG---WLFVRK-----HPDVKKRGKEIDIS--<br>DIYNNPVLRFQKKYAIPFIGAVCFALPTLIPVYGWGETWTNAWHVA-<br>MLRYIMNLNVTFLVNSAAHIYGKRPYDKKI**[LPSQ]**NIAVSIATFGEGFHNYHHVFPW--DYRAAELG-<br>NNCLNFTTKFIDFFAWIGWAYDLKTVSKEMIK
Spodoptera litura	AGH12218.1	delta 9 desaturase	SlituΔ9	IV-WRNVILMGLLHI-GGVYGAYLFLT---TAMWR-TSLFAVFLYICSGLGITAGAHRLWAHKSYKARLPL--<br>RLLLTLFNTLAFQDAVIDWARDHRMHHKYSET---DAD-PHNATRGFFFSHVV---WLLVRK-----HPQIKAKGHTIDLS--<br>DLKNDPILRFQKKHYLILMPLVCFILPCYIPT-<br>LWGESLWNAYFVCSIFRYVYVLNVTWLVNSAAHLWGAKPYDKNI**[NPVE]**TKPVSLVVLGEGFHNYHHTFPW--DYKTAELG-<br>DYSLNLTKLFIDFMAAIGWAYDLKTVSSDVIQ
Plutella xylostella			PxylDES1	IV-WRNVILMAVLHI-GGLYGAYLFLT---KAMWT-TCFFAVFLYICSGLGITAGAHRLWAHKSYKARLPL--<br>RILLTLFNTIAFQDSVIDWARDHRMHHKYSET---DAD-PHNATRGFFFSHVG---WLLVRK-----HPEIKNKGHTIDMS--<br>DLWADPVLRFQKKNYLLLMPLCCFVLPTMIPT-<br>LWGESLWNAYFVCALFRYTYVLNVTWLVNSAAHKWGDKPYDKDI**[NPVE]**TRPVSLVVLGEGFHNYHHTFPW--DYKTAELG-<br>HYSLNLSKLFIDTMSLLGMAYDLKTVSRDVIE
Plutella xylostella			PxylDES2	IV-WFNVATFTYAHL-AALYGLYLGLT---AAKWE-TIIFSVILYSMAMIGVTAGAHRLWSHKAFKAKLPL--<br>QIILMLFNSVAFQYTAYHWVREHRLHHKYSDT---DAD-PINAHRGFFYAHVG---WLLVRK-----HPDVKKAGPTIDMS--<br>DLDANPVVKFQKDYAIPFCGTICFLLPTLIPVYCWGESLNVAWHVA-<br>VFRYIANLNVMFLVNSAAHLWGYRPYDHTM**[LPAE]**NLSVALASFGEGFHNYHHTFPW--DYKAAEFG-<br>NNRLNFTTAFIDFFAKIGWAYDLKSVSDEMIE
Plutella xylostella			PxylDES3	IV-YFNLLTFGYWHL-AGAYGLYLCFT---SAKFA-TVLFAILTYTAAEIGITAGAHRLWSHKAYKAKLPL--

		QIILMTFNTLAFQNSAIEWVRDHRLHHKYSDT---DAD-PHNATRGFFYSHVG---WLLVRK-----HSEVKKRGKTIDMS--DMYSNPVLAFQKRYIVPWVILVTFLLPTVIPVYFWNESLWTSWHVT-MLRYVANLNATFLVNSAAHLWGYKPYDKNI**[MPAQ]**NISVSLATFGEGFHN--
Plutella xylostella	PxylDES5	YV-WRNILLFAYLHL-TALYGGFLFLT---SAKWQ-TDVFAYILYVVSGLGITAGAHRLWAHKSYKAKWPL--RLILVIFNTIAFQDSAIDWARDHR--
Plutella xylostella	PxylDES6	IR-WPDLIVQVLLHL-VSLCGLYLIITN--SVRFY-TTLFALATIYTSGFGITAGVHRLWSHRAYKANTPL--RVILALLFTITGQRDIYTWALDHRVHHKYSET---CAD-PHDVRRGFWFAHVG---WLVLTP-----HPAVEDRRRALREVSKDLDSDPVVYWQKILFIPLFGILNVALPIWIPVHCWGESVINAFIVSFVLRFTATLNIAYSVNSFAHLWGNKPYDKFI**[SPVE]**NQVVSLAALGEGWHNYHHVFPW--DYRTSELG---RVNISTNFINAFAKIGWAYDLKAASSAMIT
Plutella xylostella	PxylDES7	VV-YFNLLTFGFAHL-SWAYGVYLIFT---AAKWQ-TTVFALAYGIAGGLGITAGAHRLWAHRTYKAKMPL--QIILMILNTIAFQNTAMDWVRDHRLHHKYSDT---DAD-PHNATRGFFYSHVG---WLLVKK-----HPEVRRRGKLIDMS--DIRNNPVLDFQKRYAIPLVLTLTFILPAFIPWYFWGETLNSSWHVATMFRYVTGLNLTFLVNSAAHFFGYKPYDKSI**[LPAQ]**NKFVAFAALGEGWHNYHHIFPW--DYRTAELG-NNSLNLTTHFIDFFAKIGWAYDLKAAPEDMIK
Plutella xylostella	PxylDES8	VN-WLNVTFIIGIPLAGCVAAFWT------PLKWQ-TALWAVVYYFWTGLGITAGYHRLWAHKSYNASLPL--SVFLALVGGGAVEGSIRWWSRDHRAHHRYTDT---NKD-PYSVRKGLLYSHLG---WMVMKQ-----NP--KRIGRT-DIT--DLNEDPVVVWQHKHYIKVVIFMGLIFPSAVAGLLWND-WKGGFIYAGILRIFFVQQATFCVNSLAHWLGDQPFDDRN**[SPRD]**HVITALVTLGEGYHNFHHEFPS--DYRNAIE--WHQYDPTKWSIWLWSKLGLASNLKQFRANEIE
Plutella xylostella	PxylDES9	--AFQNTAYTWIRDHRLHHKYSDT---DAD-PHNATRGFFFSHVG---WLLVRK-----HEDVKKYGKLIDMS--DIENNPVLRFQKKYALPWCGMVCFVLPTLIPMYCWGESLNCAWHIAVVLRYVANLNVTFLVNSAAHIWGYKPYDKNM**[LPGE]**NISVAIASFGEGFHNYH--
Plutella xylostella	PxylDES10	AD-WPAVLFFIHIHL-LSLYGVWLLLF---EVKLM-TVLLLVALTSFGLLGVTTGAHRLWAHRTYKASTGL--RVALMICQTLAGQGSIYEWVRYHRLHHAHFGT---DSD-PYNYKQGFLHAHMM---TRLRQL-----SPHQQRLMEEVDVA--DLEADWVVMFQKKFYWLLYGIVFLLLPLNAPLEYWDDSVLSSVFVIGFLRYLVVLHAAWLVESGICVWGLKPGEKYP**[ADSN]**MVFILAKTF---WPHYHYLVPQ--DYKSGEYG-TYDCGCSTAFIRVWAALGLATDLQTVDTATAQ
Plutella xylostella	PxylDES11	--AFQNTAYTWIRDHRLHHKYSDT---DAD-PHNATRGFFFSHVG---WLLVRK-----HEDVKKYGKLIDMS--DIENNPVLRFQKKYALPWCGMVCFVLPTLIPMYCWGESLNCAWHIAVVLRYVANLNVTFLVNSAAHIWGYKPYDKNM**[LPGE]**NISVAIASFGEGFHNYH--

Note: The signature motif of each sequence was marked with boldtype and bracketed by square brackets

Supplementary Table S3. Sequence details used in the alignment and phylogenetic analysis of OBP and PBP genes.

Species name	Accession number	Sequence definition	Abbreviation	Amino acid sequence
Bombyx mori	BAH36761.1	odorant binding protein	BmorOBP1	---------------------------------MVGFKILFTLCAFAICFC-------------ASAYVDTLQKCGKSDAVCQKKLLQS----VLKSIS------------------------K------TGIPEL---DIPQI-------DPIQLKGFNVAILDLVNITLVDGVAKGVKDCTVNKFEANFDDLHASIEL-VCDITI---------KGHYSVYSGSPLIKNFLGGDNIHGDGNGKAKIEKFKVAFDFDFTVEKRGDDLFIKSSIEKM-------------KYTYDVLGKMVFAADNLYVGN-----KEQSASIVKL----MNENWRI----L---MDMVGK----QFVEKAM-NFVFNFTQKFFSNVPTKNYILDDLENYVSS
Bombyx mori	BAH79159.1	odorant binding protein	BmorOBP2	--------------------------------------MYRILLLFCVIY--------------PSNGAIEISKHLKVCNRNSLDLNDC----IVEAVR------------------------DGIEKMATGIEELDIPPLDPF-------FQDELKVEYKNNQIAVKMLIKNIYVEGLKGSTVHDARVRADEDNFYMEVDLSAPSI-------VIRGDF--------------------KGEGQ---------YNALKVKAYGDF---NTSMSDLIFTWKLDGVPEKNGTDTYVRIKSFYMRPDVGNMISHLNNENPETRELTNLGTSFLNQNWRV----LYRELLPYAQ----SNWDKIGTNVANKIFSKVPYDQIFPSGT----------
Bombyx mori	BAH79158.1	odorant binding protein	BmorOBP3	---------------------------------MHVTYFVTFLSVICIVNSTP-----------PSFVNRCKYGDSECTKESTVVAIPI----FAAGLP------------------------E------YGVEKL---DPVTFNKVDASSPNLKFILTD-------------VEVTGLSGCK---PKQIQHGSKLELKI-LCQAKLNGNYELNGQVLVL--------------------PIKGKGKIHVDLKTTQINVDANYEEK---LGDDGKKHWHITKWSYTFELQDK-SDVVFENLFDGNEVLGQAARELIANNGNDIIKEIGSPM---IKAAVARV---MKNIER----FFKAIPVEDLILN-------------------------
Bombyx mori	BAH36763.1	odorant binding protein	BmorOBP4	---------------------------------MANPVLLLTFVLMTXSMARLKSTE-------APKSKTALFNDQDNMGYEELDMEEI----MSACNE------------------------S------FRIEYAYLESLNDS---------GSFP----------------DETDKTPKCY----------------I-RCVLEK---------TEIL--------------------SENGV---------LNPATAALVFAG----ERNGKPM-------------SD-LEEMAVACADRH-----------EKCKCEKAYN----F---VKC----LM--YMEIDK----YEKKN---------------------------------
Bombyx mori	BAH36762.1	odorant binding protein	BmorOBP5	-----------------------------------MTSFIVFFVLSVLTL--------------KYSDALT----DEQKDKIQSKFIEI----GAECIV------------------------E------HPISID---DINSF-------KNKKF------------------PSGVNAGCF----------------V-ACIFNK---------IGLF--------------------DDKGN---------LSHSSALEKAKG---IFNADEEV-------------KN-LEEFLNRCAKVNEEAVGD-----DVKGCERAKL----A---YNC----L---IENSLE----FGFNIDF-------------------------------
Bombyx mori	BAH36760.1	odorant binding protein	BmorOBP6	----------------------------------MTSKVLLSCVVLAVLA--------------TTVLAEDSRKLVSFAPEVAKKLKVL----IQECLN------------------------E------NGLGED---AIEVI-------RAGEYR------------------EDEPFQNL----------------V-YCAYKK---------FGAL--------------------DENNR---------IISQVAAASFPK------------------------DIDVVTVIESCGKED-----------GNTPVDQVFK----Y---FKC--------FQKNSP----VRMQLY-------------------------------
Bombyx mori	BAH36759.1	odorant binding protein	BmorOBP7	-----------------------------------MSANSFVVLAFCALA--------------VGVNALT----EEQKAEITKSSLPL----IAECSK------------------------E------FSVNQGDIDAAKKL-----------------------------GDPSGLNSCF----------------V-GCFMKK---------AGII--------------------NASGL---------FDVAATIEKSKK---YLTSEEDL-------------KA-FEKLTEMCAPENDKPVSD-----SDKGCERAKL----L---LDC----F---VANKGS----FSVFSL-------------------------------
Bombyx mori	BAI44701.1	odorant binding protein	BmorOBP8	----------------------------------MLRVVVICVCFLVIAP--------------YGINASS-------LDDLKTVYENV----IKECVG------------------------D------YPITAA---DLELI----------KAR----------------QIPNDDIKCV----------------F-ACAYKK---------TGMM--------------------TEEGM---------LSVEGIKDMSQK--YLSDNPEQL-------------RK-SKEFAEACSSVNDQQVSD-----GTKGCERAAL----I---FKC----S---TEKITN----FGFEL---------------------------------
Bombyx mori	BAI44700.1	odorant binding protein	BmorOBP9	-----------------------------------MYTNFILIFYFGISI--------------YDVRASS-------LDDLKTVYENV----IKECVG------------------------D------YPITAA---DLELI----------KAR----------------QIPNDDIKCV----------------F-ACAYKK---------TGMM--------------------TEEGM---------LSVEGIKDMSQK--YLSDNPEQL-------------RK-SKEFAEACSSVNDQQVSD-----GTKGCERAAL----I---FKC----S---TEKITN----FGFEL---------------------------------

Bombyx mori	BAI22690.1	odorant binding protein	BmorOBP10	-----------------------------------MKSVVLICLAFAVFN--------------CGADNVH------LTETQKEKAKQY----TSECVR------------------------E------SGVSTE---AINAA-------KIGKY------------------SKDKAFKNF----------------V-LCFFNK---------SAIF--------------------NSDGT---------LNMDVALAKLPP-----GVNKSE----------------AQSVLKQCKNKT-----------GQGAADKAFE----I---FRC----Y---YKGTKS----HILF----------------------------------
Bombyx mori	BAI22689.1	odorant binding protein	BmorOBP11	-----------------------------------MMGYACVFVILAVLQ--------------AISAEDPPGLPPFLKDAPEKCRSPPRVKNPNECCI----------------------SE------PFFKEA---DFIEC-------GIEKP-----------------GSERGPPDCS----------------KQNCLLKK---------YNLL--------------------KNDET---------PDIEAIKSLLDK---YIEKNPSF-------------KS-SVEKAKECLREDLPG--------PPQICLANRM--------TLCIGTVL---LMECPD----EKWNTTDDCKAFKDHMTECQKYFPK-------------
Spodoptera litura	ALJ30188.1	putative odorant binding protein OBP1	SlitOBP1	-----------------------------------MLNSIFLLFTFGVFS--------------LYAEALS-------MDDLKQKYVDN----ILECSK------------------------Q------YPIDRA---DAEQL---------QNRI----------------MPDKEPIKCL----------------F-ACVYKL---------AGMM--------------------NDQGE---------LSVEGVNAISRK--YLAEDPEKL-------------QK-SEEFTEACRSVNDAPVTD-----GTRGCDRAAL----I---FKC----T---IEKSPD----FNFV----------------------------------
Spodoptera litura	ALJ30189.1	putative odorant binding protein OBP2	SlitOBP2	---------------------------------MTCSQALALLALVAI----------------SQQATTGCKNCIMLGKEEKAMFRAH----SDACVA------------------------A------SRVEPR---LVDAM-------LAGE------------------LLDEPALRKH----------------V-YCVLLK---------CKLI--------------------SKDGK---------LQKAAVLGKMAA-----RPDAKN----------------ATKVLESCADQT-----------GDTPEDLAWN----L---FRC---GY---DKKALL----FDYMPTNVASETDNNS----------------------
Spodoptera litura	ALJ30191.1	putative odorant binding protein OBP4	SlitOBP3	---------------------------------------MYRFVILSIVL--------------VSALADD-----IDIRECGRIFHPP----PHGCCK------------------------ANNAVKNKDMLAE---ELKDC-------FDG--------------------SGPKDPMKCE----------------IDLCIAKK---------KGFA--------------------TDDGK---------LDIKKFEEVITK---EVGSDKDL----------------LDEIKTNCINGDLNNYG------PPEFCDFMKI--------KHC----VTL-HMMNHC----SEWSDDGNCKVVKELVGKCAKVI---------------
Spodoptera litura	ALJ30193.1	putative odorant binding protein OBP6	SlitOBP4	-----------------------------MFRCNMVKVTYIALFVVAVSL--------------SSVQADD------KNSKPEFNLDTI----TFQCAQ------------------------K------FDISEEQFSKAIMT-----------------------------FDASLLAPCF----------------W-SCCFMK---------VGVL--------------------NSEGQ---------YDSDSTLNLAKN--MFKNEEYKK----------------VEEILKKCVSVNDESVSD-----GSAGCERSFL----L---ASC----M---FENAKK----TFTIPSRPTVI---------------------------
Spodoptera litura	ALJ30194.1	putative odorant binding protein OBP7	SlitOBP5	----------------------------------MITSCLLVLSAVVQVLLAKQPVFESGPPEPWGPPERTSHPGQFQPRVPKRCWVPPQRINVYNCCP-----------------------IP------TLYPDE---DMQSC-GFEKLSENKPQK----------------PVYRPEGTCK----------------EGYCVMGK---------FDLL--------------------LANNS---------VDYVKFREYLDN--WAESYPEFA-------------NA-IHIAKEECAQDGGPEVPPICEPDKLFLCLTSTI----F---WNC----KLRDGEGCAALQEHMNECKQYYTRVMAPTIKDFEVR----------------
Spodoptera litura	ALJ30195.1	putative odorant binding protein OBP8	SlitOBP6	-------------------------------MVRKISGLLCCLCVFGISFS-------------DSAISADSESRCRNPPTAPQKIERV----ITLCQD------------------------E----IKLSILREALDVIKEEHTMPAQRRRDKREVPFT------------HDEKRIAGCL----------------L-QCVYRK---------VKAV--------------------DGYGF---------PTLEGLVGLYSD----GVNERGY-------------FMAVLEASRECLMKNHDKFSRTVPMDNGRNCDISFD----I---FEC----I---SDRIGE----YCGTSGL-------------------------------
Chilo suppressalis	AGK24577.1	odorant-binding protein 1	CsupOBP1	----------------------------------MRCCAVLFVLAFIGCI--------------YAEQEIV-----HLPPEKVAQILPV----AMQCVG------------------------E------SSVPPE---VIFQY-------ASGKS-----------------LGNDKKYQKF----------------I-HCVFTK---------TGYA--------------------DETGH---------INIDKAMEVFPK-----GTDKEA----------------VKKIMEECSKER-----------GEDPPETSFK----F---AKC----F---RKKAP-----VRIAL---------------------------------
Chilo suppressalis	AGK24578.1	odorant-binding protein 2	CsupOBP2	-----------------------MGTVSTEHYPAHHVINMKAFIVLAVCI--------------VAAQALT--------DEQKEKLKKH----KSECLA------------------------E------TKVDEQ---LVNKL-------KAGDYK----------------SDNEALKKYA------------------LCMLIK---------SELM--------------------TKEGK---------

				FKKDVALAKVAN-----PADKPQ----------------VEKLIDTCSANK-----------GNTPHQTAWN----Y---VKC----Y---HEKDPK----HAIFL--------------------------------
Chilo suppressalis	AGK24579.1	odorant-binding protein 3	CsupOBP3	---------------MKRSRVKRPRDKNQKMDRVLTDFIMIIFVLFAIYLVTAF----------EPMTKKEHIAKFNKMNDEVEPFRKN----LTECSR-----------------------Q------VKASMV---DIENF-------LKRI------------------PQTSMQGKCF----------------V-ACILKR---------NAII---------------------RNNK---------VDDNGLLEANRA--VYGDDSEVM-------------SR-LKAAVGECKQVVDN---------IFEICEYASI----F---NDC----M---HMKMEHILDKVTLERRMEALGQMSSDTDTWTDEEDEMLKLLKDEL---
Chilo suppressalis	AGK24580.1	odorant-binding protein 4	CsupOBP4	----------------------------------MFRLFLCLVVFVTTS--------------YGDLLTQ--------ERSRGATLKP----ISACCG-----------------------I------PELGDS--KPLTEC-----------------------------SKPKLLGPCN----------------DIQCVFEK---------SGFL--------------------VDRNT---------LNKDVYKKHLRK--WAEAHDSWT------------EA-VERAIADCVDKELRQY-------LDYPC-RAYD----V---FTCTGIAM---LKKCPQ----EAWKC--------------------------------
Chilo suppressalis	AGK24581.1	odorant-binding protein 5	CsupOBP5	---------------------------------MKLTLFPAFLLFTSIL--------------------------GLKDEHEENLKRW----HMECFQ-----------------------E------TKVNPD---LVLKL-------KMGNW-----------------QIKNKLLKEW----------------I-LCVFNK---------YDLM--------------------SKEGV---------FKLDTAMSLVPS------ADRDM----------------IEDYIDACLPKH-----------IAEPLDIAWK----Y---AKCYHVGAKDPINKNKR----LHYMTLFY-----------------------------
Helicoverpa armigera	AEB54580.1	OBP1	HarmOBP1	---------------------------------MSKFTFFVLCVVAVSL--------------SKVYASD-----EDKAKLHEALKPL----VEECMK-----------------------D------HEVSLD---DLKAA-------KEA-------------------KSADGVKPCF----------------L-ACVYKK---------AEVL--------------------NDKGE---------FDADHALEKLKE---FVSDEDVL-------------AK-VAEVGNTCKAVNDKAVSD-----GDAGCERAAL----L---TAC----F---LEHKAE----ILV----------------------------------
Helicoverpa armigera	AEB54586.1	OBP2	HarmOBP2	--------------------------------MMDRKRLCLLIIALFLA--------------QGSDAMS-------RQQLKNSGKML----KKNCMN-----------------------K------NQVTED---QIGSI-------DKGKF------------------VEDKKVMCY----------------I-ACIFEM---------TNVV---------------------KNNK---------LNYDASIKQIDL--MYPPDLKES----------------AKAAVEKCKDVQKK---------YKDICEASYW----T---AKC----M---YDFKPE----DFIFA--------------------------------
Helicoverpa armigera	AEB54582.1	OBP3	HarmOBP3	---------------------------------MSKFTCFVLCVLAVSL--------------GEVRSNA-----LEKAAIRAAVYPL----IVDCAK-----------------------E------HGVTLEQLKAAKAS-----------------------------HSAEGINPCF----------------Q-SCVYKK---------TGIF--------------------NDNGE---------YDVANAKTKLQK---FVTDEDEY-------------AR-IAEVGKTCASVNDKSVSD-----GAAGCERAAL----L---TAC----FLEHRAQIII---
Helicoverpa armigera	AEB54584.1	OBP4	HarmOBP4	---------------------------------MSKLTCVVFAAVAVVF--------------SNVNADD-----ETRASFRQVLGPL----VMECRN-----------------------E------FGITED---DLKKA-------QQE-------------------RSPDALKPCF----------------I-ACVFKK---------FGII--------------------TSAGK---------YDSDASISRIKD---VVKNDDLL-------------AK-LKSVGEKCNSVNDASVSD-----GDAGCERAAL----L---AKC----F---IENKSE----LSI----------------------------------
Helicoverpa armigera	AEB54581.1	OBP5	HarmOBP5	---------------------------------MSKFTCLVLCVVAASL--------------SQAYASE-----EEKAAFREAIKPI----VEECSK-----------------------E------HGVSHDELKSAKDN-----------------------------QNADSIKPCF----------------L-GCVYKK---------AEVF--------------------NSKGE---------YDVDKALEKLKK---FVSNDEAY-------------AK-FAEVGKKCASVNDKAVSD-----GDAGCERGAL----L---TAC----FLEHKAEVPL---
Helicoverpa armigera	AEB54587.1	OBP6	HarmOBP6	---------------------------------MSKFTCLLLCVVAVSL--------------SKVHATE-----EEKEAIRAAVRPI----MQECGK-----------------------E------HGVTLD---DLKAA-------KAA-------------------HSADGIKPCF----------------Q-SCVYKK---------AGIF--------------------NDNGE---------YDIANAKTKLQK---FVTNDEEY-------------AR-IAEVGKMCASVNDKPVTD-----GAAGCDRAAL----L---TAC----F---LEHRAQ----III----------------------------------
Helicoverpa armigera	AEB54591.1	OBP7	HarmOBP7	---------------------------------MFRFGVLSFVVLLFCM--------------ESSYALS----SEEELSIKEALHPF----VVECAE-----------------------E------YGMTEEMFEEAKKK-----------------------------GSAEDIDPCF----------------M-SCFLKK---------TGFF--------------------DDSGK---------FDAEKSISFAKE---HITSESAI-------------KF-LEAGAGECVKINDEDVSD-----GENGCDRAKL----L---FDC----LTELKKKMSE---

Helicoverpa armigera	AEB54589.1	OBP8	HarmOBP8	---------------------------------MLLIEIVKFLTLVAMCE-------------------------AMTMKQIRNTGKMM----RKSCQP------------------------K------NNVADE---QIDPI-------AEGVF------------------NEDKEVKCY----------------M-ACIMKM---------ANTI---------------------KNGK---------LNYEAAIKQADL--LLPDDIKEP----------------AKEAITACRKVADA---------YKDICDASFH----I---TKC----I---YTQNPG----IFYFP----------------------------------
Helicoverpa armigera	AEB54592.1	OBP9	HarmOBP9	--------------------------------MCKFSVLFLYSAVMAVNI-------------WSASCIS----EEDKAAIITAIAPL----AQNCGS------------------------E------CGLDND---DFEKY----------------------------KEDGSDMDPCF----------------K-ACLMTQ---------MGVL--------------------DKEGK---------YDGKGLHKAMEE-ADYPGDKDDA-------------QK-FLDELDRCFDAKGDNSGSD----EEAKMKRADV----L---FRC----M---QDMKEK---
Plutella xylostella			PxylOBP1	-----------------------------RESSAMSTRRLLLFSMLIAAV--------------FGGKSKP-----EFSEEIKEIIQHV----HNECVG------------------------K------TGVAED---DITNC-------ENGVF------------------KDDQKLKCY----------------M-FCLLEE---------ASVA--------------------DENGV---------VDYEMMISLIPE------DYTER----------------VSKMIMACKHLDTP---------DKDKCQRAFD----V---HKC----S---YEKDPD----LYFLF----------------------------------
Plutella xylostella			PxylOBP2	----------------------------MVGLDGLPPNTMERLALLLAVL--------------AAVRAEF------PTKEFVEMLKPV----ILKCEE------------------------K------TGVNKD---FVDQF-------NKGT------------------MVDDPTFKCY----------------L-KCMFLE---------FEVL-------------------DPTSGH---------FRYEKMLGILPQ------EMKPI----------------AMEMGKNCIHFKGEE--------GSDLCEVSYQ----L---HQC----W---QKASPQ----HYFLLRR-------------------------------
Plutella xylostella			PxylOBP3	--------------------------------KETNMKTIVFVCLVATAA--------------AAANVKH----AHLTKDQSARVHEY----GMECMK------------------------K------TGVNPE---LVAKA-------KKGEF-----------------TDDEALKKFT------------------LCFFQK---------TGIL--------------------TSDGK---------LNEEVALSKLPA-----EVDKAA----------------VKKVLDECKKKT-----------GKDMADSAFE----V---FKC----Y---HKATPT----HVSF-----------------------------------
Plutella xylostella			PxylOBP4	-------------EGQYPKNPVGEHWLCTKLKIKMKMGSSMYVAFLAVAV--------------AALFGNTHAISDENREKLKKEMAPI----FMECAK------------------------E------GSLNLD---DLKQY-------KGVKE-----------------LPADEGVTCF----------------F-ACAFKK---------IGMI--------------------DDKGM---------FAVEESVERGKK---YMDSEEKQ-------------KH-LEEAANTCASVNDESVSD-----GDKGCERAKH----L---YEC----L---IKQAEK----FGLELPTSAV----------------------------
Plutella xylostella			PxylOBP5	-------------------------------LPAMEGHKVLTLLLFGVLV--------------VSVSCMD--------EEMAELAKMI----RDNCGA------------------------E------TEVDMG---LIDQV-------NGGAD-----------------LMPDGKLKCY----------------I-KCVMET---------AGMM---------------------SEGE---------VDVEAVLALLPE------DFKAK----------------NEKSLRACGTQK-----------GADDCDTAFQ----T---QAC--------WQKANK----ADYFLV--------------------------------
Plutella xylostella			PxylOBP6	-----------------------------------MTCFFPVFIFITLVIN--------------SVICSVC----SRFLLDMSNSCVLF----LLLCVS---------------LTHGNLFLEELRKKGASLKPLSACCDIPDL-------GDPEHLAAC-------------SSPKLQGPCN----------------DIQCVFEK---------SGFL--------------------VDKQT---------LDKEAYRSHLRR--WAEEHQGWS-------------EA-AEKAVRDCVERELRQY-------LPRPC-RAYD----A---FTCTGIAM---LKKCPE----EAWKCSHKK-----------------------------
Plutella xylostella			PxylOBP7	MSSFFKNSNFIRAFAFSGSCVFVVGNCWIEKMVRRLSALACCFCMFAISLC-------------GSAIPSESDTRCKNPPTAPQKIERV----ITLCQD------------------------E----IKLSILREALDVIKEEHTMPAQRRRNKREVPFS------------HDEKRIAGCL----------------L-QCVYRK---------VKAV--------------------DGYGF---------PTLEGLVGLYSD----GVNERGY-------------FMAVLEASRGCLMRHHDRFSRTVPMDNGRNCDVSFD----I---FEC----I---SDRIGE----YCGNSGL-------------------------------
Plutella xylostella			PxylOBP8	------------------------VLASKGSTTIINKIKMKFLVVFAICL--------------VAAQALT--------DEQKEKLKKH----KTECLA------------------------E------TKPEVE---HVDKL-------KNGDYT----------------TENEALKKYA------------------HCMMIK---------SELM--------------------TKDGK---------FRKDVALAKVPN-----PADKPM----------------VEKLIDTCLANK-----------GDTPQQTAWN----Y---VKC----Y---HEKDPK----HAIFL---------------------------------

Plutella xylostella			PxylOBP9	--MPPSLMSRQVPELLMARSLPVLHPE-----HSCLG------------------------S---------------TLGTM-----------<br>------------------EKPFARSPLW----------------YRSCNARS-----PPDMQTYM--------------------STHGA---------YSAGSAGACYPH----------A----------------<br>LLDVQSSTGDVSPRT--------NYKGNSRAP--
Bombyx mori	CAA64443.1	Pheromone binding protein	BmorPBP	---------------------------------MSIQGQIALALMVNMAV--------------GSVDASQ-----EVMKNLSLNFGKA----LDECKK------------------------E------<br>MTLTDAINEDFYNF-------WKEGY-----------------EIKNRETGCA----------------I-MCLSTK---------LNML--------------------DPEGN---------<br>LHHGNAMEFAKK---HGADETMA-------------QQ-LIDIVHGCEKSTPA---------NDDKCIWTLG----V---ATC----F---KAEIHK----LNWAPSM-<br>DVAVGEILAEV-------------------
Helicoverpa armigera	CAC08212.1	pheromone binding protein (PBP-Harm)	HarmPBP1	---MNFAKP----LEDCKK------------------------E------MDLPDSVTTDFYNF-------WKEGY-<br>----------------EFTNRQTGCA----------------I-LCLSSK---------LELL--------------------DQELK---------LHHGKAQEFAKK---HGADDAMA-------------KQ-<br>LVDLIHGCAQSTPDV--------ADDPCMKTLN----V---AKC----F---KAKIHE----LNWAPSM-ELVVGEVLAEV-------------------
Helicoverpa armigera	ACD01993.1	pheromone binding protein 2	HarmPBP2	--MM--------------GSAMSSK-----ELLTKMTGGFTKV----VDACKT------------------------E------LSVGDHIMQDMYNF-<br>------WREEY-----------------QLVNRDLGCM----------------I-MCMTAK---------LDLI--------------------GDDQR---------MHHGKAEEFAKS---HGADDALA----<br>---------KQ-LVGLIHGCETQHQA---------IEDHCSRALE----I---AKC----F---RTKIHE----LKWAPSM-EVIMEEIMTAA-------------------
Helicoverpa armigera	AAO16091.1	pheromone binding protein 3	HarmPBP3	---------------------------------MGSRHVFFALVVLAVSV--------------RKAEPSK-----DAMQYITSGFVKV----LEECKH------------------------E------<br>LDLNEQILADLFHF-------WKLEY-----------------SLLGRDTGCA----------------I-ICMSKK---------LDLL--------------------DANGR---------<br>MHHGNAAEFAKK---HGAGDEVA-------------SK-IVTIIHECEKKHEQ---------DGDECLRVLE----V---AKC----F---RTGIHE----LDWQPKV-<br>EVIVSEVLTEI-------------------
Spodoptera exigua	AAS46620.1	pheromone binding protein 1	SexiPBP1	---------------------------------MAGAKWRFVCVVFALYL--------------TSAALGS----QELMMKMTKGFTKV----VDECKA------------------------E------<br>LNAGEHIMQDMYNY-------WREDY-----------------QLINRDLGCM----------------I-LCMAKK---------LDLM---------------------EDQK---------<br>MHHGKTEEFAKS---HGADDEVA-------------KK-LVSIIHECEQQHAG---------IADDCMRVLE----I---SKC----F---RTKIHE----LKWAPNM-<br>EVIMEEVMTAV-------------------
Spodoptera exigua	AAU95537.1	pheromone binding protein 2	SexiPBP2	-------------------------------MAFCRSGTMSVLVVAASML--------------VVVQASQ-----DVMKNLAINFAKP----LDDCKK------------------------E------<br>MDLPDSVTTDFYNF-------WKEGY-----------------ELTNRQTGCA----------------I-LCLSSK---------LEIL--------------------DQELN---------<br>LHHGRAQEFAMK---HGADETMA-------------KQ-IVDMIHTCAQSTPDV--------AADPCMKTLN----V---AKC----F---KLKIHE----LNWAPSM-<br>ELIVGEVLAEV-------------------
Papilio xuthus	KPI94830.1	Pheromone-binding protein-related protein 3	PxutPBP3	----------------------------------MWTASLPVFFTLIVFG--------------RTEKEKP-----ELSDEIKEIIQHV----HNECVA------------------------K------TGVAEE---<br>DITNC-------ENGIF-----------------KEDTKLKRYM------------------YCLLEE---------ASLI--------------------DDEGN---------VDYDMMVSLIPE--------QYY----<br>---------DR-VHKMIFSCKHLDTPDKDKYQRVFDVHKCSYEKDPNVGSSLKTRCGCRNLAPSRSNDVD-GGSRRWRLDSAYVHWRRPPSSAELFF-------<br>--------
Papilio machaon	KPJ07512.1	Pheromone-binding protein	PmacPBP1	-----------------------------------MEKKIIFVVIVCLTT--------------CKTVYSS----QEIIQTMSINYMKG----LDTCKS------------------------E------<br>LNLPDVVDIEFAQF-------WREDY-----------------IISNRLTGCA----------------I-VCLSSK---------LDLL--------------------EPDGS---------LHHGNAADFAKK-<br>--HGADEAMA-------------QQ-LIDILHQCEQQYPD---------KMDACLYALQ----V---CNC----F---KTQIHK----LNWAPDV-ELIVGEVLAEI-------------------
Papilio machaon	KPJ07513.1	Pheromone-binding	PmacPBP2	-----------------------------------MCVVVVRTALLLLVVA--------------AAADDAP----SSIINEISKQFGSM----MLYCVQLVRYIHCCTLLLPFEKVESCGLWLQ---

		protein		---LYPQTGYFRDVLDF-------WNRDLN----------------ITGHTYLGCL----------------A-ACSLFK---------LQLR--------------------NRDGS---------<br>LNETNIMNFLRQ---NGAVENDA-------------AV-LLEVFKTCQNVSS-SE--------RKACAAGLK----T---MIC----F---RSQIYR----<br>LNWTPQFYRGPFENVLNGK-------------------
Cnaphalocrocis medinalis	AFG72999.1	pheromone-binding protein 1	CmedPBP1	-----------------------------------MGFLVKLVLLAMVV--------------GVQSSQD------VMKKVTVHFSKA----LETCKK------------------------E------<br>LDLPDAINTDFFNF-------WKEDY-----------------ELQNRLTGCA----------------L-MCMSSK---------LDLV--------------------DPEGK---------<br>LHHGNAHEYAKS---HGADDSVA-------------KQ-LVDLLHGCESSTAQ---------SDDDCSRVLG----I---AKC----F---KAEIHK----LKWAPDM-<br>EVVMAEVLAQV-------------------
Cnaphalocrocis medinalis	AGI37364.1	pheromone binding protein 2	CmedPBP2	---------------------------------MWAKTLMVVVTVVMMS--------------VNVESSQ-----TLLKDMTKNFLKA----YGQCQK------------------------E------<br>LGLPDSTATELMNF-------WKEGY-----------------EIKSREAGCA----------------I-MCLSKK---------LEVI--------------------DPEGK---------LHKGKTTEFIVA-<br>--HGTDEATA-------------HK-LIDILHACMQSVTP---------SEDHCLMSLQ----V---AMC----F---KAEIHK----LGWAPDT-ELLFEEMVAEMQ------------------
Cnaphalocrocis medinalis	AGI37367.1	pheromone binding protein 3	CmedPBP3	---MEVEMLP-----EGMKQLTGGFIKV----FEACKT------------------------E------LGLKDGMLTDMYHL----<br>---WREEY-----------------DQVSPDAGCM----------------F-GCMSKK---------LDLL--------------------DASGK---------IHHGNTKEYVMQ---NGGGEDLA-------<br>------AQ-LLSISQECEKQHEG---------VEAECARMLE----M---AKC----F---RSGIKR----VQWSPKM-EVVITEIIADV-------------------
Danaus plexippus	EHJ71307.1	pheromone binding protein 1	DplePBP1	---MAR--DILHI-------WEESY-----------------<br>DLNHDETGCL----------------V-LCAMVR---------LELL--------------------DQQGN---------MIVENTEGFIRA---NGGDDSMV-------------SF-<br>LIQLYSMCREKTSS---------ISNGCKAAIE----L---SKC----F---RAAIQQ----IGWVPDTTSLLVISYD----------------------
Danaus plexippus	EHJ71308.1	pheromone binding protein 3	DplePBP2	----------------------------------MNKSCVFVFALIFVN--------------VQKVQSN-----EVMKGITSSFFKV----LDECKR------------------------E------<br>LGLTDNVLTDLYYF-------WKQDH-----------------PLMHRDTGCA----------------I-VCMSQK---------LNLL--------------------DTIGK---------<br>LHHGNAQEFAIN---HGAGEQMA-------------KK-LVTMVHECEQQFME---------QEDSCLRALD----V---AKC----F---RTAMHD----VNWAPKF-<br>DIIVTEVLTEVK------------------
Plutella xylostella			PxylPBP	-------------------DFSLRKNKGLVLMVKMITKKLACLMMVLMCA--------------LKKVESS----ADVMKGLSENFGKA----LGDCKK------------------------E---<br>---LDLPDSIMTEFYNF-------WKDDY-----------------VLSDRSTGCA----------------I-ICLSSK---------LDLL--------------------DPDGN---------<br>LHHGNAKDFALK---HGADEGMA-------------GQ-LVGMIHECEKAAPD---------NPDACLKVLD----I---ANC----F---KKKIHE----LKWAPSM-<br>DVVVAEVLADV-------------------

Supplementary Table S4. Sequence details used in the alignment and phylogenetic analysis of CSP genes.

Species name	Accession number	Sequence definition	Abbreviation	Amino acid sequence
Bombyx mori	ABH88194.1	chemosensory protein 1	BmorCSP1	------------------------MKCLTIAALLFVAGLSIA--E---------------------------------KYTD-KYDNID---VDEILEN----------------RKLLVPYIKCVLDEGR-CTPDG-KELKAH-----------------------------IKDGMQT-ACAKCTDKQKVSARKIVKHIKQH--EAD--YWEQMKAKY----DPKDEFKEIYEGFLAGQN--
Bombyx mori	ABH88195.1	chemosensory protein 2	BmorCSP2	------------------------MKSVILICFLGVATVVIA--RP----------------------------------KT-PFDNIN---IEEIFEN----------------RRLLLGYINCILERGN-CTRAG-KDLKSS-----------------------------LKNVLEE-NCDKCSEDQRKSIIKVINYLVSS--EPE--SWNQLKSKY----DPEGKYLIKYEAKMESN---
Bombyx mori	ABH88196.1	chemosensory protein 3	BmorCSP3	------------------------MNSLIAFCLFAVLAVALA--RP-----------------------------DD-KYTD-RYDNVN---LDEVLSN----------------SRLLQPYIKCILDKDR-CAPDA-KELKEH-----------------------------IREALET-ECAKCTEAQKKGTRRVIGHLINN--ESK--SWNELTAKY----DPENKFTAKYEKELREIKA--
Bombyx mori	ABH88197.1	chemosensory protein 4	BmorCSP4	--------------------------MKVLIVLSCVLVAVLA--DD--------------------------------KYTD-KYDKIN---LQEILEN----------------KRLLESYMDCVLGKGK-CTPEG-KELKDH-----------------------------LQEALET-GCEKCTEAQEKGAETSIDYLIKN--ELE--IWKELTAHF----DPDGKWRKKYEDRAKAKGIVIPE---
Bombyx mori	ABH88198.1	chemosensory protein 5	BmorCSP5	-------------------------MKTVIVCLLALTAVALA--RP------------------------------E-QYTD-KYDTVD---LDQLISN----------------RRLLIPYVHCILEKGQ-CTAEG-KELKSH-----------------------------IKEALET-NCAKCTKAQKGGTEKMIGHLINH--EAE--FWEELKAKY----DPTNEFTKKYETELKRVTA---
Bombyx mori	ABH88199.1	chemosensory protein 6	BmorCSP6	--------------------------MKSLIVLSCLLAACLA------------------------------------ADLS-KYENFD---VEPIVTS----------------DRLLKAYINCFLDKGR-CTPEA-SDFKKA-----------------------------LPDTIAT-NCGKCTEKQKANVRKVIKVIQQK--HST--EWEKLVKKH----DPSGKHRADFDKFLLGS---
Bombyx mori	ABH88200.1	chemosensory protein 7	BmorCSP7	-------------------------MKGFYVLCFALFAAVYC--KE--------------------------------TYSS-ENDDLD---IEALVGN----------------IDSLKAFIGCFLETSP-CDAVS-GDFKKD-----------------------------IPEAVAE-ACGKCTPAQKHLFKRFLEVVKDK--LPQ--EYEAFKTKY----DPQGKHFDALLSAVANS---
Bombyx mori	ABH88201.1	chemosensory protein 8	BmorCSP8	--------------------------MKTILILCALVSVVVC--RP-----------------------------EE-YYSS-QYDNFD---VEQLVGN----------------LRLLKNYAKCFLDQGP-CTAEG-TEFKKR-----------------------------IPEALRT-KCAKCNPKQRHLIRTVVKAFQTK--LPD--LWEELAIKE----DPKGQYKHEFTAFINAMD--
Bombyx mori	ABH88202.1	chemosensory protein 9	BmorCSP9	--------------------------MRAVIFLYTCVFVVVG--QD----I--N-----------------AMMSMP-KYDE-RYDYLD---VDDIFRN----------------KRLVRNYVDCLINAQR-CTPEG-KALKRI-----------------------------LPEALRT-KCIRCTERQKRTSVKVIRRLKNE--YPE--EWAKLASRW----DPTGDFTRYFEDYLAKEHFNTIPGSGPTVNVLSLQTTPPPPPPPPSRPASVFTNPPP---PVMSTSPRPVVLNRFRR-------------------------
Bombyx mori	ABH88203.1	chemosensory protein 10	BmorCSP10	-------------------------MKILIIVVMACVAVTWA--RP------------------------------ESTYTD-KWDNIN---VDEILES----------------NRLLKGYVDCLLGKGR-CTPDG-KALKET-----------------------------LPDALEH-ECVKCTGKQKSGADKVIRHLVNK--RPD--LWKELAVKY----DPDNIYQARYKDKID---

Bombyx mori	ABH88204.1	chemosensory protein 11	BmorCSP11	-------------------------MKLTSFLLVGMAMVSAE------------------------------------FYSS-RYDDFD---VKPLVEN-----------------<br>DRILQSYTNCFLDKGP-CTPDA-KEFKKV------------------------------IPEALET-TCGKCSPKQKQLIKTVIKAVIER--HPE--AWEELVNKY----<br>DKDRKFRPSFDKFINEDD--
Bombyx mori	ABH88205.1	chemosensory protein 12	BmorCSP12	------------------------MFMLFIISFIIVPVLKCCG--TE------------------------------TS-TYTT-QYDEVD---IKEIMGN-----------------<br>ERLLVAYIGCLLDKNP-CTPEG-KELKRN------------------------------IPDALQS-DCSKCSDKQRENADAWIEFMIDN--RPE--DWTKLEER---<br>--
Bombyx mori	ABH88206.1	chemosensory protein 13	BmorCSP13	--------------------------MKLLLVFLGLFLAVLA--QD--------------------------------KYEP-IDDSFD---ASEVLSN-----------------<br>ERLLKSYTKCLLNQGP-CTAEL-KKIKDK------------------------------IPEALET-HCAKCTDKQKQMAKQLAQGIKKT--HPE--LWDEFITFY-<br>---DPQGKYQTSFKDFLES--
Bombyx mori	ABH88207.1	chemosensory protein 14	BmorCSP14	-------------------------MKSSLFCVLVLTVVVSSSRQQ--------------------------------SYP--RNDNIN---INAILQN-----------------<br>DRILLGYFKCVMDRGP-CTKDG-KTFKRA------------------------------LPEALPT-ACARCSNKQKAAFRTLLLAIRAR--SEP--SFLELLDKY--<br>--DPSRSNRELLYTFLATGL--
Bombyx mori	ABH88208.1	chemosensory protein 15	BmorCSP15	-----------------MIENFYSKCTISKSVLFLCLIFLPYA--LN------------------------------QK-YYDS-RYDYYD---IDHLVQN-----------------<br>PRLLKKYLDCFLGKGP-CTPIG-RLFKQV------------------------------MPEVITT-ACAKCTPTQKRFARKTFNAFRRY--FPE--TLMELRRKF---<br>-DPESKYYDAFEKVITNA---
Bombyx mori	ABH88209.1	chemosensory protein 16	BmorCSP16	----------------MIEWKRFKILHFLSYLGLLVLVVVCAA------------------------------------QQNRPQVTDTA---LDEALND-----------------<br>KRFIQRQLKCALGEAP-CDPIG-KRLKTL------------------------------APLVLRG-ACPQCSPQETKQIQKTLSYVQRN--FPQ--HWAKLVRQY-<br>---AG--
Helicoverpa armigera	AEB54579.1	CSP5	HarmCSP1	------------------------MRTFVVVCLLGLVAVTLA--RP------------------------------ESKYTS-KYDNIN---LDEILAN-----------------<br>QRLLVPYLKCILEEGK-CTPEG-KELKSH------------------------------IREALEE-DCAKCTENQRKGTRKVLAHLINH--EEG--<br>YWNRLKAKY----DPESKYTAKHEQELRELKH---
Helicoverpa armigera	AEX07265.1	CSP2	HarmCSP2	--------------------------MKVVLLTLCFALGVLA--QD--------------------------------QYES-ANDNFD---ISEVIGN-----------------<br>DRLLHAYANCLLNKGP-CTPEV-KQVKEK------------------------------LPEALET-RCAKCTDKQKQMGKALAQEVKKN--HPD--<br>IWKQLVAMY----DPQGKYQQAWKDFLQE--
Helicoverpa armigera	AEX07266.1	CSP3	HarmCSP3	------------------------MNADWFLIFTLITVVSSD------------------------------------FYNS-KYNCFN---VQPLLEN-----------------<br>DRILLSYTKCFLDQGP-CTPDA-KDFNKV------------------------------IPEALET-TCGKCSPKQKLVIKTVIKAVISR--HPD--AWDQLTEKY----<br>DKDKKYKDSFDKFLA---
Helicoverpa armigera	AEX07267.1	CSP6	HarmCSP4	-------------------------MKADCFLFVTLIAVVAA-----------------------------------D-FYNS-KYDSFD---VQPLLEN-----------------<br>DRILLSYTKCFLDQGP-CTPDA-KDFKKV------------------------------IPEALET-TCGKCSPKQKQLIKTVIKAVISR--HPD--AWDQLTEKY----<br>DKDQKYKESFDKFLAEQD--
Helicoverpa armigera	AEX07268.1	CSP7	HarmCSP5	------------------------MKVFVVLSVLIAFTAAASL--TP---------------------------------AELD-LAEAFD---YEALFSN-----------------<br>DEQRKLVFDCILGKGE-CG-DY-QKMAEI------------------------------SRKVLES-KCADCNPKQKAKYETVLKTIQTK--YEP--<br>FYNELLKNV----AAKKE---

Helicoverpa armigera	AEX07269.1	CSP4	HarmCSP6	-------------------------MNSLIVFCVLSLAALTIA--RP------------------------------DGATYTD-KYDNVD---LDEILGN-----------------RRLMVPYIKCMLDQGK-CAPDA-KELKEH------------------------------IKEALEN-ECGKCTEAQKKGTRRVIGHLINH--EAD--FWNELTAKY----DPERKYTTKYEKELKEVKA--
Papilio xuthus	BAF91711.1	chemosensory protein	PxutCSP1	-------------------------MNSLLLFSLLTLFVVAFA--NE---------------------------------QYTD-RYDNIN---IDEILSN-----------------KRLLTSYIKCILDKGR-CTPEG-KELKLH------------------------------IKDGMQN-SCSKCTDFQKNGARKVVKYIRAN--EKE--SWEEMKKKY----DPKDEYKEKYEAFLAADN--
Papilio xuthus	BAF91712.1	chemosensory protein	PxutCSP2	-------------------------MKLFMVCALLCVAAVAWG--KP-------------------------------ASTYTD-KWDYIN---VDEILES-----------------QRLLKGYVDCLMDRGR-CTADG-KTLKET------------------------------MPDALEH-ECSKCTEKQKESSDKVIRFLINK--RPE--LWKELATKY----DPDNVYQQRYKDKIEAVKEH--
Papilio xuthus	BAF91713.1	chemosensory protein	PxutCSP3	---------------------------MKTLVVLACVLLSVYA--AD---------------------------------KYNS-KYDNFD---VETLITN-----------------ERLLKSYINCFLDKGR-CTAEG-TDFKKA------------------------------LPEAIET-TCGKCTEKQKLNIRKAIRAIQQK--YPG--QWEDLVKKN----DPSGKHRANFDKFIQGS---
Papilio xuthus	BAF91714.1	chemosensory protein	PxutCSP4	-------------------------MNTFLMVCLLALVAAVSA--D----------------------------------QYTD-RYDNVD---LDEILSN-----------------RRLLVPYLNCILEEGK-CSPDG-KELKSH------------------------------IKEALEN-NCGKCTETQKSGSRKVIGHLINN--EKE--YWGKLTAKY----DPERKYVTKYESELRKIAA--
Papilio xuthus	BAF91715.1	chemosensory protein	PxutCSP5	------------------------MKLLPILVIAACVVMVKAG-------------------------------------TYTD-RYDSMN---VDDVIAN-----------------KRLFIAYVKCILNKGR-CTPEG-KELKSH------------------------------ITEALQS-GCDKCTARQRRSIRKAIKHLIHK--ENN--YWNQLVNMY----DPNKMYSKMYERELGTI---
Papilio xuthus	BAF91716.1	chemosensory protein	PxutCSP6	---------------------------MKTIILLCTLAAAALA--AP------------------------------AD-TYNS-QYDNFD---ATELVGN-----------------TRLLKSYGRCFLGQGP-CTAEG-SDFKKT------------------------------IPEALRT-TCAKCTPKQRELVRVVVRGFQTK--LPE--IWEELVKQQ----DPKGEFKEAFDRFLNSSD--
Papilio xuthus	BAF91717.1	chemosensory protein	PxutCSP7	---------------------------MKLIVLVLCVTALAYA--ED---------------------------------KYED-IEDNFN---LQELLEN-----------------DRLLTGYIKCLLNKGP-CTPEV-KKIKEK------------------------------LPEALAT-NCAKCTDKQKQMGKVLVKQVKKA--HPE--LWDELKNLY----DPQGKYQKEFQQFLSD--
Papilio xuthus	BAF91718.1	chemosensory protein	PxutCSP8	-------------------------MKSKAALVLMCVLAAALA--E----------------------------------SYSD-KYDNID---LQEIADN-----------------DRLLDAYANCLLEKGK-CSPEG-KELKGH------------------------------MKDAIET-GCEKCTDAQKKGTNFMIDHLIRK--KPE--IWNQLANKY----DPTGKWRKVYEDRAKEHGIVIPH--
Papilio xuthus	BAF91719.1	chemosensory protein	PxutCSP9	-------------------------MVSRNLMILCCLVVAVVA--KP---------------------------------TYTD-KYDNID---LEEFKEN-----------------KRLLLAYVDCILDKGK-CTAEG-KALKDN------------------------------LLDATET-GCEKCTEKQKEGSYEMIEHLIKN--EPE--IWNELCAKY---DPTGKWRKEYEEKAKAKGIKIPQKISTK---
Papilio xuthus	BAF91720.1	chemosensory protein	PxutCSP10	------------------------MQINHIVIALCALAATCLA--QA---------------------------------QTDRPPVSDTA---LEEALND-----------------KRFIQRQLKCALGEAP-CDPIG-KRLKTL------------------------------APLVLRG-ACPQCTPQETKQIQRTLSYVQRN--FPQ--QWAKIVRQY----SG--

Papilio xuthus	BAF91721.1	chemosensory protein	PxutCSP11	--------------------------MKLILMILASTIALVQG--DA-----------------------------------TKQRYDAFD---IQTALQN-----------------<br>DDIILSLINCFGDTTP-CSPEM-KAFKND------------------------------IPTALQT-ACGKCSDRQREVIRHVIRTVMKK--YPD--AWTYLIDKY----<br>DPENKYRDGFYQFIGQDD---
Ostrinia furnacalis	BAV56805.1	chemosensory protein 1	OsfuCSP1	-------------------------MISTKYLIVLCCVAAAVA--RP------------------------------SD-KYTD-KYDNLN---IQEILEN-----------------<br>KRLLKAYVDCVMGQGK-CSPDG-KELKEH------------------------------LQEAIET-GCAKCTEAQEKGAYTAIEYLIKN--ELD--<br>IWKQLAAKF----DPEGKWRKTYEDRARANGIVIPE--
Ostrinia furnacalis	BAV56806.1	chemosensory protein 2	OfurCSP2	---------------------------MKTIVALCALVAVALA--RP------------------------------ED-TYST-AFDSFN---AQELVDN-----------------<br>IRLLKNYGKCFLDQGP-CTPEG-SDFKKK------------------------------IPEALKT-DCGKCTPKQRELIKTVVHGFQSK--LPD--<br>MWAELVKKH----DPEGQYTESFDAFLNSK--
Ostrinia furnacalis	BAV56807.1	chemosensory protein 3	OfurCSP3	-------------------------MKTFILICLSALVMVSSA-------------------------------------DKLD-DLLNTD---MEKLLAD-----------------<br>DAVRKQVVGCMTDELP-CG-DY-QAYKDM------------------------------LPDLIAT-NCGKCTPEQKKRYEEINKFVLEK--YPN--<br>EYNAVVSKY----RPKTE--
Ostrinia furnacalis	BAV56808.1	chemosensory protein 4	OfurCSP4	-------------------------MKTFVLLALSLVVAVAYA--RP-------------------------------GAQYTD-KWDHIN---VDEILES-----------------<br>QRLLRGYVDCLLDKGR-CTPDG-KALKET------------------------------LPDALEH-DCSKCTEKQKASSDKVIRHLINK--QPD--<br>YWKELSAKY----DPNNIYQDKYKDKIEEVKSKN--
Ostrinia furnacalis	BAV56809.1	chemosensory protein 5	OfurCSP5	--------------------------MHPQHFCMIVMVTTAAA--D----------------------------------FYSA-KYDDFD---IQPLLEN-----------------<br>DRILQGYTKCFLDQGP-CTPDA-KDFKKV------------------------------IPEALET-SCGKCTPKQKILIKKVIRAVMER--HPD--SWKELEDKF---<br>-DKDKKFRDSFNKFLEEKD---
Ostrinia furnacalis	BAV56810.1	chemosensory protein 6	OfurCSP6	---------------------------MKLVIISLCLAAAVVA--QE---------------------------------KYDS-IDDNFD---ISEVLNN-----------------<br>ERLLNSYTKCLLDKGP-CTPEV-KKVKDK------------------------------LPEALAT-RCAKCTDKQKQIGKQLAKEVKAK--RPD--<br>LWKELVAHY----DPEGKYQEAFQDYLKP---
Ostrinia furnacalis	BAV56811.1	chemosensory protein 7	OfurCSP7	--------------------------MKTFAICLLALVAVVSA--YP-------------------------------QAKYTD-RYDSIN---LDEIVGN-----------------<br>RRLLVPYIKCILDQGK-CSPEG-KELKSH------------------------------IKEALEN-YCAKCTETQRDGTRKVIGHLINN--ESE--YWNQLTAKY--<br>--DPQRKYVVKYEKELRTVS---
Ostrinia furnacalis	BAV56812.1	chemosensory protein 8	OfurCSP8	------------------------MKLVAFIPTFTYLLLGANA--EE------------------------------SP-TYTT-KYDGVN---LDEILEN-----------------<br>DRLLTSYVNCLLETGP-CTPDG-KELKNN------------------------------LPDAIQN-DCKKCSERQREGADQVMEYIIDH--RPD--<br>DWEKLEKKY----NSDGSYKKKYLERKEARNQSNSAEKSQENDSKSKE--
Ostrinia furnacalis	BAV56813.1	chemosensory protein 9, partial	OfurCSP9	----------------------------MKFLVLSAVLALALA--D----------------------------------SYKS-DYDSLD---IAPIVND-----------------<br>PEALSKLTACFLDKGP-CTPIA-ADFKTY------------------------------LPDATET-ACSKCNTAQKQKLKLYLQKVKET--SPD--DLAALKAKY--<br>--DPDSKHVDALIAALKE---
Ostrinia furnacalis	BAV56814.1	chemosensory protein 10	OfurCSP10	---------------------------MKTIMLVAFLVGLAMA--DE---------------------------------KYTS-ENDNFD---VEALVNN-----------------<br>TEELQKFSGCFLDKND-CDAVS-GDFKKD------------------------------IPEAFQQ-ACAKCTDAQKHLFKRFLNGLKEK--LPQ--<br>DFEAFKKKY----DPEDKFFAALDKAINA---

Ostrinia furnacalis	BAV56815.1	chemosensory protein 11	OfurCSP11	------------------------MRHIIILLAVVALVTQSFA--DE-----ETEKKEEKKEEKKE-------EQTDEK-KYTD-RFDDIN---FEEIIAN-----------------RRLLVPYLKCVLDKGR-CTPEG-KELKAH------------------------------VKDAMQT-ACEKCTDKQKTGARKVVNHIRDN--EKE--YWEELINKY----DPKGEFKSIYEPFLAAKE--
Ostrinia furnacalis	BAV56816.1	chemosensory protein 12	OfurCSP12	---------------------------MKFLVVLSAVLAVALA--RP-------------------------------D-SYKT-DHDGLD---IEGIVNN-----------------PEALAKVTACFLEKAP-CTPIA-AEFKSV------------------------------LPDATET-ACSKCTAAQKHMLKLYLLKVRET--APD--DLKALKTKY----DPDSKHIDALIAAIKDA--
Ostrinia furnacalis	BAV56817.1	chemosensory protein 13	OfurCSP13	---------------------------MRAVVFLSCLVVVLAA--D----------------------------------KYNS-KYDNFD---VETLISN-----------------DRLLKAYINCFLEKGR-CTPEG-ADFRKA------------------------------LPEAVET-TCAKCTEKQKNNIRKVIRAIQQK--HPK--QWEELVKKT----DPSGKHRAGFDKFIQSN--
Ostrinia furnacalis	BAV56818.1	chemosensory protein 14	OfurCSP14	-----------------------MWIQLAILATFVSIVITEMG--PP-GIERTFS------------------DGVTSR-GYRV-VYGDEDLTVINEVVGNMEKNDILKAKASLNEAIQPLPAGDVKCLMSADRYCSVEM-RKVKGV------------------------------LIQALKN-DCEKCSNTEKDTAGRVAASMMTY--DPV--GWKLFLTRY----DGLSKIQRILG--
Ostrinia furnacalis	BAV56819.1	chemosensory protein 15, partial	OfurCSP15	---------------------------MRAVLLLCACAAAVCG--QN----LDSN------------------RMARMP-KYDE-RYDYLD---VDALFNS-----------------KRLVRNYVDCLINAQR-CTPEG-KQLKRI------------------------------LPEALRT-KCIRCTERQKKTAVKVIKRLKYE--FPE--EWAKLSSRW----DPTGDFTRYFEEFLANESFNTISGSADGNDAAGPSSIPPLPPVPPRLPAAPPSTPPPLPVEPVSTSPKPVILNRFGDDGELMMGSPSSAALTPRPSTARPPLS
Ostrinia furnacalis	BAV56820.1	chemosensory protein 16	OfurCSP16	---------------------MSHRKVLVLSHLMVFLCVQCFA-------------------------------------KLHN--YDNFD---METLLLN-----------------TTRSRALFECVRDETK-CANKEDKEMKDD------------------------------IFEMVTT-SCANCTAKEKQKFGDAMKALHRSMGESQ--IITMFINKM------TNMFQGGLSDTEKTT--
Ostrinia furnacalis	BAV56821.1	chemosensory protein 17	OfurCSP17	------------------------MQTTLVLLLVVAACAYAAE--AP------------------------------RP------QVTDTA---LEDALND-----------------KRFIQRQLKCALGEAP-CDPIG-KRLKTL------------------------------APLVLRG-ACPQCSPQETKQIQRTLSYVQRN--YPQ--QWAKIVRQY----AG--
Ostrinia furnacalis	BAV56822.1	chemosensory protein 18	OfurCSP18	-------------------------MQKLIILALVCTMGWSVVVAAP--------------------------------------QMTDAQ---LDQTLTD-----------------RATMQRHLRCALQEGP-CDPVG-KRLRIL------------------------------APLVLRG-TCRQCTPQETRQIRYTLAFVQRN--YPW--EWAKLIRQY----G--
Ostrinia furnacalis	BAV56823.1	chemosensory protein 19	OfurCSP19	---------------------------MKTLLFAITLAALACCARAQ---------------------------------VYTD-RYDTVN---LDDVLAN-----------------KRLTVAYIKCMLDKGG-CTSEG-RELKSH------------------------------IAEALQN-GCAKCTKAQREGMRRVIKHLIQH--EKG--YWQELVEKY----DPKRVYTQKYENELNSL--
Cnaphalocrocis medinalis	AIX97823.1	chemosensory protein	CmedCSP1	-------------------------MISTKYLLVLCCVAAALA--RP-------------------------------NDKYTD-KYDNLN---LQEILEN-----------------KRLLKAYVDCVMGRGK-CSPEG-KELKEH------------------------------LQEAIET-GCEKCTEAQEKGAYTAIEYLIKN--ELD--IWRELAAHF----DASGKWRKKYEDRARANGIIIPE--

Cnaphalocrocis medinalis	AIX97824.1	chemosensory protein	CmedCSP2	-------------------------MRPIVAMFLLAIIGLAAA--AP---------------------------------AEKTDDSDEVN---VDEILAN-----------------RRLLLPYIKCALEQGK-CSSSG-KKVKDH------------------------------IKKSLQN-DCETCTEKQKKDTNAVFKHLINK--END--YWNQLIAKY----DPQRQYAPKHEKLYLGKA--
Cnaphalocrocis medinalis	AIX97825.1	chemosensory protein	CmedCSP3	-------------------------MKAIILFGLTTLVAVAWG--RP-------------------------------GTLYTD-KWDHIN---VDEILES-----------------QRLLRGYVDCLLDKGR-CTPDG-KALKET------------------------------LPDALEH-NCSKCTEKQKSSSDKVIRHLINK--QPD--YWKELSTKY----DPQNIYQERYKDKIDDVKAKA---
Cnaphalocrocis medinalis	AIX97826.1	chemosensory protein	CmedCSP4	------------------------MMKFATALVLAVCVGLAVG--E-----------------------------------TYST-ENDDFD---IEALVKN-----------------PEEFQKFSGCFLDKNE-CDAVS-GDFKKD------------------------------IPEAFEQ-ACAKCTDAQKHLFNRFLSALKDK--RPQ--DFEDFKKKY----DPEAKYYAALEKAVAKA---
Cnaphalocrocis medinalis	AIX97827.1	chemosensory protein	CmedCSP5	------------------------MKTQIIFLCVLGVIALVSC--AP------------------------------HQ-SYAN-QPETTP----EEIMRV-----------------ESQLP----CVLNQGP-CSELG-LRIKMV------------------------------LPEILTTRKCSTCTPEENAKVGRILYIMHEK--FRH--HLITLNNIY----GKKQQSSNFHHSN---
Cnaphalocrocis medinalis	AIX97828.1	chemosensory protein, partial	CmedCSP6	---------------------------MRVLVILCVCACVAYG--QE-------E------------------RISRMP-KYDE-RYDYLD---VDALFNS-----------------KRLVRNYVDCLISAQR-CTPEG-KQLKRI------------------------------LPEALRT-KCARCTERQK--
Cnaphalocrocis medinalis	AIX97829.1	chemosensory protein	CmedCSP7	--------------------------MRSWLVLVLAVVVSCSA-------------------------------------QHYN-RYDNFN---ADSIIQN-----------------DRVLLAYYKCVMDKGP-CTKDG-KNFKRV------------------------------LPETLST-ACGRCSAMQKLVVRKLLLGIRSK--SEP--RFLELLDKY----DPERSNREALYNFLVTGN--
Cnaphalocrocis medinalis	AIX97830.1	chemosensory protein, partial	CmedCSP8	-----------------------------NLFLFTCVLFVVSG--TQ------------------------------EE-LYDR-KYDYFD---IETLVQN-----------------PRLLKKYMDCFLDKGP-CTPIG-RVFKLA------------------------------LPEVIST-SCSKCTPAQ---
Cnaphalocrocis medinalis	AIX97831.1	chemosensory protein	CmedCSP9	-------------------------MQKILCIVFLSSVVSTIA-------------------------------------YPAPQMTDGQ---LEQTLAD-----------------RSTMQRHLRCALQEGP-CDPVG-RRLRIL------------------------------APLVLRG-ACPQCSVQETRQIQRTLAYVQRN--YPW--EWAKIVRQY----G--
Cnaphalocrocis medinalis	AIX97832.1	chemosensory protein	CmedCSP10	---------------------------MKVLILTLCLAAAALA--QE---------------------------------KYDS-VDDNFD---ISEVLDN-----------------ERLLNSYAKCLLDKGP-CTPEV-KKVKDK------------------------------LPEALQT-RCAKCTDKQKQIGKKLAQEVKKK--RPD--LWKDLVAHY----DPQGKYQESFQDYLKP---
Cnaphalocrocis medinalis	AIX97833.1	chemosensory protein	CmedCSP11	-------------------------MQIQVLLLVVAAAACAYA--AE---------------------------------TAHP-TVSDTA---LDDALND-----------------KRFIQRQLKCALGEGP-CDPIG-KRLKTL------------------------------APLVLRG-ACPQCSSQETKQIQRTLSYVQRN--YPQ--QWAKIVRQY----AG---
Cnaphalocrocis medinalis	AIX97834.1	chemosensory protein	CmedCSP12	-------------------------MKLVIFACLVALAAVAHA--GP-------------------------------QRGYTD-VYDSID---INEVLGN-----------------RRLLLPYLHCVLGEGK-CTPPG-KELKSH------------------------------IKEALET-QCAKCTPAQRTGTRKVIAHLINH--EAE--YWNKLTAKY----DPTGQFTKKYENELRVIA--

Cnaphalocrocis medinalis	AIX97835.1	chemosensory protein	CmedCSP13	------------------------MKILAVTLVLTTCVTINTA--DE-------------------------------S-TYTT-KYDGVD---INEVLGN-----------------ERLLTSYVNCLLDLGP-CTPDG-KELKNN------------------------------LPDAIQN-DCKKCSDRQREGADQVMDYIIDH--RAE--DWAKLEKKY----NSDGSYKKKYLERKQAKEAKEAKESKEHDESQEKTE---
Cnaphalocrocis medinalis	AIX97836.1	chemosensory protein	CmedCSP14	------------------------MRST-ILLTILAIVALSLA--EDTDQAKDTKSKESGKESDKESGNESGKESGKEE-TYTD-RFDNIN---VDEIVAN-----------------RRLLVPYLKCALDKGR-CTPEG-KELKIH------------------------------IQDAMQT-ACKKCTEKQKTGARQVVNHIKEK--EPL--YWEELLAKY----DPKNELKPIYEPFLAGKDK---
Cnaphalocrocis medinalis	AIX97837.1	chemosensory protein	CmedCSP15	
Cnaphalocrocis medinalis	AIX97838.1	chemosensory protein	CmedCSP16	---------------------------MLYLTVLLVLVGQCYA-------------------------------------KLHN--YENFD---LESFLSN-----------------EAKAKAFLGCVSDDSK-CGSQGDLEMKND------------------------------IVEMMMT-SCAGCTEKEKEKYKEGLAVLQKSVGDPQTAMFNQFANLFLWGTDPEGKNQ---
Cnaphalocrocis medinalis	AIX97839.1	chemosensory protein	CmedCSP17	---------------------------MKFLVVLSMVFAVAVA--D----------------------------------TYKT-THDSID---VEAVVTN-----------------PDSLKAFTGCFLDTGA-CNEVA-ASFKKV------------------------------LPEATEQ-ACAKCTPAQKHMLRRYLEEVKKT--SPE--DFAALNKKY----DPEGKYVEALRAAIANA---
Plutella xylostella			PxylCSP1	HDAKVRIISCDLLAKPYTPYPNFSIMQKLTLACLLVAVAAAAA--RP------------------------------NDSHYTD-RYDNVN---LDELISN-----------------RRLLVPYVKCVLDQGK-CSPDG-KELKEH------------------------------IQEALEN-NCGKCTDKQREGTRKMIGHLINH--EQE--FWDQLIAKY----DPERKYVSKYEKELKEVKA---
Plutella xylostella			PxylCSP2	--MDRKIYLPPIVKKFCVKVVYSQIMKNVVVLCLFCSAWLGLV--SA----------------------------------YPVTQMTDAQ---LDKTLSD-----------------KATMQRHLKCAMGEGP-CDPVG-RRLRTL------------------------------APLVLRG-SCPQCSPQEARQIRRTLAHVQRN--YPW--EWAKIIRQY----G---
Plutella xylostella			PxylCSP3	-------------------YSISTMKAAAFIALFLIGKAVCED--KP---------------------------------TYTT-KYDNID---LDEILSS-----------------ERLLTGYVNCLLDQGP-CTPDG-KELKRYLETMCRRLDWICVDPSEIIRITVLESDLNTLPDAIDN-DCRKCTQKQKEGSDRVMGYIIEY--RPN--DWAKLEKKY----LSDGSYKKKYLEKKNASENNGDSKSTEAKNKDDEEKKSKGDGEEK--
Plutella xylostella			PxylCSP4	----------------------MSKMKLFVALCFVTLVAYSSA--RP-------------------------------NGSYTD-RYDNLD---LDEILNN-----------------SRLRVPYVKCLLGKGK-CSPDG-KELKSH------------------------------VREALEN-QCGKCTPAQQAGTRKVIGYLINN--EAG--YWQELVALY----DPQRKYVKQYETELRKVSG---
Plutella xylostella			PxylCSP5	------------LKTVEKYIFKTPLLTMKVLVILAAAAAVAVA--RP------------------------------GD-LYDD-SKSNLD---IDELVSN-----------------ERLLKGYAHCFLEKGP-CTPEG-NNIKKI------------------------------IPEALEN-VCKKCTPKQRVMVRKMIAAFKEK--LPA--EWSELAKTY----DSEGKYKENVKTFLAQSD--
Plutella xylostella			PxylCSP6	-----MEIKSDFGVSVQCVRAIRMQFSITHLLVLSALVAVCWA--QA---------------------------------KETKPRVSESA---LEEALND-----------------KRYIQRQLKCALGEAP-CDPTG-KRLKTL------------------------------APLVLRG-ACPQCTPQETKQIQRTLSYVQRN--FPQ--EWAKIVRQY-

		---AG---
Plutella xylostella	PxylCSP7	----GGTALVLARSILLRYRIKGQRLKMKVAIIVSMLVVAACA--QD---------------------------------KYKSDLEGDFD---VTELLNN---------------<br>--ERLLLSYTRCLIDKGP-CTPEV-KAVKDK------------------------------LPEALAT-KCAKCTDKQKELGKKLAVELKRT--HPA--<br>VWAQLVAKY----DPQGQHQAAFQEFLTKQ--
Plutella xylostella	PxylCSP8	----------------MNKNFFHSDIKMRAVFVLCACVWGAVG--QD----I--N------------------SMRTMP-KYDS-RYDYLD---VDAILDS-----------------<br>KRLVRNYVDCLIAIKP-CTPEG-KALKRI------------------------------LPEALRT-KCIRCTDRQKRTAVKVIKRLKYE--YPS--EWAKLSCRW---<br>-DPSGDFTRFFEEFLAKEAFNTISGTGNELPGTLTSQATPAPPPPPP---

Supplementary Table S5. The sequences and target of the primers used in qRT-PCR and RT-PCR.

	PCR target	Forward primer sequence	Reverse primer sequence
**qRT-PCR**	ALR1	GGCGTCACTATATAACAATGAACACCT	TCAGTGACCCGAGCACATCACGTTG
	ALR5	AGGATCTTAACCAGGATTCTATTGCG	ACAGCCGAGTAGGTAATAACCGTTT
	DES7	AGTTTCAGTTAATTCACTAAACCGCGTCC	GGCTCTGAAAATTTTGTGCGGAT
	DES3	GTCCACAACTTGCTTTACCGGACT	CTTCTACCACTGACGCTTGCTC
	DES8	AGATGGCGTATGTTTGTAGCCTT	CTCACTTTTCAAAGCATGCGCTCA
	FAR5	CGGTGAATTGAACGAAACATTGCAT	ACTTGCGATTCTTCCGGCATT
**RT-PCR**	CSP1	TATGACAACGTGAACCTTGACGA	CACAGACTACGCTTTATAACCAG
	CSP2	TGCCATTTACAATAATTTATGTGATGC	TGCCCTACTGCTAATGGAC
	CSP3	TGAAGACAAGCCAACTTACACC	TTCTGTACTTTTGCTATCGCCAT
	CSP4	TAGCCATTTTGCTTGTAGATCGTT	ACTATTTCTCGCTATATAGGATCGG
	CSP5	AGTAACAATTTAAAGGCAAATCCTG	AAATCATTCCCGAGGCACT
	CSP6	ACGTGAAGTGATTATTCTTATTGAGT	GAACCCTGTCCTACGTCCA
	CSP7	ACTGCGCTTGTCTTAGCTC	CTTCTGCTTGTCCGTGCAT
	CSP8	GGACAACATCACGCAACACC	TAGCGTCCACATCCAAATAGTCG
	OBP1	CTTTGGCATTTATCCTTATCTGGT	GAAAATCTAAGCCGGAATTCAGT
	OBP2	TGCATTATTCAAGCCTTTTCGT	TAAAGTCCAATAACCACCGGAGA
	OBP3	GCTAATGTTAAACACGCTCACC	GAGTCAGCCATATCCTTTCCG
	OBP4	GGCAGTATCCTAAGAATCCTGT	TTTTGTTTCTCCTCGCTGTCC
	OBP5	TTCCTGAAGACTTTAAGGCCAA	GAAATAGAAGCACCTTAATGCAA
	OBP6	GTTTCTTTGACACACGGCAAC	TGCCCTTCAGAATCGATACCAA
	OBP7	CGGACTCTAAACACAACTACACC	ATCTCGCTCTATCACATATGCTT
	OBP8	ACAACAGCTATTAAACAGTCGCAAA	TACGTCAAGTGCTACCACGAG
	OBP9	GCTCACTTCCCGTACTCCATCCG	ACGTGAGTTTCCCTTATAGTTGGTC
	PBP	AGGACTATCTGAAAATTTCGGTA	TCTCGGTAAGAAATTCTCGT
